# Multiparameter quantum-enhanced adaptive metrology with squeezed light

**DOI:** 10.1038/s41467-026-73069-1

**Published:** 2026-05-14

**Authors:** Giorgio Minati, Enrico Urbani, Nicolò Spagnolo, Valeria Cimini, Fabio Sciarrino

**Affiliations:** Dipartimento di Fisica, Sapienza Università di Roma, Roma, Italy

**Keywords:** Quantum metrology, Quantum optics

## Abstract

Squeezed light enables quantum-enhanced phase estimation, with crucial applications in both fundamental physics and emerging technologies. To fully exploit the advantage provided by this approach, estimation protocols must remain optimal across the entire parameter range and resilient to instabilities in the probe state. In this context, strategies that rely on pre-calibrated squeezing levels are vulnerable to degradation over time and become sub-optimal when experimental conditions fluctuate. Here, we develop an adaptive multiparameter estimation strategy for ab-initio phase estimation, achieving sub-shot noise limit precision in the full periodicity interval [0, *π*), without relying on prior knowledge of the squeezing parameter. Our approach employs real-time feedback to jointly estimate both the optical phase and the squeezing level, ensuring robustness against experimental drifts and calibration errors. This self-calibrating scheme establishes a reliable quantum-enhanced sensing framework, opening new routes for practical scenarios and scalable distributed sensor networks using squeezed light.

## Abstract (web summary)

Squeezed light can improve optical phase measurements but usually needs careful calibration. Here, authors demonstrate a self-calibrating adaptive method that jointly estimates phase and squeezing, achieving quantum-enhanced precision from scratch across the full phase range.

## Introduction

Quantum-enhanced optical phase estimation using non-classical states of light^1^ has attracted renewed interest in recent years, driven both by pioneering applications in the current gravitational wave detectors^2–5^ and by its impact on emerging areas such as imaging^6^, spectroscopy^7,8^, and distributed sensing^9–11^. In this context, maximally entangled probes, such as *N*00*N* states, provide, in principle, the highest phase sensitivity permitted by quantum mechanics for a given photon number^12,13^. However, their practical use is limited by the brightness of single-photon sources^14^ and by the detrimental effect of photon losses, which rapidly degrade their advantage^15^. To date, unconditional demonstrations of sub-shot noise limit (SNL) performance with *N*00*N* states have been restricted to the two-photon regime^16^, making scalability to higher dimensions a major open challenge. As a result, the realization of quantum sensors based on such maximally entangled states remains difficult in practice.

Different routes have been explored, such as multi-pass strategies, where the same phase shift is coherently sampled multiple times^17,18^ and photon total angular momentum encoding^19^. In contrast, squeezed light provides a more scalable route to phase estimation beyond the standard quantum limit (SQL)^20,21^, where the advantage arises from nonclassical fluctuations at fixed mean photon number, rather than by increasing the effective interaction through repeated sampling, while mitigating some of the key limitations associated with maximally entangled probes. Its effectiveness has already been demonstrated in the most recent upgrades of gravitational wave interferometers, which have shown the possibility of operating below the SQL for broadband signals, marking a milestone in the practical application of quantum resources for precision measurements^22–25^

Although squeezed light enables sensitivities below the SQL^26–29^, this is obtained at the expense of increased noise in the conjugate quadrature. Consequently, this enhanced precision is contingent upon a specific value of the optical phase, posing challenges for generic measurement strategies that necessitate the estimation of the phase as a fully unknown parameter from scratch. This limitation becomes critical in ab initio phase estimation, where no prior knowledge of the parameter is available. Therefore, in this regime, a measurement strategy based on fixed, pre-determined observables can not achieve optimal precision over the full parameter range without feedback. An approach to overcome this limitation is to implement adaptive strategies that permit maintaining consistent precision across the entire phase range accessible with the probe state, independently of the specific parameter values^30–35^. Previous implementations of adaptive phase estimation with squeezed states have all relied on prior knowledge of the amount of squeezing in the probe^26,36^, pre-calibrating the squeezing level, needed to optimize the measurement settings, limiting their applicability in practice. Moreover, they were also confined to half of the system’s periodicity.

The necessity of pre-calibration is not merely a laboratory inconvenience but a fundamental bottleneck for the deployment of quantum sensors in complex, non-stationary environments. In many cases, not only is the sample-induced phase shift unknown, but properties of the probe state itself, such as the squeezing strength, may also be subject to variations during the measurement time^37–39^. This is particularly evident in fields such as quantum-enhanced biosensing and plasmonics, ranging from the study of protein adsorption kinetics^40,41^, to the monitoring of bacterial growth with squeezed light^42^. In these settings, the interaction between the probe and the biological sample manifests as a change in transmitted or reflected intensity, which intrinsically modifies the detection efficiency and noise statistics in real-time. Similarly, in broadband spectroscopy^43,44^ and nonlinear microscopy^45^, factors such as varying mode overlap, dynamic loss budgeting, and phase-lock stability fluctuate between experimental runs, translating into drifting nuisance parameters that invalidate static noise models. Conventional protocols typically address these instabilities through frequent recalibration cycles, which consume measurement time and fail to track rapid environmental drifts. Optimal operation in these regimes, therefore, requires a strategy capable of tracking a time-dependent signal while simultaneously inferring the nuisance parameters that define the inference model itself. Addressing such situations requires a multiparameter estimation framework^46,47^, in which both phase and probe parameters are inferred simultaneously. Since the corresponding observables are generally non-commuting, quantum incompatibility imposes trade-offs on precision^47–50^, making it essential to investigate experimentally how adaptive protocols perform in this regime^51^. To this end, a stepwise estimation strategy has recently been introduced^52–54^, alongside several other methods aimed at boosting precision in this framework^55–58^.

In this work, we present a significant advancement in measuring unknown phase shifts with precision that surpasses the classical limit without requiring pre-calibration of the probe key parameters. By combining Homodyne Detection (HD) with Bayesian inference in an adaptive measurement procedure, we realize and experimentally validate an ab initio multiparameter estimation scheme that simultaneously learns the unknown phase and relevant probe parameters. This calibration-free strategy demonstrates genuine quantum enhancement under realistic conditions, highlighting its technological relevance for robust, deployable phase sensing. The implemented protocol dynamically adjusts the local oscillator (LO) phase in response to prior measurement outcomes, effectively steering the measurement basis toward the quadrature with the highest sensitivity at each step. Our approach achieves unconditional quantum-enhanced sensitivities for optical phase values across the entire periodicity range, without relying on systematic calibration of the probe. This is achieved through a multistep estimation procedure designed to overcome symmetrical constraints that previously confined investigations to the [0, *π*/2] range. As a result, we extend phase estimation to the entire [0, *π*) domain while directly inferring both the phase and the squeezing strength from the data. This article reports the experimental realization of quantum-enhanced optical phase estimation over the full *π*-range^59^, marking a decisive step toward practical multiparameter estimation with squeezed light. Importantly, beyond interferometric phase sensing, our framework maps directly onto a broad class of practical quantum-enhanced sensors that rely on bright optical probes and intensity readout, where intensity fluctuations provide an effective measurement of the amplitude quadrature, so the achievable sensitivity is jointly determined by drifting probe and channel parameters such as losses. Our multiparameter strategy, therefore, treats phase, squeezing, and efficiency as co-estimated parameters, enabling self-calibrated quantum enhancement under realistic drift.

## Results

### Theoretical framework

To benchmark the performance of our protocol, we refer to the parameter estimation framework^60^. In the single-parameter scenario, the aim is to measure an unknown parameter *y* by preparing a set of *M* probe states, measuring them according to a specific set of operators *Π*_*k*_, and then processing the data to retrieve information on the parameter. These choices determine the effective precision in the estimation process. For a chosen probe state and measurement strategy, the Fisher Information (FI) quantifies the information *F*[*y*] about the unknown parameter *y* contained in the measurement outcomes, optimized on all possible data processing strategies. This provides a first bound on the variance ${{{\rm{Var}}}}_{y}[\widehat{y}]$ for estimator $\widehat{y}$ of the unknown parameter, known as the Cramér-Rao bound (CRB)^61,62^. This bound sets the achievable precision for a fixed probe state and measurement scheme. Note that the value of *F*[*y*] can explicitly depend on the true value of the parameter. Optimizing the measurement strategy over all positive operator-valued measures (POVMs) leads to the quantum Cramér-Rao bound (QCRB)^63–65^, which sets the ultimate precision associated with the specific probe state, and which is determined by the quantum Fisher information (QFI) *F*_*Q*_. Overall, the variance of any unbiased estimator satisfies the following chain of inequalities:

1 $${{{\rm{Var}}}}_{y}[\hat{y}]{\ge }^{{{\rm{CRB}}}}\frac{1}{MF[y]}{\ge }^{{{\rm{QCRB}}}}\frac{1}{M{F}_{Q}},$$

where *M* represents the number of probe states used in the estimation. In our phase estimation scenario (*y* ≡ *ϕ*), it has been shown^26,66,67^ that for Gaussian probes, the optimal sensitivity to optical phase shifts corresponds to ${F}_{Q}^{{{\rm{sq}}}}=2{\sinh }^{2}(2r)$, where *r* is the squeezing parameter. Notably, for a sufficient level of squeezing, this surpasses the QFI of coherent states given by ${F}_{Q}^{{{\rm{coh}}}}=4| \alpha {| }^{2}$ when considering an equivalent number of photons in the probe, since ∣*α*∣^2^ and ${\sinh }^{2}(r)$ are the average photon number in the probe for each scheme. This limit can be saturated using HD combined with either maximum-likelihood or Bayesian estimation^67^.

The parameter dependence of the FI highlights the need for adaptive measurement strategies, which dynamically adjust the detection basis to maintain optimal sensitivity across the entire phase range. In fact, fixed quadrature measurements provide sub-SNL precision only in restricted phase intervals. This naturally leads to using the FI as the fundamental figure of merit, where an adaptive feedback loop can be designed to steer the measurement toward the point of maximal FI, ensuring that the protocol operates close to the CRB across the entire accessible phase range.

In actual experimental implementations, it is also necessary to take into account that the effective probe state may also vary during the measurement process. Different phase shifts *ϕ* are associated with different fluctuations in the probe itself, such as variations in the amount of squeezing, thus affecting the attainable precision. As a result, the FI must be regarded as a function of both the unknown phase and the probe parameter $F[\vec{y}]$, where $\vec{y}=(\phi,r)$. Optimal strategies for phase estimation, therefore, can not be devised without simultaneously accounting for probe fluctuations, since relying on an incorrect pre-calibrated squeezing value can introduce systematic biases in the final estimate^37^. This naturally motivates the adoption of a multiparameter estimation framework, where the resources are invested in both phase and probe parameter estimation. In this multiparameter framework, the uncertainty in the simultaneous estimation provided by the estimators $\widehat{\vec{y}}=(\widehat{\phi },\widehat{r})$ is described by the covariance matrix ${{{\boldsymbol{\Sigma }}}}_{\vec{y}}[\widehat{\vec{y}}]$, which is constrained by the multiparameter version of the (Q)CRB^46^:

2 $${{{\boldsymbol{\Sigma }}}}_{\vec{y}}[\hat{\vec{y}}]{\succcurlyeq }^{{{\rm{CRB}}}}\frac{1}{M}{{{\boldsymbol{F}}}}^{-1}[\vec{y}]{\succcurlyeq }^{{{\rm{QCRB}}}}\frac{1}{M}{{{\boldsymbol{F}}}}_{{{\boldsymbol{Q}}}}^{-1}.$$

Here, ${{\boldsymbol{F}}}[\vec{y}]$ and ***F***_***Q***_ denote the FI and QFI matrices, respectively, and the notation ***A*** ≽ ***B***, indicates that ***A*** − ***B*** is positive semidefinite. In this context, one must consider the fact that there is no guarantee that a single measurement strategy is optimal for estimating multiple parameters, meaning that, in general, it is not ensured that the estimates can attain their QCRBs simultaneously^68^. Furthermore, in the multiparameter scenario, ${{\boldsymbol{F}}}[\vec{y}]$ can present singularities for some specific parameter values^69^. This is the case of joint estimation of the phase and squeezing parameter with a squeezed vacuum state, reflecting the fact that the measurement outcomes do not provide independent information about both parameters. From a practical perspective, these aspects make the design of adaptive protocols more demanding.

### Experimental platform and adaptive protocol

**Fig. 1:**
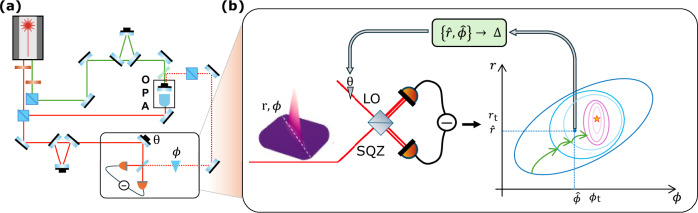
Sketch of the experimental setup and of the implemented protocol. **a** Squeezed-vacuum probes are generated by pumping an optical parametric amplifier (OPA) with a single-mode continuous-wave laser at 532 nm. To achieve high levels of squeezing, the pump and LO beams are both spatially filtered in identical mode-cleaner cavities to maximize the overlap between the squeezed field and the LO at the homodyne BS. The relative phase between the squeezed state and the LO is stabilized with a phase-locked loop (PLL). The LO phase *θ* is finely tuned by means of a piezoelectric stage, while an FPGA-based feedback system allows dynamic selection and locking of the measurement quadrature. The phase *ϕ* can be changed with a liquid crystal device in the path of the squeezed beam. **b** The probe is a squeezed state, represented by its Wigner-function ellipse (purple outline). The unknown phase shift (denoted as the white dashed line) is imprinted on the probe by the liquid-crystal element, which rotates the squeezing ellipse in phase space. At each step of the protocol, the homodyne data collected are used to reconstruct the multiparameter posterior distribution. As homodyne outcomes are acquired and processed, the joint posterior becomes progressively more concentrated (represented in the sketch as the contour ellipses). Blue contours indicate the posterior after the initial raw estimation steps, while pink contours show the refined posterior during the final adaptive stage. From this posterior, the estimate is updated, and the optimal measurement angle *θ* is computed. A feedback *Δ*, derived from the current estimates, is then applied to adjust the LO phase for the next measurement. Repeating this cycle progressively refines the posterior until, at the end of the protocol, the estimates converge to the true values of phase *ϕ*_*t*_ and of the squeezing parameter *r*_*t*_.

The squeezed vacuum state is generated at a wavelength of 1064 nm through the cavity-enhanced interaction of a pump beam at 532 nm with a periodically-poled nonlinear crystal inside an optical parametric amplifier (OPA). The phase of the generated squeezed vacuum state is kept fixed in time using an FPGA-based locking scheme, and, via a liquid crystal device, we set the arbitrary phase *ϕ*, which corresponds to the parameter of interest to be estimated. For this purpose, we perform homodyne measurements along an arbitrary quadrature angle, determined by the phase *θ* of the LO that can be controlled with a piezoelectric stage. The main components of the experimental setup are illustrated in Fig. 1. More details about the apparatus are reported in the SI.

For each unknown phase value *ϕ*, we employ a Bayesian estimation strategy^70^ to process the experimental data. Specifically, a sequence of *M* homodyne measurements, ***x*** = {*x*_1_, *x*_2_, . . . , *x*_*M*_}, is collected and used to update our knowledge of the parameter through Bayes’ theorem^67^. Assuming no specific prior knowledge regarding both the parameter of interest *ϕ* and the squeezing level of the employed probe, i.e., adopting a flat prior distribution within the interval *ϕ* ∈ [0, *π*) and *r* ∈ [0, 3]. The posterior probability reconstructed after a large number *M* ≫ 1 of measurements can be expressed as:

3 $$p(\varphi,r| {{\boldsymbol{x}}})=\frac{1}{{{\mathcal{N}}}}{\prod }_{x\in {{\boldsymbol{x}}}}p{(X=x| \varphi,r)}^{Mp(X=x| \varphi,r)}.$$

Here, *p*(***X***∣*φ*, *r*) represents the homodyne probability distribution, which consists of a Gaussian distribution centered in zero, and with variance:

4 $${\sigma }^{2}(\varphi,r,\eta )=\frac{1}{4}\left(\eta {e}^{-2r}{\cos }^{2}\varphi+(1-\eta ){\cos }^{2}\varphi+{e}^{2r}{\sin }^{2}\varphi \right),$$

with *φ* = *ϕ* − *θ* and *η* accounting for losses in the setup.

Once having reconstructed the posterior probability, we derive the estimates $\widehat{\phi }$ and $\widehat{r}$ of both parameters by calculating the mean value of the posterior distribution. To carry out ab-initio sub-SNL estimation of the phase *ϕ* over the full range [0, *π*), we implement an online adaptive protocol consisting of two main stages. In the first step, we collect a small fraction of data *M*_*R*_ for different settings of the LO phase *θ*. In this way, we can remove the intrinsic ambiguity *π* − *ϕ* of squeezed-state interferometry, which constrained previous experimental phase estimation protocols with homodyne measurements^26^ to *ϕ* ∈ [0, *π*/2]. From these outcomes, a Bayesian update is performed to reconstruct the posterior distribution, from which rough estimates of both the phase and the squeezing parameter can be extracted. As the numerical evaluation of the posterior distribution can be computationally demanding, we approximate it by employing the Sequential Monte Carlo (SMC) technique^71,72^ that allows us to determine the online feedback. It consists of discretizing the posterior distribution into *n*_p_ particles ${\{{\phi }_{k}\}}_{k=1}^{{n}_{p}}$ and ${\{{r}_{k}\}}_{k=1}^{{n}_{p}}$, associated with weights ${\{{\omega }_{k}({{\boldsymbol{x}}},\theta )\}}_{k=1}^{{n}_{p}}$. The difference between the single- and multiparameter protocols lies in associating the index *k* to either a discretized phase *ϕ*_*k*_ or a point (*ϕ*_*k*_, *r*_*k*_) in the 2-parameter space. The weights satisfy ∑_*k*_*w*_*k*_ = 1 and are distributed according to the prior information. These are sequentially updated with the observed homodyne measurements *x* ∈ ***x***, in such a way that the final mean and variance of the posterior can be efficiently computed with the following discrete sums:

5 $$\widehat{\phi } \;=\sum _{k=1}^{{n}_{{{\rm{p}}}}}{\omega }_{k}({{\boldsymbol{x}}},\theta ){\phi }_{k},$$

6 $$\widehat{r} \;=\sum _{k=1}^{{n}_{{{\rm{p}}}}}{\omega }_{k}({{\boldsymbol{x}}},\theta ){r}_{k},$$

7 $${{{\rm{Var}}}}_{\phi }[\widehat{\phi }]\;=\sum _{k=1}^{{n}_{{{\rm{p}}}}}{\omega }_{k}({{\boldsymbol{x}}},\theta ){(\widehat{\phi }-{\phi }_{k})}^{2}.$$

Further details on the SMC techniques are reported in the SI. These preliminary estimates are then used to set the adaptive feedback by changing the LO phase at the most informative measurement point.

### Experimental results

We start by benchmarking the protocol in the single-parameter estimation scenario, where the goal is to estimate only the phase *ϕ*. In this framework, the first step of the adaptive procedure consists of acquiring data at two fixed values of the LO phase, i.e., *θ* = {0, *π*/4}. This first batch of measurements provides a first rough estimate of the parameter, which in turn allows us to identify the optimal measurement projection and thus set the phase of the adaptive protocol where the FI is maximum, thereby optimizing the next measurement projections. For a pre-calibrated squeezing level *r* and a transmission coefficient *η*, we can retrieve the effective squeezing parameter that captures the combined impact of squeezing and loss given by:

8 $${r}_{{{\rm{eff}}}}=\frac{1}{2}\log \left[\frac{{\sigma }_{{{\rm{asqz}}}}^{2}(r,\eta )}{\sqrt{{\sigma }_{{{\rm{asqz}}}}^{2}(r,\eta ){\sigma }_{{{\rm{sqz}}}}^{2}(r,\eta )}}\right].$$

The optimal measurement configuration corresponds to ${\phi }_{{{\rm{opt}}}}=\frac{1}{2}\arccos (\tanh (2{r}_{{{\rm{eff}}}}))$. In practice, the measurement basis is aligned by choosing $\theta=\widehat{\phi }-{\phi }_{{{\rm{opt}}}}$, where $\widehat{\phi }$ is the current Bayesian estimate. After this step, a new block of quadratures is collected and used to update the posterior distribution, thereby updating the parameter estimates and yielding a more accurate determination of the optimal LO phase. This adaptive cycle is repeated three times, using a total the remaining *M* − *M*_*R*_ measurements. At each iteration, the increasing amount of data yields sharper posteriors and progressively improves the determination of the adaptive measurement setting.

**Fig. 2:**
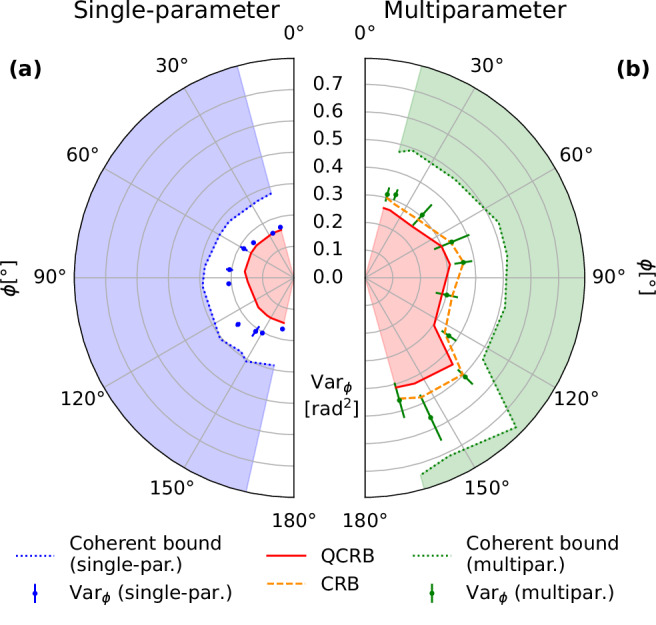
Single- and multiparameter variances of the phase estimation. We report the variances of the adaptive phase estimation (radial axis) as a function of the phase *ϕ* (angular axis), spanning the entire range [0, *π*). The experimental results ${{{\rm{Var}}}}_{\phi }[\widehat{\phi }]$ obtained in the single- [panel (**a**)] and multiparameter [panel (**b**)] adaptive protocols are reported as green and blue dots, respectively. The corresponding error bars represent the standard deviations over 5 (10) repetitions of the experiment, in the single-parameter (multiparameter) case. In both panels, we also report the corresponding classical bounds, i.e., the QCRB for a coherent probe with the same average number of photons, as a blue line for the former and a green line for the latter case, while the QCRB is represented by a red line in both cases. For the multiparameter protocol, we also report the classical CRB (orange dashed line). The experimental single-parameter protocol employs a total of *M* = 5000 homodyne measurements, among which *M*_R_ = 200 are used to obtain the rough estimate. Instead, in the multiparameter estimation, the total number of measurements amounts to *M* = 20,000, while the rough estimation needs *M*_R_ = 1200 of them.

The experimental precision is obtained as the variance of the reconstructed posterior distribution for different values of *ϕ*, after the collection of *M* homodyne quadratures. The obtained results for the phase estimation in the entire range *ϕ* ∈ [0, *π*) are reported in Fig. 2a. The experimental measurements are compared with the QCRB computed for squeezed-vacuum probes: ${{{\rm{Var}}}}_{\phi }^{{{\rm{(sqz)}}}}[\widehat{\phi }]=1/{F}_{Q}^{{{\rm{sq}}},{{\rm{eff}}}}$, in red, representing the ultimate sensitivity attainable with Gaussian resources for the given squeezing level and loss in such conditions, with ${F}_{Q}^{{{\rm{sq}}},{{\rm{eff}}}}=2{\sinh }^{2}(2{r}_{{{\rm{eff}}}})$. In the single-parameter configuration, when the LO phase is set to the optimal working point, homodyne detection is an optimal measurement for squeezed Gaussian probes; consequently, in the lossless scenario, its FI equals the QFI and the corresponding homodyne CRB overlaps with the QCRB^26,67^. The achieved variances are compared with the optimal precision attainable with classical resources of equal mean photon number, quantified by the corresponding QCRB for coherent states: ${{{\rm{Var}}}}_{\phi }^{{{\rm{(coh)}}}}[\widehat{\phi }]=1/(4\langle n\rangle )=1/[4{\sinh }^{2}(r)]$ reported as a blue line in the figure. Importantly, to assess the unconditional advantage over classical light, the coherent bound is not loss-corrected but is evaluated at the probe’s actual mean photon number linked to the squeezing parameter *r*. The bound is evaluated across all inspected phase values, each time accounting for the corresponding pre-calibrated levels of squeezing. These pre-calibrations reveal that the parameters are not fixed but instead fluctuate due to experimental instabilities, such as variations in the source during different data acquisitions and imperfections in the alignment procedures.

The experimental variances lie consistently below the classical bound, demonstrating unambiguous sub-SNL sensitivity. Moreover, the measured precision closely approaches the squeezed-state QCRB, confirming that our adaptive Bayesian strategy operates near the optimal point across the entire phase domain. While this strategy enables the saturation of the ultimate precision bound for the phase estimation with homodyne measurements, it also requires a precise calibration of the setup efficiency *η* and of the squeezing level *r*, before performing the actual experiment. For this calibration to be effective, it must be sufficiently precise, hence, employing a significant amount of resources, which are not accounted for in the overall resource budget.

Importantly, our experimental results show that in realistic scenarios, both the level of squeezing and losses are not fixed, but change depending on the specific phase value under investigation. This can arise because the liquid crystal may exhibit different transmission at different phases, or because the squeezing parameter drifts over time due to temporal instabilities of the source. Consequently, different phase values refer to different values of the bound on the attainable precision as emerges in Fig. 2a. This observation further motivates the need of a multiparameter strategy, in which both the phase and the squeezing parameter are inferred simultaneously from the same dataset. Such an approach enables the protocol to adapt in real-time to fluctuations of the probe, eliminating the dependence on previous calibration, which can jeopardize the final estimate, while still achieving sub-SNL estimation of the phase, as described below. We stress that within each estimation run, with the employed estimation model, we do not aim to track parameter variations dynamically; indeed, on the timescale of a few seconds, the phase-locking loop and the source stability are sufficient to maintain approximately constant values of *ϕ* and *r*.

In the multiparameter regime, for a fixed homodyne setting, the classical FI matrix becomes singular, which in this case showcases the impossibility of estimating both parameters simultaneously in the absence of prior information. The adaptive strategy, by measuring in different homodyne settings, restores a non-singular FI matrix, thus circumventing such a limitation. However, the measurement settings that maximize the phase sensitivity coincide with the sensitivity minimum for the squeezing parameter, and vice versa. This trade-off reflects the incompatibility of the relevant observables and precludes simultaneous attainment of the ultimate precision bounds for both parameters (see SI for additional details).

Since our primary goal remains to estimate *ϕ* with the highest possible precision, thus prioritizing the estimate of *ϕ* over *r*, it might be expected that selecting the single-parameter phase-optimal setting would suffice. In practice, however, an excessively imprecise estimate of *r* degrades the adaptive LO feedback and therefore the phase estimate (see simulation results in SI). To balance these competing requirements, we implement an LO feedback rule that aims to cancel the correlation between the two parameters’ estimation errors. The optimal strategy corresponds to setting the quadrature where phase and squeezing estimates are effectively decorrelated, i.e., where the off-diagonal elements of the FI matrix *F*_*ϕr*_ vanish. This corresponds to setting $\theta=\widehat{\phi }-\arccos {e}^{2 \widehat{r}} / \sqrt{{e}^{4 \widehat{r}}+\eta }$, with *η* the detection efficiency. Both the estimates $\widehat{r}$ and $\widehat{\phi }$, needed to compute the feedback updates, are retrieved as mean values of the two-dimensional posterior distribution, which is efficiently reconstructed with the SMC approach, further justifying its adoption to significantly speed up the numerical reconstruction, especially in this multiparameter framework. The precisions achieved on the phase estimates for the multiparameter protocol are reported in Fig. 2b, showing also in this case the ability to obtain quantum-enhanced performance, while ensuring robustness to a varying squeezing level. Overall, we observe gains of 1.79 ± 0.40 dB below the SNL, not corrected for loss, and averaged over 10 different phase values, with the conventional single-parameter (pre-calibrated) approach and 1.78 ± 0.33 dB below the SNL with our ab-initio multiparameter strategy concerning the phase estimate. These results highlight both the robustness and the unconditional character of the protocol, since the quantum advantage is maintained without prior calibration of the probe and for all the values of *ϕ*, overcoming also the restrictions of previous approaches limited to narrower phase intervals.

**Fig. 3:**
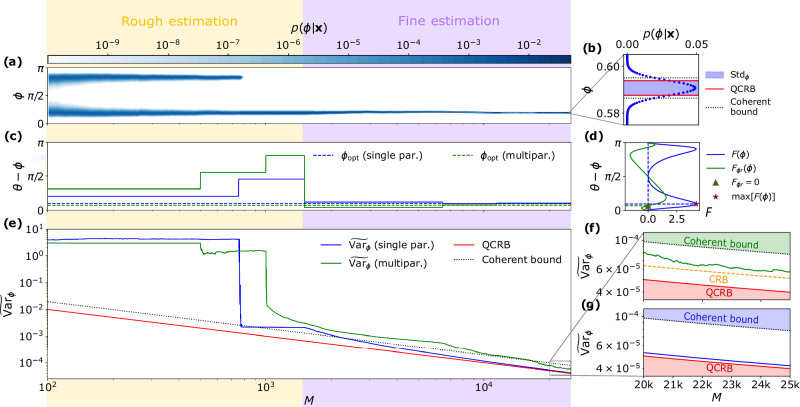
Adaptive phase estimation single- and multi-parameter protocols. In panel **a**, we report the evolution of the posterior distribution *p*(*ϕ*∣***x***) as a function of the number of homodyne data measured along the adaptive single-parameter protocol in the range *M* ∈ [100, 25,000]. During the rough estimation, highlighted by a yellow background, we observe that measuring different quadrature angles (*θ* = {0, *π*/4} and *θ* = {0, *π*/4, *π*/2} in the single- and multiparameter estimations, respectively) allows us to disambiguate the estimate between the ranges *ϕ* ∈ [0, *π*/2] and *ϕ* ∈ [*π*/2, *π*). At the end of this stage, the current estimation $\widehat{\phi }$ is employed to shift the LO phase to its optimal value $\theta=\widehat{\phi }-{\phi }_{{{\rm{opt}}}}$. The remaining measurements (purple background) are taken within the optimal homodyne configuration, which is further updated two times before reaching the final estimation, extrapolated from the posterior distribution reported in panel (**b**). This inset shows how the precision of the phase estimation (blue shaded area) tightly approaches the squeezed QCRB (red line), while surpassing the classical bound (black dotted line). The changes of the LO angle, along the adaptive protocol, are illustrated in panel (**c**). It shows that the quantity *θ* − *ϕ* is close to its optimal value, depending on the chosen protocol. In the single parameter estimation (in blue), it coincides with the value corresponding to the maximum of the FI [in panel (**d**)], while in the multiparameter approach (green), we aim at canceling the off-diagonal element of the FI matrix *F*_*ϕr*_ [shown in panel (**d**)]. In panel **e**, the normalized variance ${\widetilde{{{\rm{Var}}}}}_{\phi }[\widehat{\phi }]\equiv [\widehat{\phi }]\cdot {F}_{Q}^{{{\rm{sq}}},{{\rm{eff}}}}$ is reported as a function of *M*, thus having a common ultimate precision bound (solid red line) for both the single- and multiparameter variances, illustrated as blue and green lines, respectively. This panel also shows how the disambiguation and the LO feedback positively affect the precision of both the estimation protocols, although the multiparameter one exhibits a slower convergence to values below the classical bound (black dotted line). Panels **f** and **g** provide zoomed-in views of the marked area, demonstrating convergence to the bound for the single-parameter strategy and for the multi-parameter strategy, respectively. The latter converges to the CRB computed for the two-parameter estimate that does not coincide with the QCRB.

A more direct comparison of the single and multiparameter strategies is reported in Fig. 3, where we investigate how the estimation variances scale with the number of homodyne measurements. More specifically, in Fig. 3a, the experimental single-parameter posterior distribution is reported for a representative phase estimate as a function of the number of homodyne measurements used to update the Bayesian posterior. Fig. 3b shows the reconstructed distribution at the end of the protocol, quantifying the achieved precision as ${{{\rm{Std}}}}_{\phi }[\widehat{\phi }]=\sqrt{{{{\rm{Var}}}}_{\phi }[\widehat{\phi }]}$. Figure 3c illustrates the sequence of feedback values applied at successive steps of the estimation protocol. In the single-parameter case, after the rough estimate, the feedback angles correspond to the quadrature maximizing the Fisher information, while in the multiparameter case, the rough estimation stage is split into three steps, *θ* = {0, *π*/4, *π*/2}, to improve the estimation of *r* needed to implement the adaptive feedback. The feedback angles for the fine estimate are instead determined by the condition where the off-diagonal elements of the FI matrix vanish (see Fig. 3d). The single-parameter strategy, which assumes prior knowledge of the squeezing parameter, required to set the feedback of the adaptive algorithm, displays faster convergence to the bound as emerges in Fig. 3e. As expected, because the multiparameter strategy jointly infers both the phase and the probe parameters from the same homodyne record, it requires a larger number of measurements before reaching convergence, since every measurement outcomes contribute to simultaneously estimate both *ϕ* and self-calibrate the squeezing level of the probe. Nevertheless, and most importantly, even in the absence of any prior calibration of the squeezing, the multiparameter protocol achieves phase estimation precision at the sub-SNL level once convergence is reached. This demonstrates that quantum-enhanced performance can be retained even without relying on external information about the probe. Finally, the advantage over coherent-light phase estimation is further emphasized in the zoomed-in region showing the convergence behavior. Both our single-parameter (Fig. 3f) and multiparameter (Fig. 3g) strategies consistently outperform the lossless coherent bound calculated without rescaling by the overall transmission coefficient *η*.

**Fig. 4:**
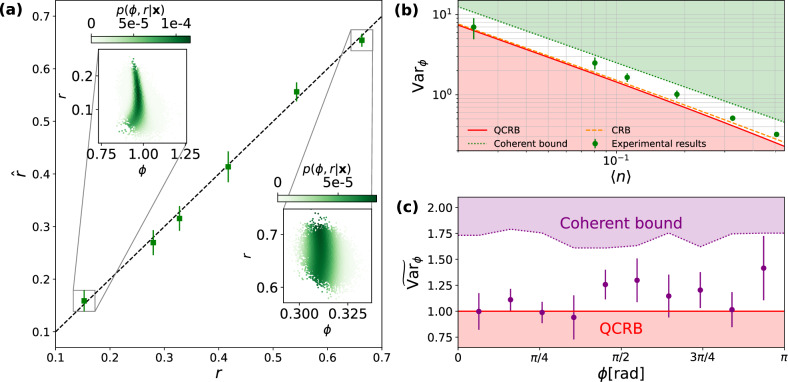
Robustness of the multiparameter adaptive estimation protocol to different squeezing strengths and a fully black-box approach. In **a**, the green squares represent the estimated squeezing level $\widehat{r}$, within the multiparameter adaptive protocol, compared to the actual values *r*. As an example, we illustrate the experimentally reconstructed posterior distributions *p*(*ϕ*, *r*∣***x***), corresponding to the lowest (top-left inset) and the highest (bottom-right inset) squeezing levels. **b** Scaling of the experimental phase variances compared to the relative bounds: the QCRB for squeezed vacuum probes (red), the CRB (orange), and the QCRB for coherent states (green). The data lie all below the coherent bounds, computed without considering losses. Panel **c**, reports the results obtained with a fully black-box approach, in which the efficiency *η* is also estimated. The normalized variances ${\widetilde{{{\rm{Var}}}}}_{\phi }[\widehat{\phi }]$ (purple dots) are compared with the classical lossless (dash-dotted purple line) bound. In all the panels, the reported errors correspond to standard deviations over 10 repetitions of the experiments.

We next demonstrate the full strength of the multiparameter protocol in a challenging regime where not only the optical phase, but also the squeezing parameter itself, undergoes significant variations, to test the robustness of this protocol to different squeezing levels. The estimated values of *r* are shown in Fig. 4a as a function of the calibrated squeezing. Remarkably, the adopted strategy can faithfully infer the true value of *r* across all operating conditions, without requiring any prior calibration or external reference. At the same time, the variance of the simultaneous phase estimate, reported for different squeezing regimes, remains consistently below the classical bound as shown in Fig. 4b, confirming the ability of our protocol to preserve quantum-enhanced performance even under strongly different conditions of the probe. This is achieved since the adaptive feedback is explicitly designed to minimize the variance of the parameter of interest, namely the phase *ϕ*. This feature is also related to the shape of the reconstructed posterior, which is narrower along the phase axis, while still providing reliable estimates of the squeezing. Our results highlight the versatility of the adopted multiparameter approach that enables the simultaneous estimation of probe and signal parameters, while allowing the feedback strategy to be tailored to prioritize the precision of the most relevant observable. Moreover, by analyzing the dependence of the estimation variance on the squeezing strength *r*, which directly quantifies the photon number in the probe, we clearly observe an improvement with respect to the classical coherent bound. As *r* increases, the variance decreases accordingly, and the gap between squeezed light estimation and the classical coherent state shot-noise benchmark widens. This behavior provides an experimental indication that stronger squeezing yields a larger improvement in estimation precision at fixed resources.

Finally, to make the protocol completely independent of any calibration, we tested its performance under the most demanding condition, in which no prior knowledge of the overall detection efficiency *η* is also assumed. Estimating losses jointly with the phase is particularly challenging in quantum multiparameter estimation since the probe and measurement choices that optimize one parameter are generally not optimal for the other, leading to fundamental tradeoffs and, in general, preventing a single measurement strategy from simultaneously saturating the corresponding single-parameter bounds^73^. Furthermore, *η* is a global setup parameter expected to be static (or slowly varying) over a run; carrying a full Bayesian representation of such a static nuisance parameter in an online update substantially increases the dimensionality and can lead to degeneracies in the numerical approximation. To overcome this limitation, we adopt a hybrid strategy that combines Bayesian inference with a maximum-likelihood (ML) estimation of *η*, which has been demonstrated to remain stable and computationally efficient^74,75^. Specifically, during each update of the three-dimensional posterior, *η* is set to the maximizer of the *η-*posterior at the previous step and used as a plug-in value in the likelihood for the Bayesian update of (*ϕ*, *r*). This update bypasses full three-parameter inference, retaining the most consistent value of *η* in every step of the estimation process without the need for additional measurement, focusing only on the physically relevant parameters *ϕ* and *r*. This mixed approach allows us to maintain quantum-enhanced performance (see Fig. 4c) without relying on any external calibration of the probe or the detection chain, thereby validating the fully unconditional nature of the protocol.

## Discussion

Our work presents the first experimental demonstration of adaptive multiparameter quantum phase estimation with squeezed vacuum states, achieving sub-SNL precision across the full periodicity interval [0, *π*) without relying on prior calibration of key system parameters such as the squeezing level and detection efficiency. These parameters are known to fluctuate in realistic scenarios, both due to temporal drifts of the source and to sample-dependent transmission, making calibration-based approaches inherently unstable and ultimately compromising the effectiveness of adaptive measurement strategies. By implementing an adaptive multiparameter protocol that simultaneously infers the phase *ϕ* and the relevant nuisance parameters, we overcome this calibration bottleneck while retaining unconditional quantum advantage over the entire unambiguous phase range. Crucially, the method is robust to consistent fluctuations of *r*, maintaining quantum-enhanced performance across a wide operating range.

These findings demonstrate the feasibility of self-calibrating, ab-initio quantum metrology protocols that are resilient to probe instabilities and experimental drifts, thereby extending the practical applicability of squeezed light for precision sensing. Beyond enabling reliable operation under realistic conditions, our results establish a pathway toward scalable implementations in advanced interferometric platforms and distributed quantum sensor networks, which rely on the simultaneous estimation of multiple parameters.

## Supplementary information

Supplementary Information

Transparent Peer Review file

## Data Availability

The data that support the findings of this study are available from the corresponding author upon request.

## Code availability

The codes for this study that support the findings are available from the corresponding authors upon request.
