# Supplementary material for: Multiparameter quantum-enhanced adaptive metrology with squeezed light
Source: Nat Commun. 2026 May 14;17:6418. doi: 10.1038/s41467-026-73069-1 (PMC13376632; doi:10.1038/s41467-026-73069-1)
Supplement: Supplementary file 1 — Supplementary Information [file 41467_2026_73069_MOESM1_ESM.pdf]

Supplementary Information for: Multiparameter quantum-enhanced adaptive metrology with squeezed light

Giorgio Minati,[1] Enrico Urbani,[1] Nicolò Spagnolo,[1] Valeria Cimini,[1,*] and Fabio Sciarrino[1]
[1]*Dipartimento di Fisica, Sapienza Università di Roma, Piazzale Aldo Moro 5, I-00185 Roma, Italy*

CONTENTS

Supplementary Note 1. Experimental Setup Characterization 1

Supplementary Note 2. Squeezed light characterization and Phase Locking stability 2

Supplementary Note 3. Additional Experimental Data and Pre-calibration measurements 4

Supplementary Note 4. Bounds on simultaneous estimation of phase and squeezing level 7

Supplementary Note 5. Overcoming Fisher Information Matrix Singularity 11

Supplementary Note 6. Sequential Monte Carlo 13

References 16

SUPPLEMENTARY NOTE 1. EXPERIMENTAL SETUP CHARACTERIZATION

In what follows, we present a complete description of the setup employed to perform the experiment, which is schematically illustrated in Supplementary Fig.1.

The main laser source is a Coherent Prometheus Nd:YAG double-wavelength continuous-wave laser emitting 1.77 W at 1064 nm and 104 mW at 532 nm. The 532 output serves as the pump for the squeezed state generation. Before this step, the action of a Half-Wave Plate (HWP) and a Polarizing Beam Splitter (PBS) allows us to tune the total power of the 532 nm beam. Phase modulation in the MHz frequency range is applied using an electo-optical modulator (EOM) to produce sidebands used for the Pound-Drever-Hall (PDH) locking. All the beams employed are cleaned through three mirrors Fabry-Perot ring cavities, each one used as an optical resonator for mode-cleaning (MC), filtering out spatial modes different from the TEM_{00}.

Generation stage. The squeezed light source is the commercial source developed by Noisy Labs. It consists of an optical parametric amplifier based on a type-0 periodically poled KTP (PPKTP) crystal configured as a hemilithic standing-wave cavity pumped via the mode-cleaned 532 nm beam. One mirror is piezo-mounted for length control, while the high-reflection coating on one end face of the PPKTP acts as the second mirror at 1064 nm (and appropriately coated for 532 nm). The opposite crystal face is anti-reflection coated. The cavity is locked on resonance via PDH. The crystal temperature is actively stabilized to maintain quasi-phase matching at the pump wavelength and to suppress slow thermal drifts; length control is handled by the piezo and fine-tuned with an additional temperature control. The output of the source can be extracted using a Dicroic Mirror (DM) that reflects only 1064 nm wavelength. The 1064 nm beam is split into two paths, each subjected to the same mode-cleaning procedure used for the pump. This ensures high spatial purity and excellent overlap at the detection stage between the squeezed field and the local oscillator (LO), which is essential for resolving large levels of squeezing. One path forms the LO for balanced homodyne detection, while the second serves as a coherent control beam used to stabilize the phase of the squeezed field.

Measurement stage. We employ a balanced homodyne detection scheme where the LO and the squeezed beam are mixed on a 50:50 beam splitter (BS) and the photocurrent difference is measured via an oscilloscope. The detected quadrature is set by the LO phase θ controlled via a motorized piezoelectric stage.

Phase locking loop. The phase of the squeezed beam is stabilized using a coherent control beam that is spatially mode-matched to the squeezed mode. The control beam is phase-modulated by an EOM at radio frequencies and injected in the squeezer cavity. When in resonance, the bright leakage provides an alignment reference for the squeezed path; at low power it generates a phase-sensitive error signal upon interference with the LO. This signal is demodulated and used in a phase-locked loop (PLL).

* valeria.cimini@uniroma1.it

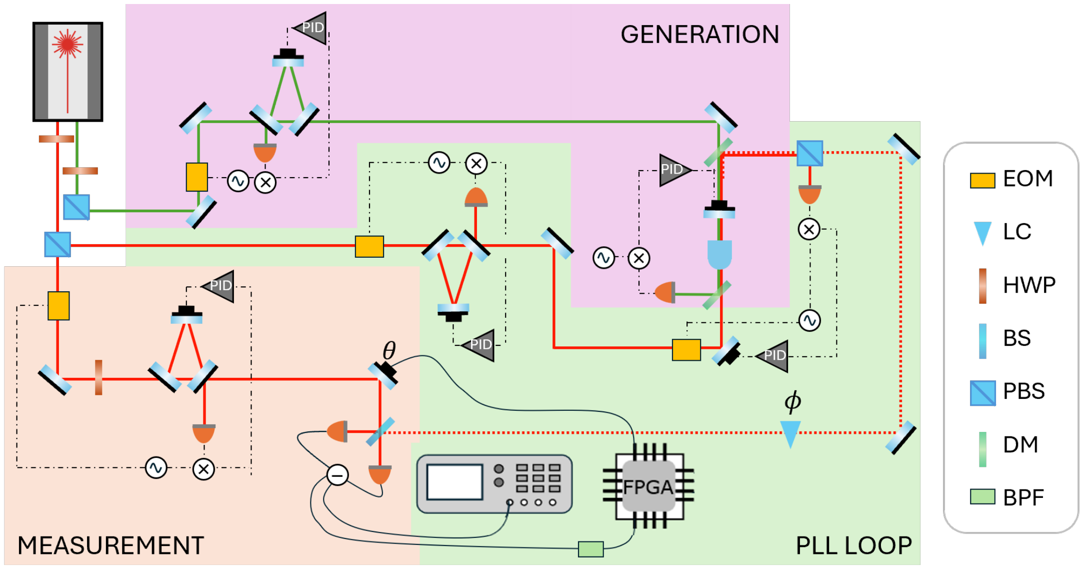

Supplementary Figure 1. **Experimental setup.** The laser provides two outputs. The 532 nm beam pumps the squeezer cavity, while the 1064 nm beam is split by a half-wave plate (HWP) and a polarizing beam splitter (PBS) into a control beam and a local oscillator (LO), both mode-matched to the squeezed output. The setup comprises three main blocks: (i) squeezed-state generation, (ii) homodyne measurement, and (iii) phase stabilization using the control beam in a phase-locked loop (PLL). The squeezer cavity is stabilized via a Pound–Drever–Hall (PDH) scheme employing an electro-optic modulator (EOM), a piezoelectric actuator, and a photodiode; the same infrastructure is used to keep three mode-cleaner cavities resonant with their respective beams. After the squeezer, the 532 nm pump and the 1064 nm control/squeezed beams are separated with dichroic mirrors (DM). The phase ϕ along the squeezed-beam path is tuned with a liquid-crystal (LC) phase shifter and measured by balanced homodyne detection (HD), where the LO and the squeezed beam interfere on a 50:50 beam splitter (BS). The quadrature angle θ is swept using a piezoelectric stage driven by an FPGA, which reads the DC output of the homodyne detector after a band pass filter (BPF) to suppress electronic noise.

In this configuration, the OPA produces squeezed vacuum at 1064 nm the MHz detection band. The combination of mode cleaning, PDH-stabilized cavities, and coherent-control locking provides the spatial mode quality and phase stability $\theta - \phi$ required to resolve sub-shot-noise quadrature fluctuations and to implement the adaptive, real-time estimation protocol.

SUPPLEMENTARY NOTE 2. SQUEEZED LIGHT CHARACTERIZATION AND PHASE LOCKING STABILITY

In order to characterize the generated squeezed state, we measure the photocurrent noise modulation in different configurations, which we report in Supplementary Fig.2, where all the traces are zero-span measurements at 6 MHz. We take the shot-noise as a reference, hence, setting the noise level which is measured when only the LO is sent to the homodyne detector at 0 dB. A 1 Hz triangular-wave-shaped scan of the measured quadrature angle allows us to estimate the maximum and minimum noise modulation (blue trace in Supplementary Fig.2), thereby quantifying the squeezing and anti-squeezing level as $\sigma^2_{\rm sqz} = -6.12 \pm 0.28$ dB and $\sigma^2_{\rm asqz} = 11.35 \pm 0.28$ dB. Additionally, within the same settings, we measure the electronic dark-noise when no light at all is sent to the detector, obtaining a noise attenuation of $\sigma^2_{\rm dark} = -14.9 \pm 1.0$ dB

In what follows, we describe the implementation of the FPGA-based locking procedure employed to measure the squeezed field quadrature along an arbitrary angle. First, we model the fields incoming at the homodyne detectors as:

$$E_1 = E_{\rm LO} e^{i(\omega t + \vartheta)}, \tag{1}$$

$$E_2 = E_{\rm CF} e^{i(\omega t + \beta \sin(\Omega t))}. \tag{2}$$

The first is the LO field, oscillating with an amplitude $E_{\rm LO}$ at the optical frequency ω, with a phase ϑ set by the LO piezo mirror. The second field is the weak coherent field superimposed onto the squeezed light path, whose oscillation has amplitude $E_{\rm CF}$ and, in addition to the optical frequency ω, is phase-modulated with sidebands of frequency Ω and modulation depth β. The interference of these in the HD detection BS produces the output fields E_3 and E_4, which are computed as follows:

$$\begin{pmatrix} E_3 \\ E_4 \end{pmatrix} = \frac{1}{\sqrt{2}} \begin{pmatrix} 1 & i \\ i & 1 \end{pmatrix} \begin{pmatrix} E_1 \\ E_2 \end{pmatrix} = \frac{1}{\sqrt{2}} \begin{pmatrix} E_1 + iE_2 \\ iE_1 + E_2 \end{pmatrix}. \tag{3}$$

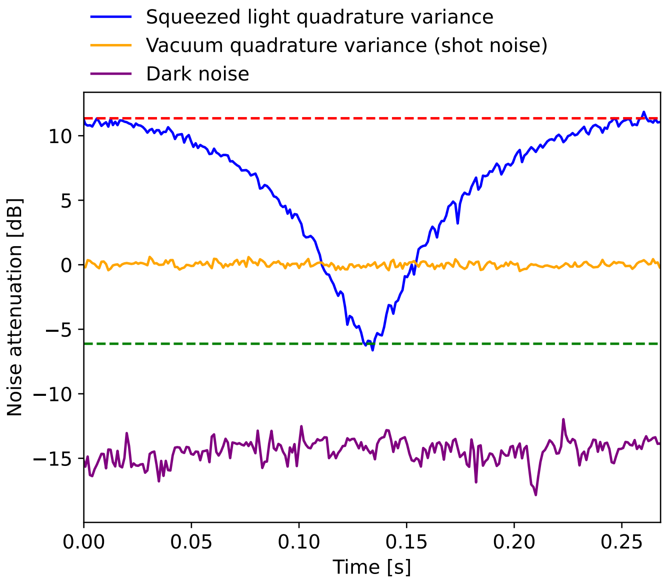

Supplementary Figure 2. **Squeezed quadratures noise attenuation, compared to the shot noise and the dark noise.** In this figure, we report the squeezed light variance attenuation (blue trace), in units of shot-noise. In particular, it has been acquired by scanning the LO phase by ramping the piezo mirror with a triangular wave with a frequency of 1 Hz. Additionally, we also report the shot noise (yellow trace) and the electronic dark noise (purple trace). All three traces have been acquired with a zero-span measurement at 6 MHz.

The corresponding photocurrents measured at the HD detector are proportional to the output fields intensities P_3 and P_4:

$$P_3 = |E_3|^2 = \frac{1}{2}(|E_1|^2 + |E_2|^2 + iE_1^*E_1 - E_1E_1^*), \tag{4}$$

$$P_4 = |E_4|^2 = \frac{1}{2}(|E_1|^2 + |E_2|^2 - iE_1^*E_1 + E_1E_1^*), \tag{5}$$

therefore, the photocurrents subtraction reads:

$$P_{\mathrm{HD}} = P_3 - P_4 = i(E_1^*E_2 - E_1E_2^*) = \tag{6}$$

$$= iE_{\mathrm{LO}}E_{\mathrm{CF}}\left(e^{i(-\vartheta+\beta\sin{(\Omega t)})} - e^{-i(-\vartheta+\beta\sin{(\Omega t)})}\right) = \tag{7}$$

$$\simeq iE_{\mathrm{LO}}E_{\mathrm{CF}}\left(e^{-i\vartheta}\left[J_0(\beta) + 2iJ_1(\beta)\sin{(\Omega t)}\right] - e^{i\vartheta}\left[J_0(\beta) - 2iJ_1(\beta)\sin{(\Omega t)}\right]\right) = \tag{8}$$

$$= 2E_{\mathrm{LO}}E_{\mathrm{CF}}\left(J_0(\beta)\sin{(\vartheta)} - 2J_1(\beta)\sin{(\Omega t)}\cos{(\vartheta)}\right), \tag{9}$$

where, in (8), the terms $e^{\pm i\beta\sin{(\Omega t)}}$ have been expressed as a Bessel series truncated at order 1. The part of Eq.(9) oscillating at 2Ω, selected using a high-pass filter, is then mixed with an electronic local oscillator $E_3 = E_{\mathrm{eLO}}\sin{(\Omega t + \varphi_{\mathrm{demod}})}$, obtaining;

$$P_{\mathrm{HD}}^{\mathrm{mix}} = -4E_{\mathrm{LO}}E_{\mathrm{CF}}J_1(\beta)\sin{(\Omega t)}\cos{(\vartheta)} \,\cdot\, E_{\mathrm{eLO}}\sin{(\Omega t + \varphi_{\mathrm{demod}})} = \tag{10}$$

$$= -4E_{\mathrm{LO}}E_{\mathrm{CF}}E_{\mathrm{eLO}}J_1(\beta)\cos{(\vartheta)}\left(\sin{(2\Omega t + \varphi_{\mathrm{demod}})} + \sin{(\varphi_{\mathrm{demod}})}\right). \tag{11}$$

A low-pass filter eliminates the terms oscillating at 2Ω, resulting in the following error signal:

$$P_{\mathrm{cos}}^{\mathrm{err}} = -4E_{\mathrm{LO}}E_{\mathrm{CF}}E_{\mathrm{eLO}}J_1(\beta)\cos{(\vartheta)}\sin{(\varphi_{\mathrm{demod}})}. \tag{12}$$

Therefore, we have access to the first component of (9) (which we will call $P_{\mathrm{sin}}^{\mathrm{err}}$ in what follows) and (12), respectively modulated as $\sin{(\vartheta)}$ and $\cos{(\vartheta)}$. We can use them to generate an error signal that can lock the homodyne measure to an arbitrary angle α. In fact, upon rescaling $P_{\mathrm{cos}}^{\mathrm{err}}$ by a factor S to match the amplitude of $P_{\mathrm{sin}}^{\mathrm{err}}$, we can combine them in a sum with weights A_α, B_α such that $A_\alpha^2 + B_\alpha^2 = 1$. Hence, the final error signal will be:

$$P_{\mathrm{err}}^{\alpha} = A_\alpha P_{\mathrm{sin}}^{\mathrm{err}} + B_\alpha S P_{\mathrm{cos}}^{\mathrm{err}} \sim \sin(\vartheta + \alpha), \tag{13}$$

where $\alpha = \arctan{(B_\alpha/A_\alpha)} = \arccos{(A_\alpha)} = \arcsin{(B_\alpha)}$. In what follows, for notation simplicity, we define $\theta = \vartheta + \alpha$.

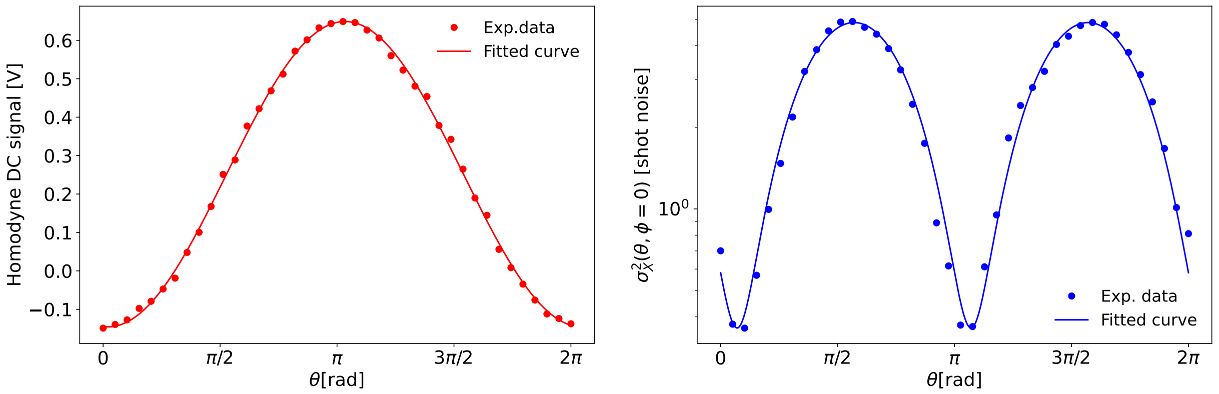

Supplementary Figure 3. **Phase-locked measurements.** In panels **(a)** and **(b)** are reported, the homodyne DC signal (red dots) and the quadratures variance (blue dots) acquired for different phase-locked homodyne angles spanning the range $\theta \in [0, 2\pi]$, respectively. Both experimental datasets have been acquired by averaging over $200,000$ acquisitions, but in the second case, a band-pass filter around 1.1 MHz is also required, especially to remove low-frequency noise. We report the corresponding fitted curves, as red and blue solid lines for the homodyne DC and quadratures variance, respectively. The former employs a cosine model, while the latter uses the lossy variance model for the quadrature variance defined in Eq.(4) of the main text.

To experimentally test this procedure, we lock the homodyne measure to different $\theta \in [0, 2\pi]$ and, for each of them, we measure the resulting homodyne DC signal and the quadrature variance, and report the corresponding results in the left and right panels of Supplementary Fig.3, respectively. The regularity of these measurements demonstrates the reliability of this approach, and enables, by fitting the experimental data, to finely calibrate possible overall offsets of the phase θ.

SUPPLEMENTARY NOTE 3. ADDITIONAL EXPERIMENTAL DATA AND PRE-CALIBRATION MEASUREMENTS

In this Section, we describe additional measures relative to the results reported in the main text. To better understand the effects of adaptive feedback, we report in Supplementary Fig.4 the quadratures measured for a specific phase ϕ during the estimation protocol. We can observe how a small portion of the total acquired data (illustrated with a yellow background in Supplementary Fig.4) is sufficient to have a rough estimation, precise enough to compute a feedback that shifts the LO phase towards the optimal one, noticeable by the substantial decreasing of the quadrature variance in the second stage of the estimation (depicted in Supplementary Fig.4 with a purple background).

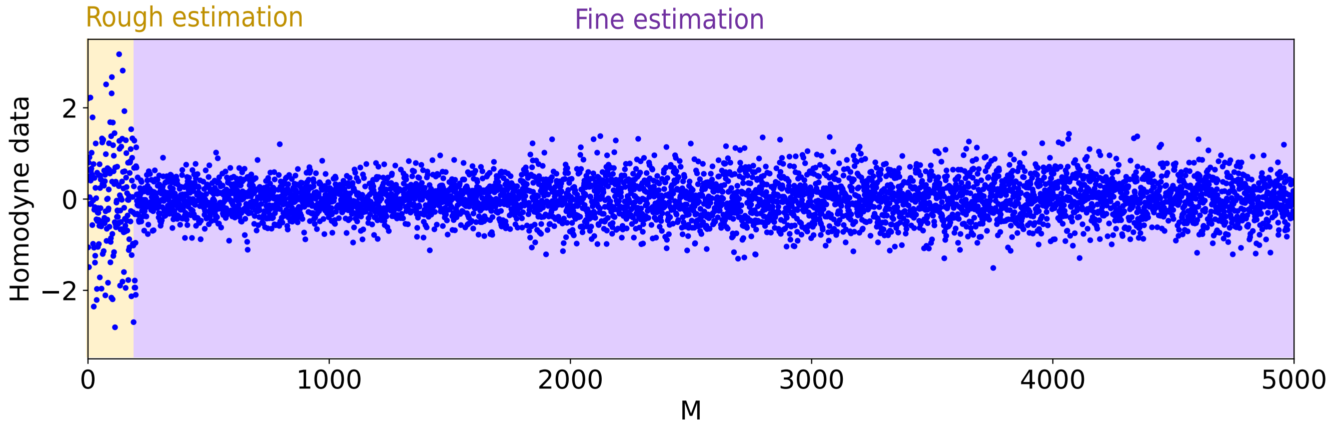

Supplementary Figure 4. **Homodyne data during the adaptive protocol.** Here we plot the homodyne measurements taken during the single-parameter estimation of the phase $\phi = 1.1$ rad. The normalized measurements are reported in such a way to fix the shot-noise to the conventional value $\sigma^2_{\text{shot}} = 1/4$. The yellow background indicates the fraction of measurements employed to obtain the phase rough estimation necessary to compute the feedback for the LO phase. Its effect is noticeable in the second stage of the measurement, illustrated on a purple background, where the homodyne data exhibits a smaller variance, corresponding to measuring along the optimal quadrature angle.

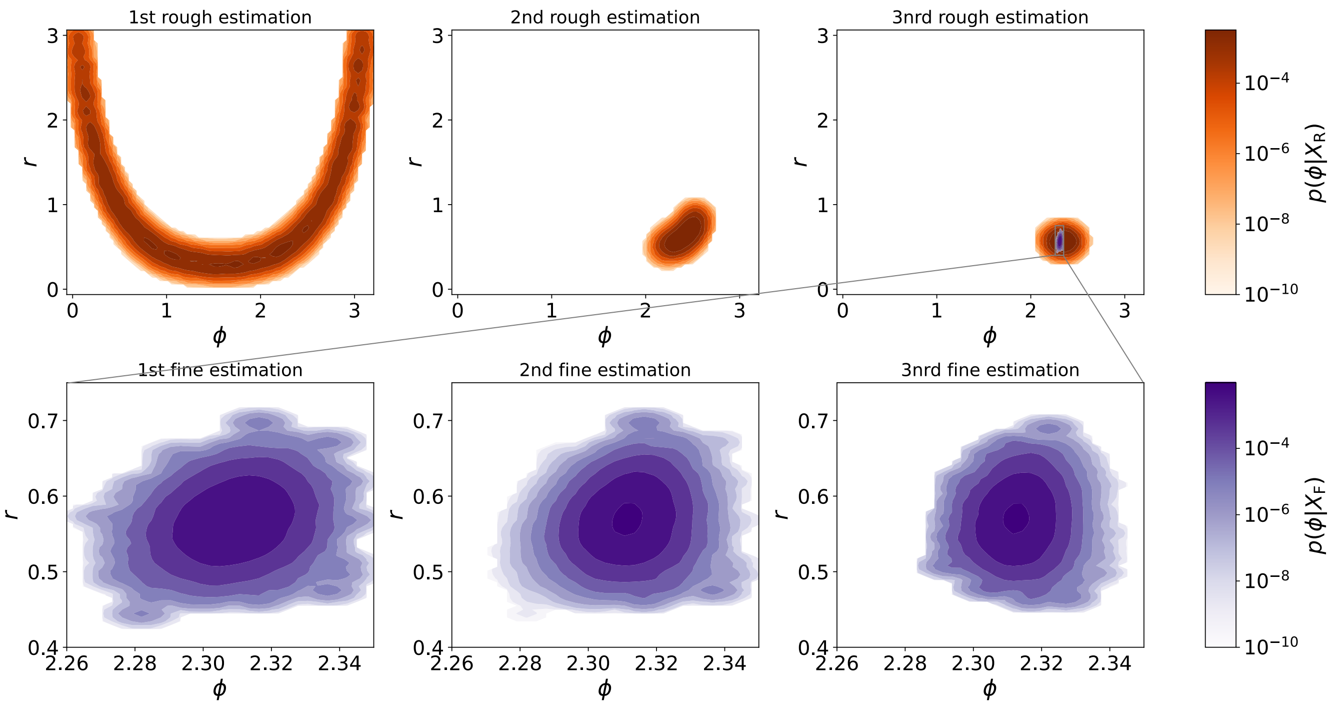

Supplementary Figure 5. **Evolution of the experimental posterior distribution during the multiparameter adaptive protocol.** In this figure, the posterior distribution $p(\phi, r|\boldsymbol{x})$ is updated during the different steps of the adaptive protocol. In the upper panels, we report the posterior during the three steps of the rough estimation, using an orange color scale. Instead, the three bottom panels illustrate the posterior evolution during the fine estimate (purple color scale), again subdivided into three adaptive feedback steps.

In the case of multiparameter estimation, we can gain further insights into the functioning of the adaptive estimation by studying how the experimental 2-dimensional posterior $p(\phi, r|\boldsymbol{x})$ evolves during the different steps of the protocol, illustrated in Supplementary Fig.5. After the first step of the rough estimation (upper left panel in Supplementary Fig.5), taken with the LO phase set at $\theta = 0$, the posterior presents a symmetric behavior along the phase. This feature reflects the fact that a homodyne data taken in a single setting cannot disambiguate the phase between a phase ϕ and $\pi - \phi$. The second step of the protocol (upper central panel in Supplementary Fig.5) updates the previous posterior with quadratures measured along $\theta = \pi/4$, and illustrates how this additional measurement setting resolves the ambiguity mentioned above. The third part of the rough estimation, by setting $\theta = \pi/2$, probes the orthogonal quadrature with respect to the first step, improving, as we can see in the upper right panel of Supplementary Fig.5, the estimation of the squeezing parameter. A sufficiently precise estimate of r is essential for computing accurate adaptive feedback, whose effect becomes clear when comparing the final rough estimate with the initial fine estimation (purple color scale). The lower panels show the posterior distributions for the three steps of the fine estimation on a magnified scale, illustrating how much of the overall precision gain is attributable to the adaptive feedback.

To quantify the protocol ability to estimate the phase ab-initio over the full $[0, \pi)$ range, the estimated values of the 10 different investigated phases $\hat{\phi}$ are compared in Supplementary Fig.6 to the true calibrated values ϕ for all the inspected strategies. Starting from the experimental single-parameter estimate, the estimated values of the two-parameter ones, and the black box variant of the protocol that also infers the overall detection efficiency η from the data, are here reported.

The scaling of the phase variance for the black-box protocol, which jointly estimates (ϕ, r, η), as a function of the number of measurements is reported in Supplementary Fig.8. Even in this case, the implemented approach enables a phase estimate below the coherent bound, confirming that the information gain provided by squeezing is retained even while the squeezing parameter and the overall efficiency are inferred on the fly and not pre-calibrated.

In terms of scaling with the average number of photons in the probe, in the ideal lossless case, the variance of the phase estimate retrieved with coherent probes scales at the SQL, $\mathrm{Var}_\phi[\hat{\phi}] \sim \frac{1}{4\langle n\rangle}$. On the contrary, squeezed probes attain a quantum-enhanced scaling $\frac{1}{8\langle n\rangle(\langle n\rangle+1)}$ that for $\langle n\rangle >> 1$ attains the Heisenberg limit. Such behaviors are altered in the presence of losses, therefore, when $\eta < 1$ the asymptotes change, obtaining bounds with slower power laws as emerges in the plot in Supplementary Fig.8. However, even for realistic lossy conditions, squeezed probes retain an advantage over coherent probes, and this is the region where we work experimentally.

In the single parameter strategy, the probe and system parameters r and η, have to be pre-calibrated. In practice, this requires collecting additional homodyne data before the phase-estimation run. This stage represents a genuine metrological cost because

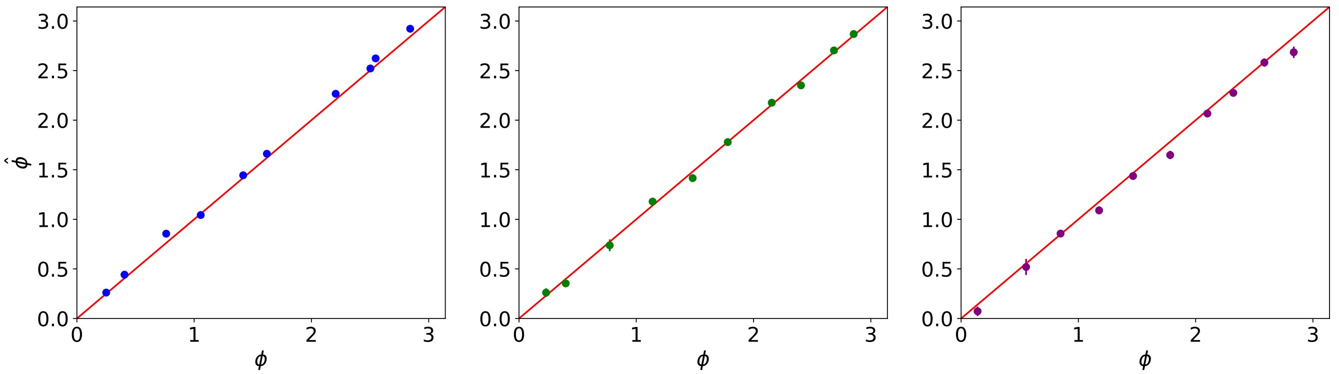

Supplementary Figure 6. **Estimated phases compared to the calibrated phase values.** In the three panels, we report the estimated phase values $\hat{\phi}$ as a function of the actual phase ϕ, respectively for the single parameter (left panel, blue dots), 2-parameter (central panel, green dots), and 3-parameter (right panel, purple dots) protocols.

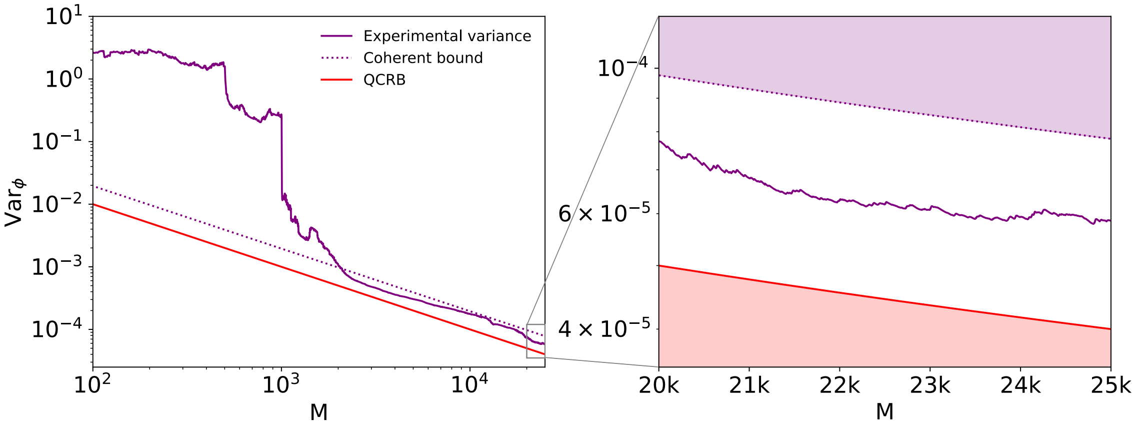

Supplementary Figure 7. **Scaling of the phase estimation variance as a function of the number of measurements, in the case of the 3-parameter estimation protocol.** The normalized variance $\widetilde{\mathrm{Var}}_\phi[\hat{\phi}] \equiv \mathrm{Var}_\phi[\hat{\phi}] \cdot MF_Q^{\mathrm{sq}}$ is reported as a function of the homodyne measurements M and compared to the QCRB (solid red line). The dotted line represents the coherent bound for the lossless scenario.

these *extra* samples do not directly contribute to estimating the parameter of interest. To quantify the amount of required *extra* resources, we determine, from experimental measurements, the relative uncertainty on the calibtation measurements of r and η as a function of employed homodyne samples M_{calib} employed in this pre-calibration stage. The results reported in Supplementary Fig.9 show that achieving a relative error, on both measurement of r and η, below 1% requires employing at least $M_{\mathrm{calib}} = 5000$ samples. A perfect calibration is instead obtained for M_{calib} larger than $40k$. This makes explicit that, even under stable conditions, obtaining r and η values needed to retrieve the likelihood and the optimal measurement projection assumed in single-parameter benchmarking entails a non-negligible measurement cost.

To highlight the operational consequence, we therefore re-evaluate the single-parameter quantum and classical bounds under an explicit resource accounting in which the total number of detected samples is $M_{\mathrm{tot}} = M + M_{\mathrm{calib}}$. As shown in Supplementary Fig.10, this shift away the experimentally evaluated variances from the ultimate QCRB computed considering M_{tot}. In particular, requiring a relative error on both r and η of the order of 1% requires an amount of calibration measurements M_{calib} that, if included in the overall resource budget, when computing the bounds whases away the quantum enhanced performances achieved in this scenario. This is shown by the fact that in this configuration the experimental points lie on the blue classical coherent bound.

By contrast, our multiparameter strategy has no separate pre-calibration stage: the relevant nuisance parameters are inferred online from the same measurement record used to estimate ϕ. In this scenario, we already include in the reported performance and in the corresponding bounds the total resource consumption. All detected samples contribute simultaneously to updating the inference model and to steering the adaptive measurement toward maximal information. This removes the structural separation between calibration and sensing that can otherwise inflate apparent performance when calibration resources are omitted, and it directly illustrates the operational advantage of a genuinely calibration-free protocol.

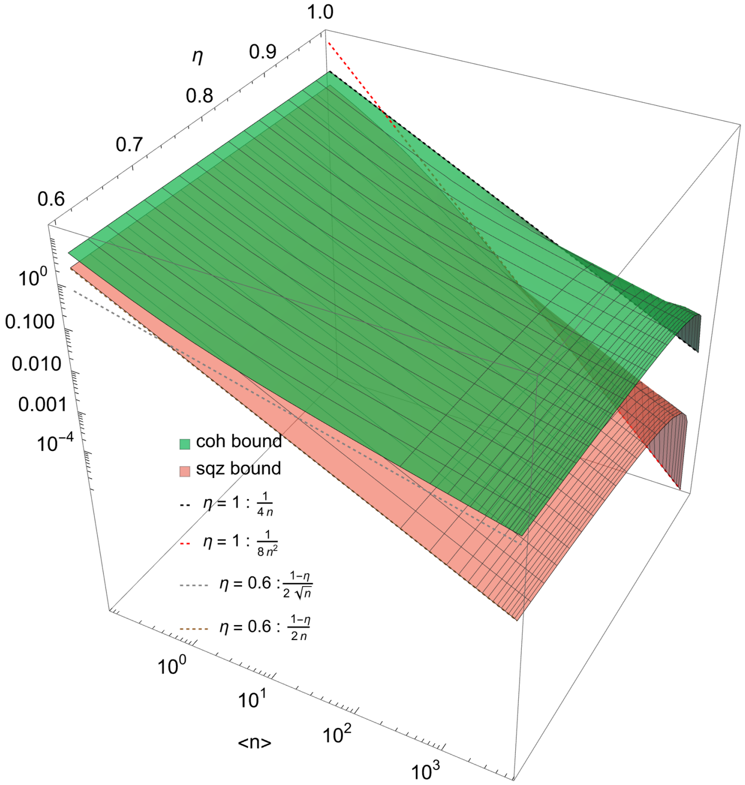

Supplementary Figure 8. **Scaling of the phase estimation variance as a function of the probe average photon number.** The ultimate phase–variance bounds for coherent (green surface) and squeezed (red surface) probes as functions of the mean photon number $\langle n \rangle$ and total efficiency η are reported. In the lossless limit ($\eta = 1$), coherent light follows the SQL, while squeezed probes achieve a quadratic scaling. Loss degrades these scalings.

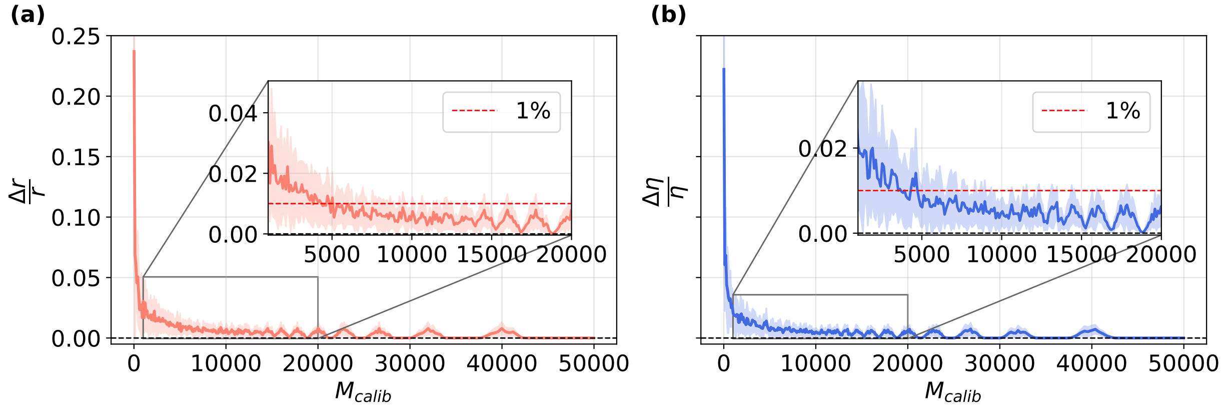

Supplementary Figure 9. **Resources for pre-calibration.** The experimental relative error of the pre-calibrated squeezing parameter r and overall efficiency eta values as a function of the number of processed homodyne samples M_{calib}, are reported in panel **(a)** and **(b)**, respectively. Insets highlight the 1% relative-error threshold (red dashed line). The black dashed line denotes the perfect estimate.

SUPPLEMENTARY NOTE 4. BOUNDS ON SIMULTANEOUS ESTIMATION OF PHASE AND SQUEEZING LEVEL

Quantum Fisher Information and Compatibility Conditions- In order to find the ultimate precision bound for the estimation of multiple parameters $\vec{y} = \{y_i\}_{i=1}^{P}$ from a given quantum state ρ, we have to compute the corresponding Quantum Fisher Information (QFI) matrix $\boldsymbol{F_Q}$. It defines the inequality chain reported in Eq.(4) in the main text, which, for the sake of clarity,

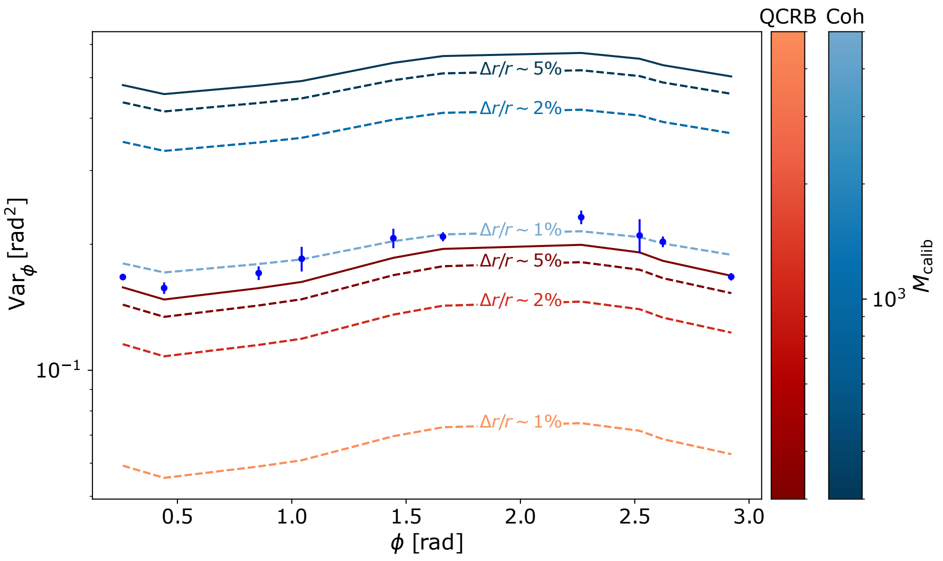

Supplementary Figure 10. **Effects of calibration resources accounting on the estimation variance bounds.** The experimental estimation variances for the single-parameter protocol are reported as blue dots for different values of the estimated ϕ. If the resources needed to pre-calibrate the nuisance parameters are not accounted for, the resulting QCRB and coherent bound are represented by solid blue and red lines, respectively, the same as reported in the main text. We report here as dashed lines in shades of red and blue, the bounds computed including also the resources needed for calibration. This shows how the QCRB and coherent bound change according to three different levels of relative error in the pre-calibration of the squeezing strength, i.e. $\Delta r/r \sim 5\%$, 2%, and 1%.

we also rewrite here:

$$\mathbf{\Sigma}[\vec{y}] \overset{\text{CRB}}{\succeq} \frac{1}{M}\boldsymbol{F}^{-1}[\{y_i\}_{i=1}^{P}] \overset{\text{QCRB}}{\succeq} \frac{1}{M}\boldsymbol{F}_{\boldsymbol{Q}}^{-1}. \tag{14}$$

In general, the elements of the QFI matrix can be computed as:

$$(\boldsymbol{F}_{\boldsymbol{Q}}[\rho])_{ij} = \frac{1}{2}\,\mathrm{Tr}\left(\rho\{L_i, L_j\}\right), \tag{15}$$

where the curly brackets denote the anticommutator and L_i is the so-called Symmetric Logarithmic (SLD) operator corresponding to the estimation of y_i. This operator is indirectly defined by the equation:

$$\frac{\partial \rho}{\partial y_i} = \frac{1}{2}\left(L_i\rho + \rho L_i\right), \tag{16}$$

and plays a crucial role in quantum metrology, since not only it is needed for the computation of the QFI, but it also defines the possibility of actually saturating the QCRB when $\mathrm{Tr}[\rho[L_i, L_j]] = 0$. Let us consider parameters that are encoded in a pure state $\rho = |\psi_{\vec{y}}\rangle\langle\psi_{\vec{y}}|$ by means of unitary transformations $|\psi_{\vec{y}}\rangle = U(\vec{y})\,|\psi_0\rangle = e^{-i\sum_i y_i G_i}\,|\psi_0\rangle$, where G_i is the generator of the i-th transformation. The computation of the SLDs and the QFI is simplified as follows:

$$L_i = -2i[G_i, \rho] \tag{17}$$

$$(\boldsymbol{F}_{\boldsymbol{Q}}[|\psi_{\vec{y}}\rangle])_{ij} = 4\left(\langle G_i G_j\rangle - \langle G_i\rangle\langle G_j\rangle\right), \tag{18}$$

where the expectation values are taken over the state $|\psi_{\vec{y}}\rangle$.

In our case, we consider a model in which the parameters of interest describe a pure squeezed vacuum state, featuring a squeezing level $r > 0$ and rotated by a phase $\phi \in [0, \pi]$. The encoding of both these parameters can be modeled as the following unitary transformation of the vacuum state $|0\rangle$:

$$|\phi, r\rangle = e^{i(\phi G_\phi + r G_r)}\,|0\rangle\,, \quad \text{where} \quad G_\phi = n = a^\dagger a,\; G_r = \frac{i}{2}\left(a^{\dagger 2} - a^2\right). \tag{19}$$

Then, the QFI corresponding to the operations reported in Eq.(19) can be computed by means of Eq.(18), where we substitute $\vec{y} = \{\phi, r\}$, obtaining the following matrix:

$$\boldsymbol{F}_{\boldsymbol{Q}}[|\phi, r\rangle] = \begin{pmatrix} 2\sinh^2(2r) & 0 \\ 0 & 2 \end{pmatrix}. \tag{20}$$

The diagonal form of the QFI in Eq.(20) further simplifies the evaluation of ultimate precision bounds on the estimation of ϕ and r, which ultimately reduces to the inverse of the corresponding diagonal elements.

In a multiparameter setting, two parameters are said to be compatible if there exists a measurement strategy that can simultaneously saturate the QCRB for both parameters. A sufficient condition to achieve compatibility is the commutation of the SLDs corresponding to the parameters of interest, as projective measurements onto their common eigenbasis enable the optimal estimation of such parameters. When the state ρ is pure, as in our model, a weaker necessary and sufficient condition can be verified, i.e. the so-called *weak commutation relation*, which is verified $\langle [L_i, L_j] \rangle = 0$. In our model, where the parameters are encoded via unitary transformation this expression further simplifies, and can be evaluated as follows:

$$\langle [L_\phi, L_r] \rangle = 4 \langle [G_\phi, G_r] \rangle = -4i \sinh(2r) \neq 0. \tag{21}$$

This means the parameters ϕ and r are incompatible, i.e. we cannot devise a measurement strategy that simultaneously achieves the optimal precision in both estimations.

In what follows, we describe how adaptivity can exploit the multiparameter estimation framework to approach the phase estimation QCRB when the squeezing level is unknown.

Adaptive protocol model- The likelihood $p(x|\theta, \phi, r)$ for a homodyne measurement with local-oscillator phase θ on a squeezed state (phase ϕ, squeezing r, transmission η) is modeled as a zero-mean Gaussian whose variance is the one reported in Eq.(4) of the main text, which we also reproduce here for convenience.

$$\sigma^2(\varphi, r, \eta) = \frac{1}{4}\big(\eta e^{-2r} \cos^2 \varphi + (1-\eta) \cos^2 \varphi + e^{2r} \sin^2 \varphi\big), \tag{22}$$

where $\varphi = \theta - \phi$.

In what follows, we describe the 2-parameter adaptive protocol, deriving the model of the corresponding Fisher Information (FI) and, then, its CRB.

In particular, in a multiparameter framework where $\vec{y} = \{\phi, r\}$ are the parameters to be estimated, the individual elements of the FI matrix are computed as:

$$F_{ij}(\vec{y}|\theta, \eta) = \int_{-\infty}^{\infty} \mathrm{d}x \; p(x|\vec{y}) \left(\frac{\partial}{\partial y_i} \log p(x|\vec{y}, \theta, \eta) \right) \left(\frac{\partial}{\partial y_j} \log p(x|\vec{y}, \theta, \eta) \right). \tag{23}$$

In this case, η must be determined by calibration, whereas in the three-parameter estimation protocol it is unknown and therefore included in the data vector $\vec{y}$.

If we consider a single-setting experiment in which homodyne data are acquired at a single LO phase, then the resulting FI matrix is singular and the corresponding CRBs are not defined. This is unsurprising: measuring only one quadrature does not provide enough information to distinguish different combinations of ϕ and r , i.e., the measurement settings are not tomographically complete. A first achievement of the adaptive protocol we describe is the removal of this ambiguity, which in turn allows one to derive a meaningful multiparameter CRB.

As discussed in Supplementary Note 4, ϕ and r are incompatible parameters and cannot be estimated simultaneously with optimal precision. Given that our aim is to choose the optimal setting ϕ_{opt} for estimating ϕ, we expect r to be estimated with suboptimal precision at that setting. In fact, below we show that the optimal setting for ϕ corresponds to the point of minimal sensitivity for r. Since the adaptive feedback relies on $\hat{r}$, poor estimation of r also degrades the precision of $\hat{\phi}$; to mitigate this, we design the adaptive protocol accordingly. A natural strategy is to steer the LO phase towards the setting that removes the correlation between the estimation errors, or, equivalently, to drive the off-diagonal element of the FI matrix to zero. This yields

$$\theta_{\text{opt}} = \hat{\phi} - \phi_{\text{opt}} = \hat{\phi} - \arccos\left(\frac{e^{2\hat{r}}}{\sqrt{e^{4\hat{r}} + \eta}} \right), \tag{24}$$

where $\hat{\phi}$ and $\hat{r}$ are the current parameter estimate before the adaptive feedback.

Keeping in mind this strategy, we now develop a FI matrix model that replicates our multiparameter adaptive protocol. Firstly, we recall the strategy we have implemented experimentally:

- *Rough estimation*: the first $M_R = 1,200$ homodyne measures are devoted to obtain approximate estimations of the parameters. In particular, we equally divide them in measures with LO phase in $\theta = \{0, \pi/4, \pi/2\}$. This resource allocation in equally spaced settings has the twofold objective of eliminating the aforementioned singularity and provide a sufficiently precise estimation of both parameters.

- *Adaptive step*: the estimated parameters $\hat{\phi}$ and $\hat{r}$ are employed to compute θ_{opt}, and shift the LO phase to this value. In the actual experimental implementation, this step can also be reiterated multiple times to finely adjust the homodyne setting to the optimal value.

- *Fine estimation*: as further discussed in Supplementary Note 6, a sufficient amount M of homodyne measures has to be acquired to achieve statistical convergence. In our case, $M = 20,000$ is sufficient to ensure such a convergence. Therefore, the remaining $M_F = M - M_R = 18,800$ homodyne data are measured within the optimal setting to estimate ϕ. As we will see, this strategy allows to closely approach the phase QCRB.

Therefore, using the definition reported in Eq.(22), we can model the FI matrix corresponding to the adaptive protocol as a function of the LO phase in the fine estimation step:

$$\widetilde{\boldsymbol{F}}(\phi, r|\theta, \eta) = \underbrace{\left(\frac{1}{3}\boldsymbol{F}(\phi, r|0, \eta) + \frac{1}{3}\boldsymbol{F}(\phi, r|\pi/4, \eta) + \frac{1}{3}\boldsymbol{F}(\phi, r|\pi/2, \eta)\right)\frac{M_R}{M}}_{\text{Rough estimation}} + \underbrace{\boldsymbol{F}(\phi, r|\theta, \eta)\frac{M_F}{M}}_{\text{Fine estimation}}. \tag{25}$$

The resulting FI matrix is invertible, allowing us to define the corresponding multiparameter CRBs.

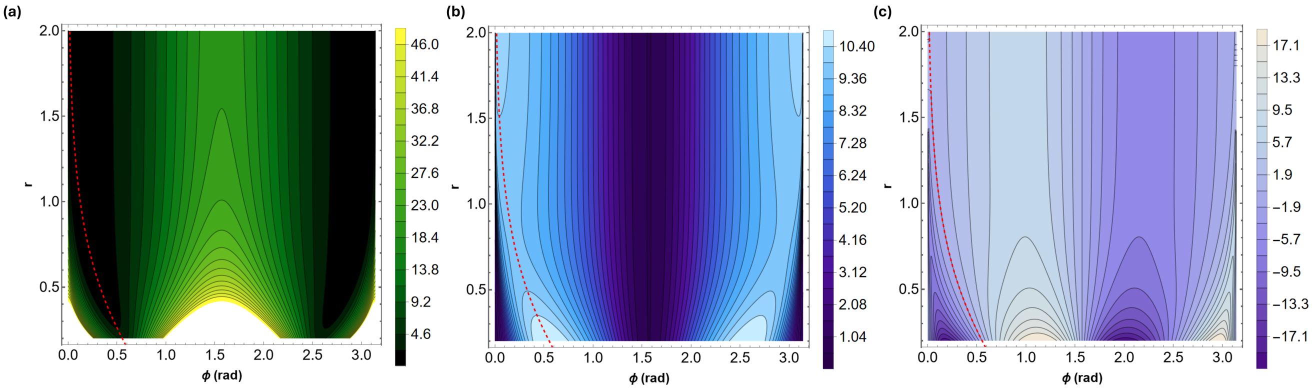

Supplementary Figure 11. **Inverse of the FI matrix elements in the multiparameter adaptive protocol**. In this figure, we report the behavior of the $\widetilde{\boldsymbol{F}}^{-1}$ entries as a function of $\phi \in [0, 2\pi]$ and $r \in [0, 2]$. In particular, $[\widetilde{\boldsymbol{F}}^{-1}]_{\phi\phi}$, $[\widetilde{\boldsymbol{F}}^{-1}]_{rr}$, and $[\widetilde{\boldsymbol{F}}^{-1}]_{\phi r} = [\widetilde{\boldsymbol{F}}^{-1}]_{r\phi}$ illustrated respectively in panels **(a)**, **(b)**, and **(c)**. In all of them, we report the value of the phase $\phi_{\text{opt}}(r, \eta)$ that eliminates the correlations as a red dashed line.

In Supplementary Fig.11, we report the behavior of the individual elements of $[\widetilde{\boldsymbol{F}}^{-1}]$ as a function of the parameters ϕ and r. For the sake of clarity, in what follows, we will use the notation $[\widetilde{\boldsymbol{F}}^{-1}]_{ij}$ with $i, j \in \{\phi, r\}$ to denote corresponding entries of the inverse FI matrix. In particular, we can notice how the r-dependent phase that eliminates the correlations (red dashed lines in Supplementary Fig.11), i.e. $[\widetilde{\boldsymbol{F}}^{-1}]_{\phi r} = 0$ (right panel) also corresponds to the minimum of the $[\widetilde{\boldsymbol{F}}^{-1}]_{\phi\phi}$ (left panel) and the maximum $[\widetilde{\boldsymbol{F}}^{-1}]_{rr}$ (central panel), along the entire range of r.

To have a better understanding of the adaptive FI matrix model we derived, in Supplementary Fig.12, we illustrate the behavior of $[\widetilde{\boldsymbol{F}}^{-1}]_{\phi\phi}$, $[\widetilde{\boldsymbol{F}}^{-1}]_{rr}$, and $[\widetilde{\boldsymbol{F}}^{-1}]_{\phi r}$ when the squeezing level and efficiency are similar to the experimental conditions, i.e. $r = 0.63$ and $\eta = 0.85$. As previously mentioned, we can see how the setting that maximizes the precision in estimating ϕ (r), at the same time minimizes the sensibility to r (ϕ). Additionally, the inset (a) of Supplementary Fig.12 shows how the ϕ_{opt} is indeed the one that removes the correlation (purple curve) and optimizes $[\widetilde{\boldsymbol{F}}^{-1}]_{\phi\phi}$, which achieves a value quite close to the corresponding QCRB. This consideration is also true for the optimal estimation of r, which is detailed in the inset (c) of Supplementary Fig.12.

To directly connect the model to the experiment, Supplementary Fig.13 compares the theoretical bounds computed in this section with the posterior variances reconstructed from the experimental data. Specifically, considering the experimentally implemented configuration that optimizes phase estimation precision, we observe that the posterior variances are compatible with the adaptive protocol CRBs for both ϕ (panel **(a)**) and r (panel **(b)**), and the phase estimation variances remain significantly below the coherent bound. In addition, in panel **(c)** we report the experimentally estimated correlation $\text{Corr}[\hat{\phi}, \hat{r}] = \text{Cov}[\hat{\phi}, \hat{r}]/\sqrt{\text{Var}_\phi[\hat{\phi}]\text{Var}_r[\hat{r}]}$ for all the measured phases, finding an overall average value of $\overline{\text{Corr}}_{\phi r} = -0.085 \pm 0.037$. Such a small correlation is in agreement with the model prediction (see Supplementary Fig.12b), according to which the phase-optimized operating point of the multiparameter protocol also corresponds to a near-vanishing off-diagonal term of the inverse FIM. Furthermore, panel **(d)** depicts a representative experimental posterior distribution, which directly visualizes the anisotropic uncertainty expected from the multiparameter trade-off: the posterior is more tightly localized along the phase direction (the parameter prioritized by the feedback) and broader along the squeezing direction. This provides an intuitive picture of how the protocol allocates information between phase estimation and probe self-calibration.

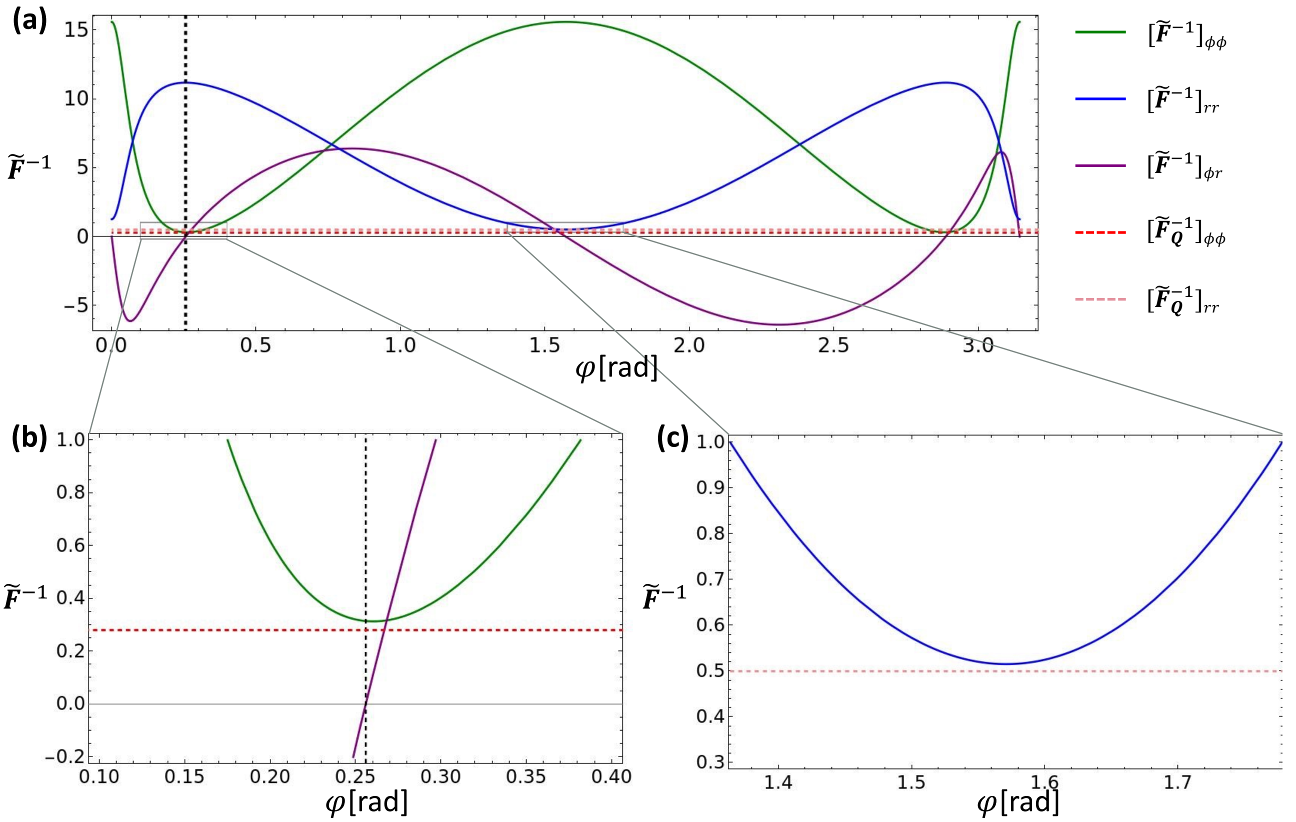

Supplementary Figure 12. **Adaptive FI matrix model for experimental values of r and ϕ.** In this figure, we report the behavior of the adaptive FI matrix entries as a function of $\varphi = \theta - \phi$, when considering the experimentally plausible parameters $\phi = \pi/4$, $r = 0.63$, and $\eta = 0.85$. In the main panel **(a)**, we report the $[\widetilde{\boldsymbol{F}}^{-1}]_{\phi\phi}$ (green curve), $[\widetilde{\boldsymbol{F}}^{-1}]_{rr}$ (blue curve), and $[\widetilde{\boldsymbol{F}}^{-1}]_{\phi r}$ (purple curve) for the entire range $\varphi \in [0, 2\pi]$, comparing them to the corresponding QCRBs for the estimation of ϕ, i.e. $[\boldsymbol{F}_{\boldsymbol{Q}}^{-1}]_{\phi\phi}$ (red dashed line), and for the estimation of r, i.e. $[\boldsymbol{F}_{\boldsymbol{Q}}^{-1}]_{rr}$ (orange dashed line). The black dashed line represents the phase ϕ_{opt} that removes the correlation between the estimation errors of ϕ and r. In the insets **(b)** and **(c)**, we report the magnification of the main plot around the optimal settings for estimating ϕ and r, respectively, again compared to the corresponding QRCBs.

Finally, we further motivate the use of the multiparameter approach through numerical simulations showing that the implemented protocol is inherently robust to variations in the probe squeezing level, whereas the single-parameter approach fails under the same conditions. The results of simulations performed by changing the squeezing by an amount Δr are reported in Supplementary Fig. 14. The plotted results refer to the variance on the phase estimate for a single-parameter estimator that fixes the squeezing at its pre-calibrated value r, and our multiparameter adaptive estimator, as the true squeezing is offset by Δr. For the single-parameter approach, even small mismatches brings to unphysical variances or significantly degrade estimation precision. The estimation error approches the bound only when the effective squeezing remains very close to the calibrated value. On the contrary, the proposed multiparameter adaptive protocol is able to keep the phase estimation variance close to the QCRB for any variation of the squeezing, since it avoids explicit calibration of the probe squeezing and instead infers its value directly from the data. This joint inference makes the protocol robust, suppressing systematic errors from drift and sample variability.

SUPPLEMENTARY NOTE 5. OVERCOMING FISHER INFORMATION MATRIX SINGULARITY

The singularity of the (Quantum) Fisher Information Matrix ((Q)FIM) is a well-known limitation that can arise in multiparameter estimation problems [1], preventing the standard definition of the corresponding (Q)Cramér-Rao Bound ((Q)CRB). Several approaches have been proposed to circumvent this problem, such as the use of the Moore-Penrose pseudoinverse [2], sequential measurement strategies [3], scrambling operations [4], and step-wise estimation protocols [5, 6]. In what follows, we will show how, in our case, the FIM singularity is removed thanks to the multiple steps prescribed by the adaptive estimation protocol.

The key property of the FIM that we exploit is its additivity for independent measurements. In our case, as illustrated in Eq.(25), the overall FIM resulting from our adaptive protocol is constituted of the sum of the contributions from the different

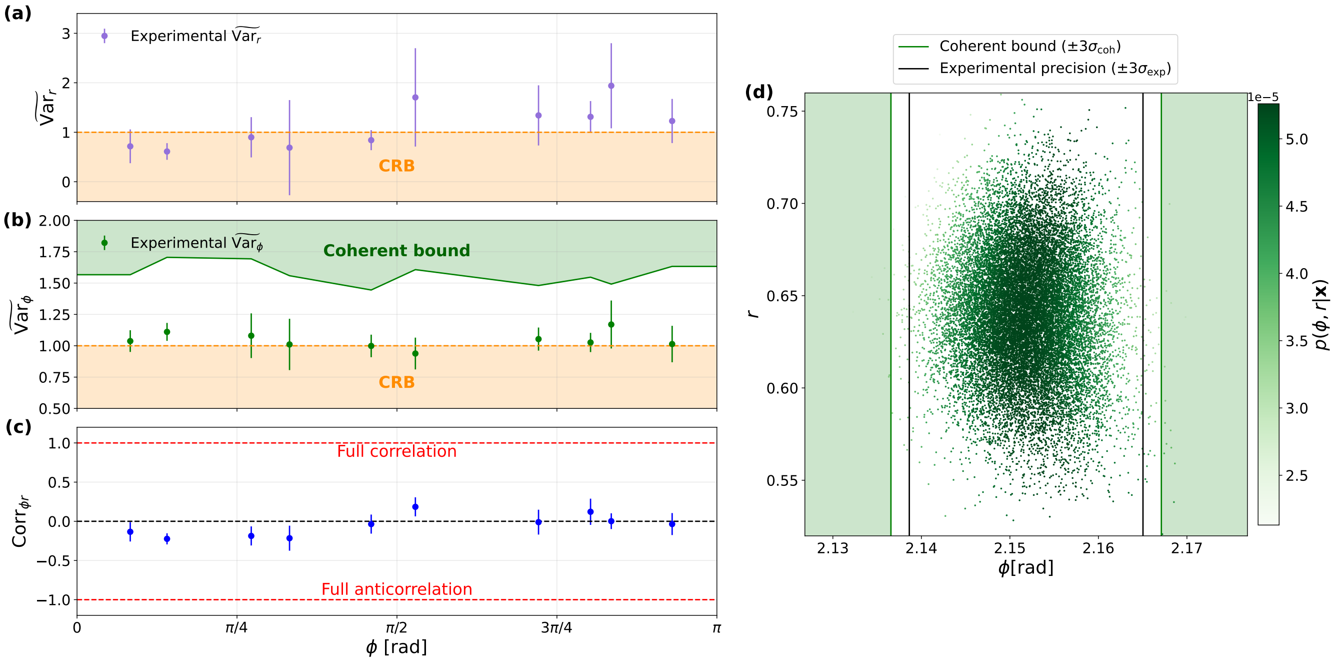

Supplementary Figure 13. **Comparison of our CRB model with experimental results.** Panels **(a)** and **(b)** display the experimental variances for the estimation of r (purple dots) and ϕ (green dots), respectively. The data are normalized to the corresponding CRB, represented by a constant dotted orange line at $\widetilde{\mathrm{Var}} = 1$, where the tilde denotes the normalization. Additionally, in panel **(b)**, the coherent bound is indicated by a solid green line. In panel **(c)**, we report the parameter correlation $\mathrm{Corr}_{\phi r} \equiv \mathrm{Cov}_{\phi r}/\sqrt{\mathrm{Var}_\phi \mathrm{Var}_r}$ obtained from the experimental multiparameter estimations, compared to the extremal values of full (anti)correlation (red dashed lines) and full lack of correlation (black dashed line). Panel **(d)** depicts a representative experimental posterior distribution (green dots) alongside the corresponding experimental standard deviation, $\sigma_{\mathrm{exp}} \equiv \sqrt{\mathrm{Var}_\phi}$, which is compared to the coherent bound standard deviation, $\sigma_{\mathrm{coh}} \equiv \sqrt{\mathrm{QCRB}_{\mathrm{coh}}}$.

steps of the protocol. Since the sum of singular matrices is not necessarily a singular matrix itself, by properly choosing the measurements to be performed during the rough estimation, it is possible to achieve a global non-singular matrix and, therefore, a well-defined CRB.

In order to have a better physical understanding of this process, we exploit the framework presented in [7] to analyze the origin of FIM singularity in our model and how it can be removed. In particular, it can be demonstrated that the FIM corresponding to the joint estimation of phase and squeezing level from measurements along a single homodyne angle always leads to a singularity. This is a consequence of the parametrization of the model, since the likelihood corresponding to the homodyne detection of squeezed vacuum state (Eq. (4) of the main text) is modeled by a zero-mean Gaussian distribution with variance depending on both the phase and squeezing level, which we recall here for practicality:

$$\sigma^2(\varphi, r, \eta) = \frac{1}{4}\left(\eta e^{-2r}\cos^2\varphi + (1-\eta)\cos^2\varphi + e^{2r}\sin^2\varphi\right), \tag{26}$$

This is indeed an instance of overparametrization, where a model with only one degree of freedom (i.e. the variance) is instead described with a larger number of parameters. For this reason, there will be different combinations of ϕ and r which will produce the same quadrature variance. In the context of Bayesian estimation, a recent work [7] points out that for such problems, even if one cannot give a proper estimate of the parameters, it is still possible to gain insights on the singularity affecting the estimation, as the posterior distribution will not converge to a single point in parameter space, but rather lie along a line. The curve describing this line represents the set of parameters for which the effective single parameter describing the problem remains constant. Even if a general approach to identify such an effective parameter is not provided, in our case, it is rather intuitive to associate it with quadrature variance, since it is the only quantity that is really fixed when measuring quadrature along a single angle. For this reason, we expect that, in our case, the posterior will lie along the "isovariance curve" describing the combinations of ϕ and r for which the variance in Eq.26 is equal to the observed quadrature variance. Examples of such isovariance curves are illustrated in Supplementary Fig.15a.

In order to verify this property, in Supplementary Fig.15b-d, we report the behavior of the posterior distribution $p(\phi, r|\eta, \theta, \mathbf{x})$ during the rough estimation step of our protocol. In particular, we simulate 400 homodyne detection for a squeezed vacuum state with parameters $\phi_{\mathrm{true}} = 3\pi/4$, $r_{\mathrm{true}} = 0.8$, and $\eta = 0.85$, repeating this operation for three different LO phases $\theta = 0, \pi/2, \pi/4$. After measuring quadratures only along $\theta = 0$, the resulting posterior is indeed "delocalized" along the curve describing the

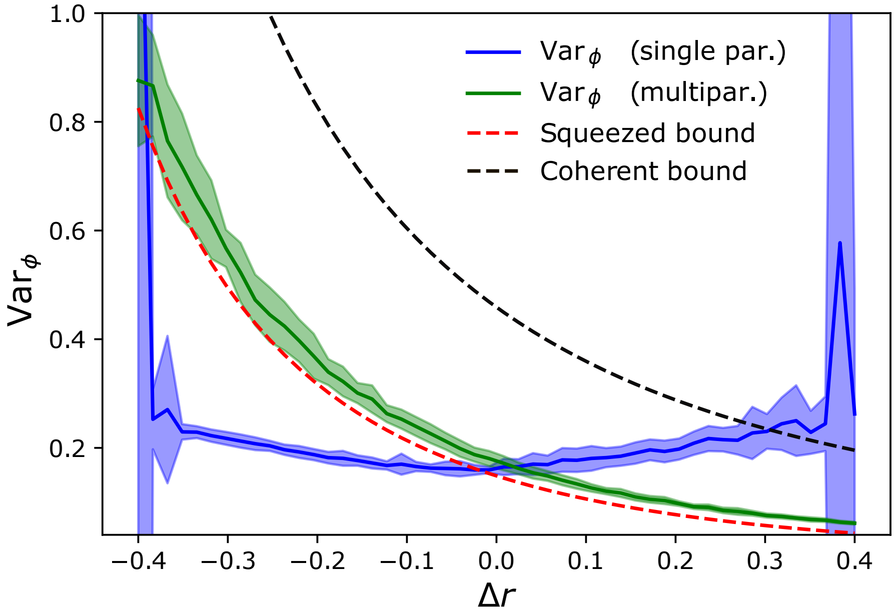

Supplementary Figure 14. **Behavior of the variance as the squeezing level deviates from the calibration.** In this figure, we report how the phase estimation variance changes when the squeezing level deviates from the original value $r = 0.8$ by a quantity $\Delta r \in [-0.4, 0.4]$. In particular, we report the average and the standard deviation (over 50 repetitions) of the simulated estimation variance obtained with the single-(multi-)parameter adaptive protocol as a blue (green) solid curve and shaded area, respectively. We compare them with the corresponding QCRB (red dashed curve) and coherent bound (black dashed curve).

combinations of parameters which produce a homodyne distribution with variance equal to $\sigma^2(\phi = \phi_{\mathrm{true}}, r = r_{\mathrm{true}}, \eta)$, as we can see in Supplementary Fig. 15. Therefore, at this stage, the estimation cannot yet provide a reliable measure of ϕ and r. In Supplementary Fig.15c, we can see how the singularity breaks after updating the posterior with an additional 400 homodyne measurements taken along $\theta = \pi/2$, since the posterior distribution is now constrained to lie in the intersection between the two isovariance curves. Even if this lifts the symmetry generating the continuous set of ambiguous pairs (ϕ, r), the estimation cannot resolve between the phases ϕ_{true} and $\pi - \phi_{\mathrm{true}}$. The final 400 homodyne data taken with $\theta = \pi/4$ also remove this ambiguity, so that, as illustrated in Supplementary Fig.15d, the posterior distribution concentrates around the only intersection point among the three isovariance curves, which correspond to the actual values of the parameters ϕ_{true} and r_{true}, therefore providing the proper estimations needed to proceed with the adaptive step of the protocol.

SUPPLEMENTARY NOTE 6. SEQUENTIAL MONTE CARLO

We estimate the unknown phase ϕ with a Bayesian procedure that updates a flat prior on $[0, \pi]$ using homodyne data $\boldsymbol{x} = \{x_m\}_{m=1}^{M}$. Such measurements are employed to reconstruct the single-parameter posterior probability distribution using Bayes' theorem, the posterior probability reconstructed after M measures is reported in Supplementary Fig.16.

The reconstructed posterior is updated using the homodyne probability distribution, obtained by marginalizing the Wigner function of the squeezed probe state and setting the phase of the local oscillator (LO) to θ. Once having reconstructed the posterior probability, we derive the estimate $\hat{\phi}$ for the parameter of interest and its variance. As usual in the Bayesian framework, these are obtained by calculating the mean value and variance of the posterior distribution, respectively:

$$\hat{\phi} = \int p(\varphi|\boldsymbol{x})\varphi d\varphi \sim \sum_{k=1}^{n_p} \omega_k(\boldsymbol{x})\phi_k, \tag{27}$$

$$\mathrm{Var}_\phi[\hat{\phi}] = \int p(\varphi|\boldsymbol{x})(\hat{\varphi} - \varphi)^2 d\varphi \sim \sum_{k=1}^{n_p} \omega_k(\boldsymbol{x})(\hat{\phi} - \phi_k)^2. \tag{28}$$

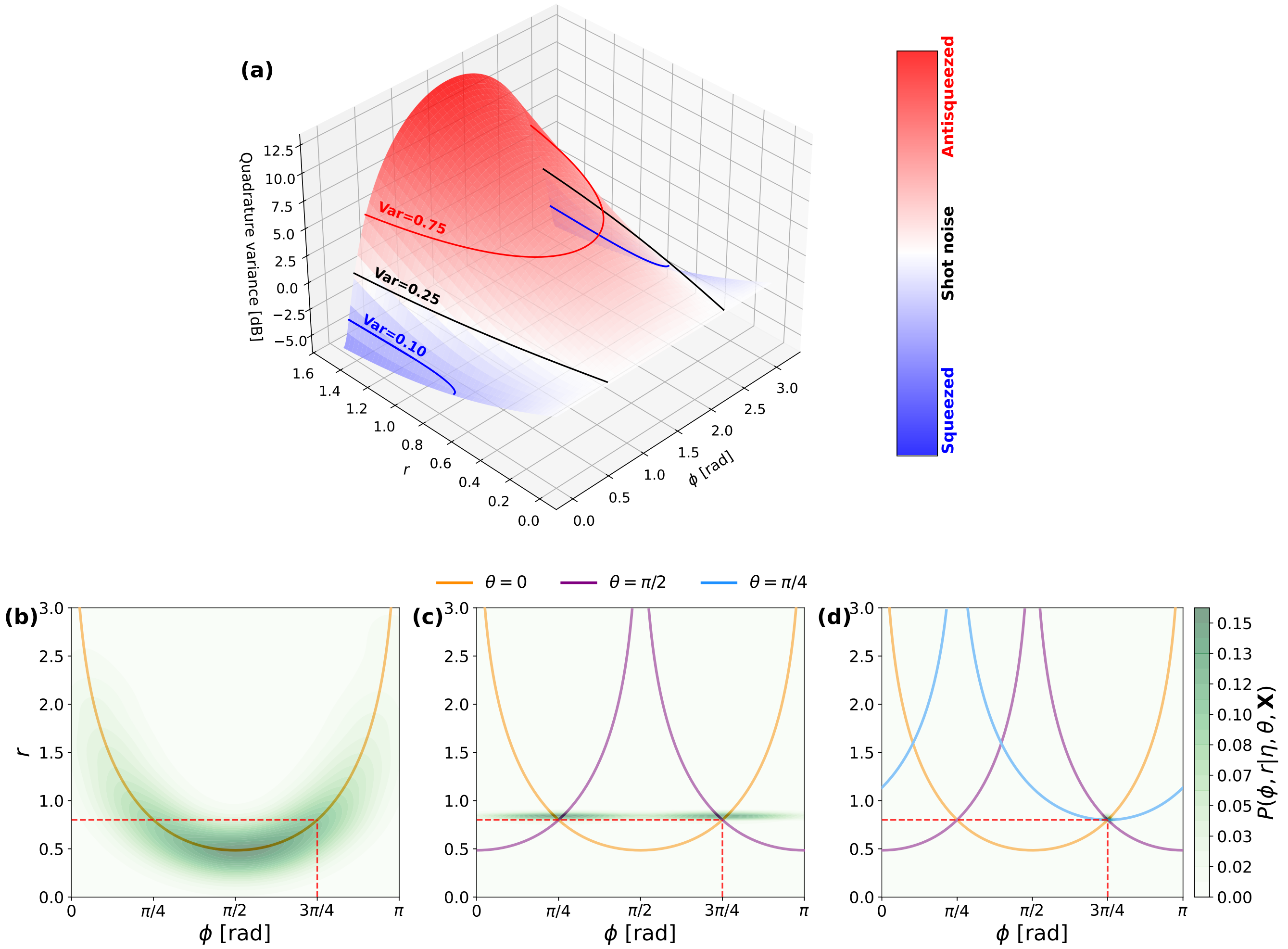

Supplementary Figure 15. **Effects of the FIM singularity on the Bayesian estimation of ϕ and r.** In panel **(a)**, we represent the behavior of the quadrature variance for the homodyne detection of a squeezed vacuum state, as a function of the phase ϕ and the squeezing strength r, while the efficiency $\eta = 0.85$ and the LO phase $\theta = 0$ are taken as fixed parameters. As examples, isovariance curves are depicted in the cases of vacuum fluctuations ($\sigma^2 = 1/4$), squeezed ($\sigma^2 = 0.1 < 1/4$), or antisqueezed ($\sigma^2 = 0.75 > 1/4$), respectively indicated by black, blue, and red solid curves. In panels **(b)**-**(d)**, we illustrate the evolution of the posterior distribution when consecutive batches of homodyne data (400 quadratures each) are acquired for different values of $\theta = 0, \pi/2, \pi/4$. For each one of these LO phases, we depict the corresponding isovariance curve (yellow, purple, and blue solid lines, respectively corresponding to $\theta = 0, \pi/2, \pi/4$), illustrating how the posterior distribution is affected by its symmetries, progressively removed as new batches of homodyne data are measured along different angles. The actual values of the parameter to be estimated are $\phi_{\text{true}} = 3\pi/4$ and $r_{\text{true}} = 0.8$, indicated by the intersection of the red dashed lines.

In the adopted framework, the computation is accelerated by substituting integrals with discretized sums that have a relevant impact, in particular for multiparameter estimation, where the computation of multidimensional integrals can be costly, affecting the realization of the adaptive protocol.

To verify that the implemented estimator attains the relevant precision limits, we perform simulations of the estimation protocols using the probe parameters $\eta = 0.8$ and $r = 0.8$. In the single-parameter protocol, we discretize $[0, \pi]$ with $n_p = 10000$ particles and observe the expected $1/M$ variance decay that saturates the QCRB. In the joint (ϕ, r) estimate, $n_p = 20000$ total particles were employed, and the trend of the variances on both parameter estimates has been investigated. The simulations have been carried out with the same adaptive protocol described before, whose aim is to minimize the variance on the phase estimate. Studying the performance scaling reported in Supplementary Fig.17 with the number of homodyne measures is evident how they saturate to the relative elements of the inverse of the FIM computed in the previous section.

Finally, we study the robustness of the implemented protocol with respect to the initial prior choice. In Supplementary Fig.18 we report the simulated phase estimation variance as a function of processed homodyne samples, when adopting different priors from the flat prior used in the experimental realization. We study the algorithm performance when starting from Gaussian

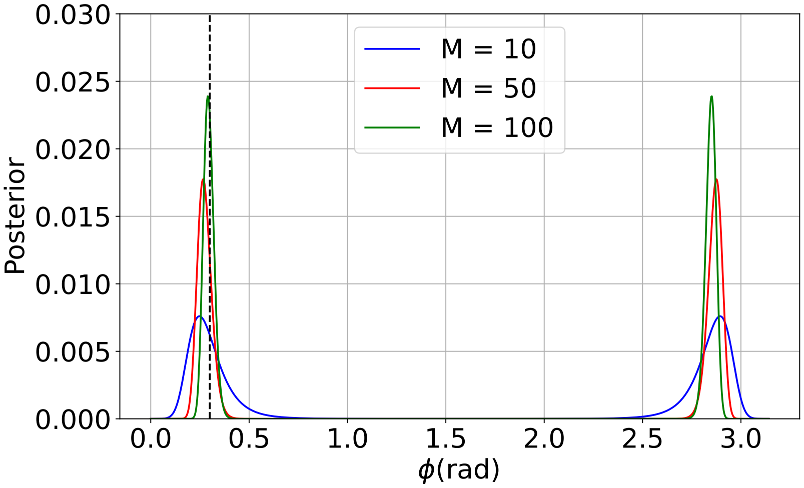

Supplementary Figure 16. **Simulated Bayesian posterior.** Reconstructed posterior probability distribution of Bayesian phase estimation in range $[0, \pi]$ with $M = 10, 50, 100$ homodyne measures. We choose a trial phase $\phi_{true} = 0.3$ rad and a squeezing parameter $r = 1$.

informed priors centred on the true values $(\phi_{\text{true}}, r_{\text{true}})$ with different widths, and for Jeffrey's based on Fisher-information weighting priors [8]. As is clear from the simulated results, the performance after a sufficiently large number of M becomes independent of the particular choice of the prior.

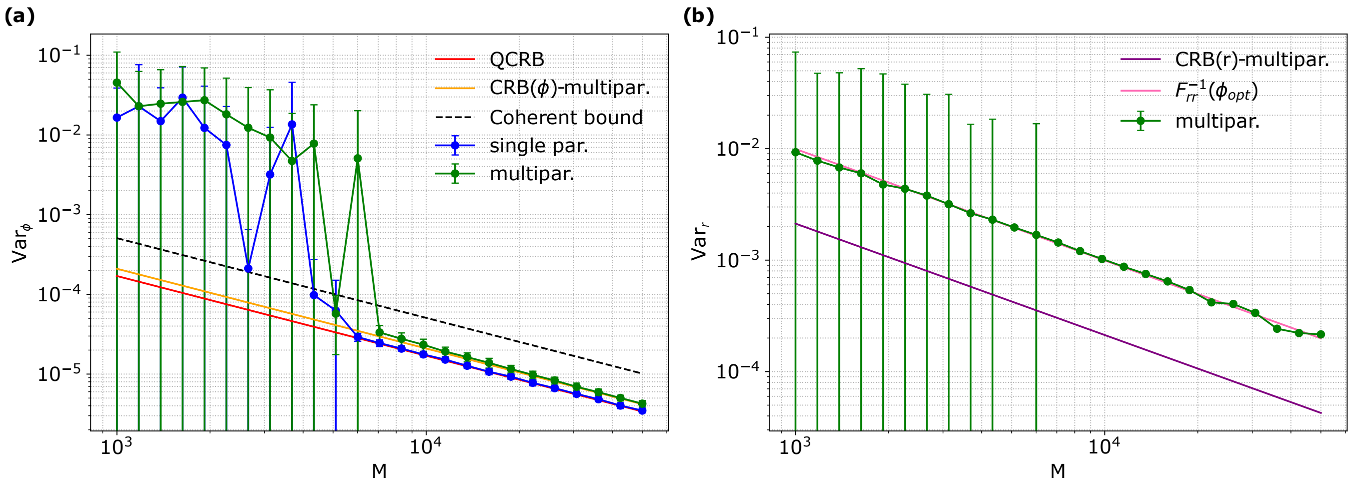

Supplementary Figure 17. **Scaling of simulated posterior variance with the number of homodyne samples M.** Panel **(a)** average variance of the phase estimate with the single-parameter Bayesian protocol (blue circles); variance of the phase estimate with the multiparameter Bayesian protocol (green circles). The dashed black line is the ultimate classical bound for coherent states, the red line is the QCRB for squeezed light; the orange line is the CRB for the adopted two-parameter strategy. Panel **(b)** shows the average variance of the squeezing parameter estimate (green circles). The purple line is the CRB achievable when the protocol is optimized to minimize $\mathrm{Var}_r[\hat{r}]$ while the pink line represents the bound obtained with the implemented (phase-optimized) adaptive strategy. Error bars represent the standard deviation over estimates of 20 different phase values.

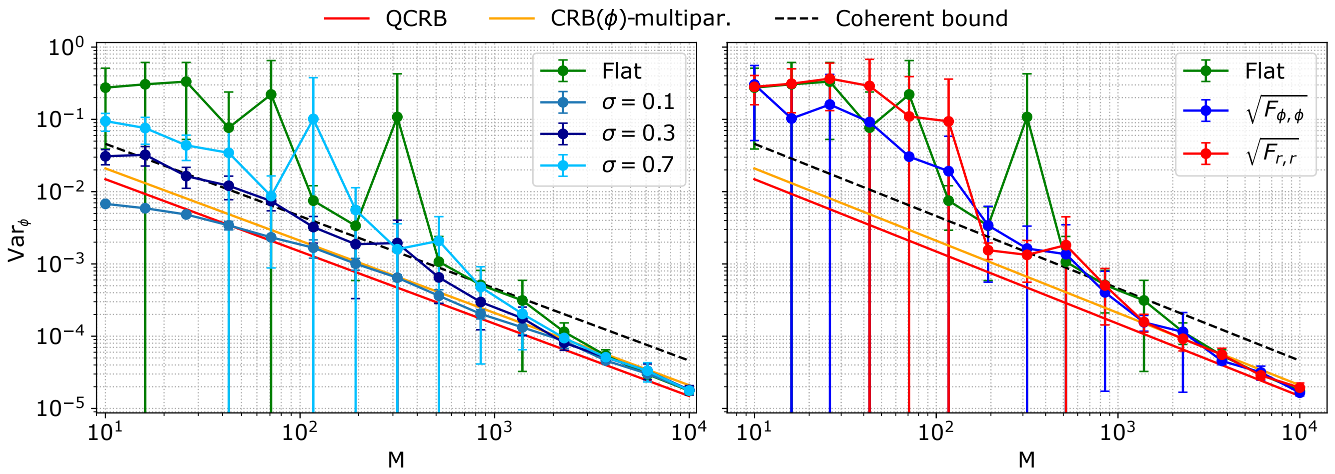

Supplementary Figure 18. textbfRobustness of phase estimation sensitivity to prior choice. Numerical simulation results for the phase estimation variance as a function of the number of homodyne samples M, comparing the flat prior used in the experiment (green) against alternative initial distributions. The left panel shows the protocol performance when adopting Gaussian informative priors with different widths (σ) and centered on the true parameters, $p(\phi, r) = \mathcal{N}(\phi_{\rm true}, r_{\rm true}, \sigma)$. The right panel reports the results for independent Jeffreys priors, $p(\phi, r) \propto \sqrt{F_{\phi,\phi}}$ and $p(\phi, r) \propto \sqrt{F_{r,r}}$. In all cases, the adaptive Bayesian update ensures that the estimator is dominated by the measurement data at large M, with the phase variance converging to the same quantum-enhanced limit regardless of the initial prior modeling.

[1] Aaron Z Goldberg, José L Romero, Ángel S Sanz, and Luis L Sánchez-Soto, "Taming singularities of the quantum fisher information," International Journal of Quantum Information **19**, 2140004 (2021).

[2] Petre Stoica and Thomas L Marzetta, "Parameter estimation problems with singular information matrices," IEEE Transactions on Signal Processing **49**, 87–90 (2002).

[3] Yaoling Yang, Victor Montenegro, and Abolfazl Bayat, "Overcoming quantum metrology singularity through sequential measurements," Physical Review Letters **135**, 010401 (2025).

[4] Priyanka Sharma, Stefano Olivares, Devendra Kumar Mishra, and Matteo GA Paris, "Mitigating sloppiness in joint estimation of successive squeezing parameters," New Journal of Physics **27**, 104511 (2025).

[5] Chiranjib Mukhopadhyay, Abolfazl Bayat, Victor Montenegro, and Matteo GA Paris, "Beating joint quantum estimation limits with stepwise multiparameter metrology," arXiv preprint arXiv:2506.06075 (2025).

[6] Mylenne Manrique, Marco Barbieri, Assunta Di Vizio, Miranda Parisi, Gabriele Bizzarri, Ilaria Gianani, and Matteo GA Paris, "Bayesian stepwise estimation of qubit rotations," arXiv preprint arXiv:2512.04898 (2025).

[7] George Mihailescu, Saubhik Sarkar, Abolfazl Bayat, Steve Campbell, and Andrew K Mitchell, "Metrological symmetries in singular quantum multi-parameter estimation," Quantum Science and Technology **11**, 015006 (2026).

[8] James O Berger, José M Bernardo, and Dongchu Sun, "The formal definition of reference priors," (2009).
