# Supplementary material for: Multiparameter quantum-enhanced adaptive metrology with squeezed light
Source: Nat Commun. 2026 May 14;17:6418. doi: 10.1038/s41467-026-73069-1 (PMC13376632; doi:10.1038/s41467-026-73069-1)
Supplement: Supplementary file 2 — Transparent Peer Review file [file 41467_2026_73069_MOESM2_ESM.pdf]

nature portfolio

Peer Review File

Multiparameter quantum-enhanced adaptive metrology with squeezed light

Corresponding Author: Dr Valeria Cimini

This file contains all reviewer reports in order by version, followed by all author rebuttals in order by version.

Version 0:

Reviewer comments:

Reviewer #1

(Remarks to the Author)
The manuscript reports on an experiment that uses squeezed vacuum states of light for ab-initio phase estimation. The phase to be determined is assumed to be completely unknown. In order to adapt the squeezed vacuum states to this, real-time feedback is implemented to jointly estimate both the squeezing phase and the squeezing level.
The experiment conducted thus concerns the other extreme case of phase measurement, which is complementary to the case of laser interferometers (with squeezed vacuum states) such as gravitational wave detectors. In laser interferometers, it is not a phase that is tracked, but rather a small excitation of the well-defined phase quadrature amplitude that is measured. Since the present work does not result in a potential improvement of laser interferometers (with squeezed vacuum states), it must define even more precisely the potential progress that could result for quantum technologies. My main criticism is thus my first point.
1) The motivation for the work in the abstract refers to „strategies that rely on pre-calibrated squeezing levels“. It is unclear what strategies are meant. Later in the text, the manuscript provides references [24] and [34]. In neither of these papers did I find any indication that a pre-calibrated squeeze value is necessary. In [34], it states that there is an optimal squeezing factor in their phase tracking experiment. If it is suboptimal, only the phase tracking is less sensitive. But the method does not depend on knowledge of the value. In [24], I found no indication that their phase estimation relies on pre-calibrated squeezing levels.
GW detectors do not require a pre-calibrated squeezing level. If the squeezing factor and squeezing phase (angle) are not close to optimal, the GWs are measured with a slightly poorer S/N ratio. Active control loops ensure that this does not happen. These control loops are completely independent of whether a gravitational wave signal is currently being measured or not. Real-time measurement-based feedback is therefore not helpful.
My main criticism is therefore that the usefulness of the available results needs to be presented in a better and more concrete way. The manuscript may be suitable for publication in Nature Communications if this point is addressed sufficiently.
The following changes are also necessary prior to publication.

2) The manuscript uses two different definitions of the term “standard quantum limit.” The original and definitely preferred definition goes back to Braginski. According to this definition, the standard quantum limit is only exceeded if quantum measurement noise and quantum backaction noise are at their semiclassical minimum value and are equal in magnitude, and both are simultaneously reduced by quantum correlations. The present work does indeed refer to this definition when it cites reference [22]. (However, in [22], the success is obscured by technical noise.) [21] should not be cited here because it is a characterization prior to installation in Virgo. On the other hand, there are two theoretical papers, which refer to sub-SQL provided by squeezed vacuum states: [Unruh W. G., Quantum noise in the interferometer detector. In: Meystre P. Scully M. O. (Eds.) Quantum Optics Experimental Gravitation and Measurement Theory. Plenum pp. 647 (1983)] [Jaekel, M. T., Reynaud, S., Quantum Limits in Interferometric Measurements. Europhysics Letters 13, 301 (1990)].
In all other places in the manuscript, especially in the abstract, the term “standard quantum limit (SQL)” must be replaced by “photon shot noise”! (Unless the authors can explain why they do not mean “photon shot noise”)

3) Change line 4 of the introduction from “…in the next-generation gravitational wave detectors [2–4].” To “…in the current gravitational wave detectors [2–4].”

4) Fix typos in Ref. [3,4]

5) Add to [2–4] the new Ref. [Abbott, R. et al. GWTC-3: Compact binary coalescences observed by LIGO and Virgo during the second part of the third observing run. Phys. Rev. X 13, 041039. https://doi.org/10.1103/PhysRevX.13.041039 (2023)], where all observed GWs were measured with squeezed photon shot noise.

6) Change line 9 of the introduction from “permitted by quantum mechanics [11, 12].” To “permitted by quantum mechanics for a given photon number [11, 12].”

7) Refs. [16, 17] use “multi-pass strategies.” The idea that multi-pass can provide an advantage is obvious and very old. For this reason, gravitational wave detectors have used not only multiple passes (GEO600) but even arm resonators (LIGO, KAGRA, and Virgo) from the very beginning in the 1990s. A quantum mechanical advantage cannot be achieved with multi-pass unless something is defined incorrectly. The fundamental measurement process always involves the phase difference at the beam splitter. It does not matter whether the phase difference was collected after several passes. The authors of [16,17] have made a mistake here. The papers should under no circumstances cited with respect to “multi-pass strategies”.

Reviewer #2

(Remarks to the Author)
This manuscript aims to demonstrate an adaptive protocol that simultaneously estimates the phase and relevant nuisance parameters (squeezing and losses), in a way that is robust against calibration errors and experimental drifts, and therefore more suitable for realistic quantum metrology scenarios. Particularly, the authors implement a real-time feedback protocol that jointly estimates the optical phase and the squeezing level, using homodyne detection combined with Bayesian inference. The local oscillator (LO) phase is adapted on the fly based on previous measurement outcomes, steering the measurement towards the quadrature of highest sensitivity at each step.

Within this framework, the authors claim to: (i) achieve sub–standard quantum limit (SQL) precision across the full periodicity interval \phi \in [0,\pi];
(ii) directly infer both the phase \phi and the squeezing strength r from the measurement record, without prior calibration; and (iii) maintain quantum-enhanced performance even when the detection efficiency \eta is also treated as an unknown parameter. Precision is quantified via the covariance matrix of the estimators, and compared with (quantum) Cramér–Rao bounds (QCRB).

Overall, I consider this a significant contribution to the field of quantum metrology, which may fit well in Nat. Comm. However, I have some comments that should be addressed before publication:

1. Notation for random variables and estimators:

The statistical notation could be made more standard and precise:

+ At present, the measurement record is introduced as (\mathbf{X} = {x_1,\dots,x_M}). In standard statistical notation, uppercase letters are usually reserved for random variables, while lowercase letters denote specific realizations (observed samples). I suggest the authors denote the random variables by uppercase and the observed dataset by lowercase (e.g., X_i vs x_i).

+ When discussing the variance/covariance of the estimators, the authors write \mathrm{Var}[\phi] and \Sigma[\vec{y}]. If the intention is to refer to the variance of the estimator (\hat{\phi}) or the covariance of (\hat{\vec{y}} = \hat{\phi}, \hat{r}), it would be clearer to use \mathrm{Var}_{\phi}[\hat{\phi}] and \Sigma_{\vec{y}}[\hat{\vec{y}}], thereby distinguishing the estimator from the true parameter value. In the context of the CRB, the variance is defined for random variables, not for the parameter itself.

2. In the description of the Bayesian estimation strategy, the authors consider a sequence of homodyne outcomes (\mathbf{X} = {x_1,\dots,x_M}) and update their knowledge of the parameters via Bayes’ theorem, starting from flat priors on \phi\in [0,\pi) and r\in [0,3]. Later in the manuscript, the Fisher information (FI) is explicitly shown to depend on (\phi,r).

Because the FI is parameter-dependent, a flat prior in (\phi,r) space is a

subjective modelling choice that depends on the parametrization. I would encourage the authors to:

+ Comment explicitly on the role and robustness of the chosen priors, at least qualitatively.
+ Discuss whether alternative priors, such as Jeffreys priors, reference priors or matching priors could be appropriate in this setting, particularly given that r effectively plays the role of a nuisance parameter in the estimation of \phi.

3. In the final part of the work, the authors extend the protocol to the case where the overall detection efficiency \eta is also unknown. They propose a hybrid approach where \phi and r are treated within the Bayesian framework, while \eta is updated using a maximum-likelihood (ML) step: in the three-dimensional posterior, \eta is set to the maximizer of the \eta-posterior at the previous step.

This is a central part of the "fully calibration-free" claim, but the rationale and the implications of this mixed treatment are not entirely clear from the current presentation. I would encourage the authors to expand and clarify this point. In particular, it would be useful to explain why \eta is treated differently from \phi and r. Since r and \phi also correspond to non-compatible parameters, yet are handled entirely within a Bayesian framework, it would be helpful if the authors could clarify in what sense the "incompatibility" associated with \eta is special, and why this motivates an ML step rather than a full Bayesian treatment for \eta as well.

4. In the single-parameter benchmarking (known r and \eta), the authors show variances below the classical bound, close to the squeezed-state QCRB, but they also remark that the calibration required to know r and \eta employs a significant amount of resources that are usually not included in the resource budget.

Given that a central message of the paper is that calibration steps can be removed in the new protocol, I suggest:

+ Explicitly discussing how resources are counted.

+ Clarifying, at least qualitatively, how including calibration shots would affect the effective sensitivity in more conventional schemes, versus the proposed fully adaptive, calibration-free protocol.

5. In the multiparameter case, the authors state that the FI matrix becomes singular, making the simultaneous estimation of \phi and r impossible in the absence of prior information, whereas measuring at multiple homodyne settings restores a non-singular FI matrix. This is an interesting and important point, but it is presented rather briefly. I suggest that the authors provide a citation if this is a known general result.

Version 1:

Reviewer comments:

Reviewer #1

(Remarks to the Author)
The authors have taken my comments from the first review into account in the new version of the manuscript. The new manuscript no longer gives the impression that it describes a method that could be used in gravitational wave detectors. For, as I understand it, that would certainly not be the case.
I support the publication of the revised version in Nature Communications.

Reviewer #2

(Remarks to the Author)
I have reviewed the revised version of the manuscript, as well as the authors' responses to the comments raised by the reviewers. I believe the authors have made a good effort to address the referees' comments and to clarify the previously

misleading parts of the paper. In particular, the concerns I raised regarding the manuscript have been satisfactorily addressed. In view of these improvements, I recommend that the paper be accepted for publication.

Reviewer #4

(Remarks to the Author)
I co-reviewed this manuscript with one of the reviewers who provided the listed reports. This is part of the Nature Communications initiative to facilitate training in peer review and to provide appropriate recognition for Early Career Researchers who co-review manuscripts.

Open Access This Peer Review File is licensed under a Creative Commons Attribution 4.0 International License, which permits use, sharing, adaptation, distribution and reproduction in any medium or format, as long as you give appropriate credit to the original author(s) and the source, provide a link to the Creative Commons license, and indicate if changes were made.
In cases where reviewers are anonymous, credit should be given to 'Anonymous Referee' and the source.
The images or other third party material in this Peer Review File are included in the article’s Creative Commons license, unless indicated otherwise in a credit line to the material. If material is not included in the article’s Creative Commons license and your intended use is not permitted by statutory regulation or exceeds the permitted use, you will need to obtain permission directly from the copyright holder.
To view a copy of this license, visit https://creativecommons.org/licenses/by/4.0/

Response to the Reviewers' comments

We thank all Reviewers for their careful reading of our manuscript and for their constructive comments and suggestions. In what follows, we quote relevant parts of their reports in boldface, while our replies are in standard Roman font. Any addition/change to the text of the manuscript is reported here in blue.

Reply to Reviewer 1

The experiment conducted thus concerns the other extreme case of phase measurement, which is complementary to the case of laser interferometers (with squeezed vacuum states) such as gravitational wave detectors. In laser interferometers, it is not a phase that is tracked, but rather a small excitation of the well-defined phase quadrature amplitude that is measured. Since the present work does not result in a potential improvement of laser interferometers (with squeezed vacuum states), it must define even more precisely the potential progress that could result for quantum technologies. My main criticism is thus my first point. 1) The motivation for the work in the abstract refers to "strategies that rely on pre-calibrated squeezing levels". It is unclear what strategies are meant. Later in the text, the manuscript provides references [24] and [34]. In neither of these papers did I find any indication that a pre-calibrated squeeze value is necessary. In [34], it states that there is an optimal squeezing factor in their phase tracking experiment. If it is suboptimal, only the phase tracking is less sensitive. But the method does not depend on knowledge of the value. In [24], I found no indication that their phase estimation relies on pre-calibrated squeezing levels. GW detectors do not require a pre-calibrated squeezing level. If the squeezing factor and squeezing phase (angle) are not close to optimal, the GWs are measured with a slightly poorer S/N ratio. Active control loops ensure that this does not happen. These control loops are completely independent of whether a gravitational wave signal is currently being measured or not. Real-time measurement-based feedback is therefore not helpful. My main criticism is therefore that the usefulness of the available results needs to be presented in a better and more concrete way. The manuscript may be suitable for publication in Nature Communications if this point is addressed sufficiently.

We thank the Reviewer for their insightful comments regarding the distinction between GW interferometry and our work on ab-initio phase estimation. We fully agree that our experiment targets a different operational regime from large-scale laser interferometers: our goal is ab-initio optical phase estimation over the full interval $[0, \pi)$, rather than detecting a small excitation around a fixed working point. We have therefore revised the manuscript to avoid suggesting that our protocol provides a direct improvement for GW interferometers, and we now present GW detectors only as a motivating example of the broader impact of squeezed light.

On the contrary, concerning references [24] (Berni et al. Ref. [26] in the revised version) and [34] (Yonezawa et al., Ref. [36] in the revised version), both rely fundamentally on prior parameter knowledge for their estimation algorithms. This is precisely the calibration overhead we remove by learning the relevant nuisance parameters from the same data stream used for phase estimation, enabling operation without external recalibration stages and increasing robustness against drift.

In Ref.[24], the adaptive Bayesian estimator is explicitly model-based, the core of their estimation algorithm relies on the posterior update that requires the likelihood (given in Eq.3 of [24]), which is a Gaussian defined by the probe state variance:

$$\sigma_\phi^2 = (2n_{th} + 1)[e^{-2r}\cos^2(\phi) + e^{2r}\sin^2(\phi)], \tag{1}$$

which is a function of r and the thermal noise n_{th}. Furthermore, also the local oscillator feedback phase relies on the knowledge of the adaptive phase $\phi_{\text{opt}}^{\text{th}} = \arccos(\tanh(2r))/2$. Without knowing r,

the controller cannot identify the setpoint for maximum Fisher Information. In their Supplementary Information the authors indeed write that estimating r and n_{th} is needed "beforehand" to compute the optimal phase and the likelihood, and they repeat parameter estimation before each test because changes in squeezing change the optimal phase and must be taken into account in feedback calibration. Finally, to manage real-time processing, [24] utilized Look-Up Tables (LUTs) in an FPGA. The authors explicitly note in the Supplementary Material that these LUTs were generated using a proper calibration of system parameters and that new LUTs were required before each experimental test to address variations in squeezing strength.

Our method removes exactly this requirement by learning r and also η online, thus making the whole procedure robust to drifts and noise. Indeed, these other approaches relies on the specific pre-calibration stage, and any drift of additional noise will result in degrading the performance or in the need to repeat the pre-calibration stage.

Concerning Ref.[34], while the Reviewer correctly notes that suboptimal squeezing reduces sensitivity, the key point is that in their real-time tracking, the algorithm uses the Kalman filter whose optimal gain is an explicit function of the effective squeezing $\overline{R}_{sq}$. In the main text, they state that the squeezing and anti-squeezing levels, necessary to obtain $\overline{R}_{sq}$, were measured "independently of the phase estimation" for each pump power. This is a clear instance of pre-characterizing the probe noise parameters to validate and operate the protocol near the optimum. In the Supplementary Information, they calculate the total homodyne efficiency from visibility, optical transmission, photodiode quantum efficiency, and electronics noise, and they use this in modeling the observed squeezing/anti-squeezing and loss. This is exactly the kind of pre-characterization of the detection chain that we refer to.

Our contribution is therefore not that previous protocols do not work, but that they operate under a calibrated-model assumption: needed to compute the likelihood and the tuning of the adaptive/optimal estimator, which requires externally supplied values of key nuisance parameters. In practice, these parameters have to be re-characterized when conditions change. We demonstrate experimentally a parameter-free ab-initio approach that avoids the exhaustive pre-characterization steps described in both [24] and [34]. This is a significant progress for operation in dynamic environments where squeezing levels may fluctuate or where offline calibration is not feasible.

Such conditions are common in application-driven platforms already using squeezed light such as quantum-enhanced biosensing and plasmonic sensing (where the sample modifies absorption/scattering and thus changes η and the detected noise statistics during long acquisitions), broadband spectroscopy and nonlinear microscopy, and in continuous variable optical links and distributed sensing (where phase recovery depends on calibrated receiver models and link efficiencies vary in time and scale with network size). In these fields, frequent offline recalibration is both operationally costly and intrinsically unable to capture drifts in real-time. Maintaining quantum enhancement, therefore, requires keeping the inference model and measurement settings aligned with the instantaneous noise conditions.

To make the practical context explicit (and to address the Reviewer's request for clearer motivation outside GW interferometry), we have extended the Introduction to explicitly frame our protocol as a self-calibrating adaptive strategy for precisely these non-stationary scenarios, where static noise models become unreliable and online estimation of the nuisance parameters is essential.

> *The necessity of pre-calibration is not merely a laboratory inconvenience but a fundamental bottleneck for the deployment of quantum sensors in complex, non-stationary environments. ~~Realistic sensing scenarios introduce an additional layer of complexity.~~ In many cases, not only is the sample-induced phase shift unknown, but properties of the probe state itself, such as the squeezing strength, may also be subject to variations during the measurement time [37-39]. This is particularly evident in fields such as quantum-enhanced biosensing and plasmonics, ranging from the study of protein adsorption kinetics [40-41], to the monitoring of bacterial growth with squeezed light [42]. In these settings, the interaction between the probe and the biological sample manifests as a change in transmitted or reflected intensity, which intrinsically modifies the detection efficiency and noise statistics in real-time. Similarly, in broadband spectroscopy [43,44] and nonlinear microscopy [45], factors such as varying mode overlap, dynamic loss budgeting, and phase-lock stability fluctuate between experimental runs, translating into drifting nuisance parameters that invalidate static noise*

models. Conventional protocols typically address these instabilities through frequent recalibration cycles, which consume measurement time and fail to track rapid environmental drifts. Optimal operation in these regimes, therefore, requires a strategy capable of tracking a time-dependent signal while simultaneously inferring the nuisance parameters that define the inference model itself. Addressing such situations requires a multiparameter estimation framework [46,47], in which both phase and probe parameters are inferred simultaneously. Since the corresponding observables are generally non-commuting, quantum incompatibility imposes trade-offs on precision [47-50], making it essential to investigate experimentally how adaptive protocols perform in this regime [51]. To this end, a stepwise estimation strategy has recently been introduced [52-54], alongside several other methods aimed at boosting precision in this framework [55-58].

This article reports the experimental realization of quantum-enhanced optical phase estimation over the full π-range [59], marking a decisive step toward practical multiparameter estimation with squeezed light. Importantly, beyond interferometric phase sensing, our framework maps directly onto a broad class of practical quantum-enhanced sensors that rely on bright optical probes and intensity readout, where intensity fluctuations provide an effective measurement of the amplitude quadrature, so the achievable sensitivity is jointly determined by drifting probe and channel parameters such as losses. Our multiparameter strategy therefore treats phase, squeezing, and efficiency as co-estimated parameters, enabling self-calibrated quantum enhancement under realistic drift.

The following changes are also necessary prior to publication. 2) The manuscript uses two different definitions of the term "standard quantum limit." The original and definitely preferred definition goes back to Braginski. According to this definition, the standard quantum limit is only exceeded if quantum measurement noise and quantum backaction noise are at their semiclassical minimum value and are equal in magnitude, and both are simultaneously reduced by quantum correlations. The present work does indeed refer to this definition when it cites reference [22]. (However, in [22], the success is obscured by technical noise.) [21] should not be cited here because it is a characterization prior to installation in Virgo. On the other hand, there are two theoretical papers, which refer to sub-SQL provided by squeezed vacuum states: [Unruh W. G., Quantum noise in the interferometer detector. In: Meystre P. Scully M. O. (Eds.) Quantum Optics Experimental Gravitation and Measurement Theory. Plenum pp. 647 (1983)] [Jaekel, M. T., Reynaud, S., Quantum Limits in Interferometric Measurements. Europhysics Letters 13, 301 (1990)]. In all other places in the manuscript, especially in the abstract, the term "standard quantum limit (SQL)" must be replaced by "photon shot noise"! (Unless the authors can explain why they do not mean "photon shot noise")

Indeed, there is a misalignment between the quantum metrology and GW-interferometry communities concerning the use of the term standard quantum limit (SQL). In our work, the classical benchmark we compare against is the coherent-state Quantum Cramér–Rao bound (QCRB) for phase estimation at fixed mean photon number, i.e., $\mathrm{Var}_{\mathrm{coh}}[\phi] = \frac{1}{4\langle n\rangle}$, which corresponds to the photon shot-noise limit where $\Delta\phi \propto 1/\sqrt{\langle n\rangle}$. In a substantial part of the quantum-metrology literature, this $\frac{1}{\sqrt{N}}$ scaling for uncorrelated (classical) probes is explicitly referred to as the SQL, and it is often used interchangeably with "shot noise limit". Representative examples include widely cited papers and important review for quantum metrology such as, but not limited to, [Nature Photon. 5, 222–229 (2011) - Nature Physics 7, 406–411 (2011) - AVS Quantum Sci. 2, 024703 (2020) - Rev. Mod. Phys. 90, 035005 (2018) - Nature Comm. 6, 6726 (2015)].

By contrast, in the GW-interferometry context, SQL has a more specific meaning originating from Braginsky, as the Reviewer correctly points out. It denotes the sensitivity limit that results from the trade-off between measurement imprecision (shot noise) and quantum backaction (radiation-pressure noise), and surpassing it requires quantum correlations that reshape both contributions. However, such a definition is not the relevant benchmark for our tabletop phase-estimation setting, where radiation-pressure backaction is negligible and the sensitivity is set by optical shot noise and probe statistic. We agree that using SQL without qualification can be ambiguous, especially when discussing GW interferometers. To eliminate any possibility of confusion, we have revised the manuscript to refer consistently to the coherent benchmark as the "shot-noise limit (coherent-state bound)". In addition,

following the Reviewer's suggestion, we removed Ref. [21] from the sentence where it was not the most appropriate support and replaced it with the theoretical works suggested, which explicitly discuss sub-SQL operation in the Braginsky sense.

3) Change line 4 of the introduction from "... in the next-generation gravitational wave detectors [2–4]." To "... in the current gravitational wave detectors [2–4]." 4) Fix typos in Ref. [3,4] 5) Add to [2–4] the new Ref. [Abbott, R. et al. GWTC-3: Compact binary coalescences observed by LIGO and Virgo during the second part of the third observing run. Phys. Rev. X 13, 041039. https://doi.org/10.1103/PhysRevX.13.041039 (2023)], where all observed GWs were measured with squeezed photon shot noise. 6) Change line 9 of the introduction from "permitted by quantum mechanics [11, 12]." To "permitted by quantum mechanics for a given photon number [11, 12]."

We have followed all the Reviewer suggestions and revised the manuscript accordingly.

7) Refs. [16, 17] use "multi-pass strategies." The idea that multi-pass can provide an advantage is obvious and very old. For this reason, gravitational wave detectors have used not only multiple passes (GEO600) but even arm resonators (LIGO, KAGRA, and Virgo) from the very beginning in the 1990s. A quantum mechanical advantage cannot be achieved with multi-pass unless something is defined incorrectly. The fundamental measurement process always involves the phase difference at the beam splitter. It does not matter whether the phase difference was collected after several passes. The authors of [16,17] have made a mistake here. The papers should under no circumstances cited with respect to "multi-pass strategies".

Our intention in citing Refs. [16,17] was not to claim that multi-pass interferometry can beat quantum limits in the sense relevant to gravitational-wave detectors, nor to suggest that the fundamental readout observable is anything other than the phase difference at the beam splitter.

Rather, Refs. [16,17] are commonly cited in the quantum-metrology literature as examples of sequential (multi-pass) interrogation, where the phase shift is coherently applied multiple times to otherwise unentangled probes and the relevant resource is the total number of phase applications (photon-passes). Under that resource counting, these protocols do not violate any fundamental limit; they are effectively equivalent (in scaling) to parallel/entangled strategies discussed in the general quantum-metrology framework.

To avoid any misunderstanding we have now rewritten the paragraph as follows:

> *Different routes have been explored, such as multi-pass strategies, where the same phase shift is coherently sampled multiple times [17,18] and photon total angular momentum encoding [19]. In contrast, ~~but~~ squeezed light provides a more scalable route to phase estimation beyond the standard quantum limit (SQL) [20,21], where the advantage arises from nonclassical fluctuations at fixed mean photon number, rather than by increasing the effective interaction through repeated sampling, while mitigating some of the key limitations associated with maximally entangled probes.*

Reply to Reviewer 2

Overall, I consider this a significant contribution to the field of quantum metrology, which may fit well in Nat. Comm. However, I have some comments that should be addressed before publication:

1) Notation for random variables and estimators:
The statistical notation could be made more standard and precise:
+ **At present, the measurement record is introduced as ($\mathbf{X} = x_1, \ldots, x_M$). In standard statistical notation, uppercase letters are usually reserved for random variables, while lowercase letters denote specific realizations (observed samples). I suggest the authors denote the random variables by uppercase and the observed dataset by lowercase (e.g., X_i vs x_i).**
+ **When discussing the variance/covariance of the estimators, the authors write $\mathrm{Var}[\phi]$ and $\Sigma[\vec{y}]$. If the intention is to refer to the variance of the estimator ($\hat{\phi}$) or the covariance of ($\hat{\vec{y}} = \hat{\phi}, \hat{r}$), it would be clearer to use $\mathrm{Var}_\phi[\hat{\phi}]$ and $\Sigma_{\vec{y}}[\hat{\vec{y}}]$, thereby distinguishing the estimator from the true parameter value. In the context of the CRB, the variance is defined for random variables, not for the parameter itself.**

We thank the Reviewer for their suggestions regarding the notation. We have implemented the requested modifications and standardized the notation throughout the manuscript.

2) In the description of the Bayesian estimation strategy, the authors consider a sequence of homodyne outcomes ($\mathbf{X} = x_1, \ldots, x_M$) and update their knowledge of the parameters via Bayes' theorem, starting from flat priors on $\phi \in [0, \pi)$ and $r \in [0, 3]$. Later in the manuscript, the Fisher information (FI) is explicitly shown to depend on (ϕ, r).
Because the FI is parameter-dependent, a flat prior in (ϕ, r) space is a subjective modelling choice that depends on the parametrization. I would encourage the authors to:
+ **Comment explicitly on the role and robustness of the chosen priors, at least qualitatively.**
+ **Discuss whether alternative priors, such as Jeffreys priors, reference priors or matching priors could be appropriate in this setting, particularly given that r effectively plays the role of a nuisance parameter in the estimation of ϕ.**

In our implementation, the use of uniform priors on $\phi \in [0, \pi)$ and $r \in [0, 3]$ is primarily an initialization that reflects the ab-initio scenario we target: it encodes no preference over the phase and only a broad physically allowed range for the squeezing level, without assuming any pre-calibration. To demonstrate this robustness, we performed simulations comparing the protocol performance on the estimate of ϕ as a function of number of resources for a set of informed prior. Furthermore, the effectiveness of the approach even for a uniform prior highlights the capability of the approach to work even in the absence of a prior calibration of the system, thus being a resource-effective approach for practical sensors.

To address the Reviewer's concern about parametrization dependence and the role of priors when the Fisher information is parameter dependent, we explicitly tested the robustness of our results under alternative prior assumptions. We performed numerical simulations of our adaptive Bayesian protocol using a range of initial distributions, from the original flat priors to highly informed Gaussian priors centered on the true values $(\phi_{\text{true}}, r_{\text{true}})$ with varying standard deviation σ.

In the limit of sufficiently large M, where the Cramér-Rao-Bound (CRB) becomes meaningful, the posterior distribution becomes sharply peaked and the final phase variance is dominated by the likelihood rather than the prior. Our simulations show, as expected, that while a strongly informative prior (small σ) reduces the number of samples M required to reach the CRB, the final estimation variance at the end of the adaptive run is identical for all prior choices. This confirms that the quantum enhancement reported in our work is a property of the measurement data and the adaptive feedback logic, rather than an artifact of the prior modeling.

We further addressed the Reviewer's specific suggestion regarding objective priors and the handling of r as a nuisance parameter. In our multiparameter framework, for a single homodyne measurement

setting, the Fisher Information Matrix (FIM) is singular. For this reason, we have to consider different measurement settings (as discussed in SI section III B). In line with this, we evaluated the performance using a reference prior approach where we prioritized the marginal information for ϕ, effectively treating r as a nuisance parameter. Calculating the prior based on the square root of the diagonal elements of the FIM i.e., $F_{\phi,\phi}$ and $F_{r,r}$. However both these cases revealed no statistically significant difference in the final phase estimate compared to our original uniform prior.

This confirms that the information gain from the implemented protocol rapidly washes out the initial prior distribution.

We include the following study in the revised SI section IV.

> *Finally, we study the robustness of the implemented protocol with respect to the initial prior choice. In Fig.1 we report the simulated phase estimation variance as a function of processed homodyne samples, when adopting different priors from the flat prior used in the experimental realization. We study the algorithm performance when starting from Gaussian informed priors centred on the true values (ϕ_{true}, r_{true}) with different widths, and for Jeffrey's based on Fisher-information weighting priors[8]. As is clear from the simulated results, the performance after a sufficiently large number of M becomes independent of the particular choice of the prior.*

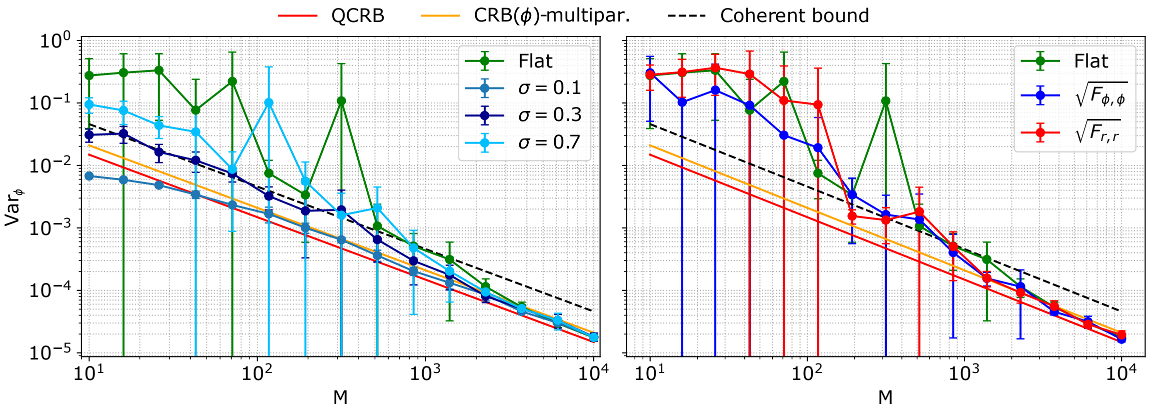

FIG. 1. **Robustness of phase estimation sensitivity to prior choice.** Numerical simulation results for the phase estimation variance as a function of the number of homodyne samples M, comparing the flat prior used in the experiment (green) against alternative initial distributions. The left panel shows the protocol performance when adopting Gaussian informative priors with different widths (σ) and centered on the true parameters, $p(\phi, r) = \mathcal{N}(\phi_{\text{true}}, r_{\text{true}}, \sigma)$. The right panel reports the results for independent Jeffreys priors, $p(\phi, r) \propto \sqrt{F_{\phi,\phi}}$ and $p(\phi, r) \propto \sqrt{F_{r,r}}$. In all cases, the adaptive Bayesian update ensures that the estimator is dominated by the measurement data at large M, with the phase variance converging to the same quantum-enhanced limit regardless of the initial prior modeling.

3) In the final part of the work, the authors extend the protocol to the case where the overall detection efficiency η is also unknown. They propose a hybrid approach where ϕ and r are treated within the Bayesian framework, while η is updated using a maximum-likelihood (ML) step: in the three-dimensional posterior, η is set to the maximizer of the η-posterior at the previous step. This is a central part of the "fully calibration-free" claim, but the rationale and the implications of this mixed treatment are not entirely clear from the current presentation. I would encourage the authors to expand and clarify this point. In particular, it would be useful to explain why η is treated differently from ϕ and r. Since r and ϕ also correspond to non-compatible parameters, yet are handled entirely within a Bayesian framework, it would be helpful if the authors could clarify in what sense the "incompatibility" associated with η is special, and why this motivates an ML step rather than a full Bayesian treatment for η as well.

We thank the Reviewer for the question that allows us to clarify the reasons behind our choice. This choice is not strictly related to the incompatibility among the parameters, but rather, here the key point is that the three parameters play different roles in the experiment and in the online inference loop. In our setting, ϕ is the signal parameter to be tracked/estimated in real time, and r is an effective probe parameter that can drift over experimental timescales, so propagating their full uncertainty is essential because the adaptive measurement setting and the posterior updates depend sensitively on them. By contrast, η is the overall detection efficiency of the experimental setup; over a single run it is typically static or slowly varying compared to the other two parameters. In sequential inference, it is standard practice to treat such "static" nuisance parameters differently from the parameters that must be tracked at the measurement rate. From an algorithmic standpoint, carrying η as an additional fully Bayesian dimension in an online Sequential Monte Carlo (SMC) scheme is possible in principle, but it is well known (*N. Kantas, et al. "On particle methods for parameter estimation in state-space models." 328-351, (2015)*) to be numerically delicate and computationally costly: for static parameters, particle representations tend to concentrate, leading to particle degeneracy and long-term error accumulation unless one introduces more complex schemes that are particularly expensive in terms of computational resources.

For this reason, a widely used alternative in state-space and signal-processing literature is a hybrid strategy where one maintains a Bayesian filtering distribution for the quantities of interest while updating static nuisance/model parameters through Maximum Likelihood (ML) steps like in *G. Kitagawa, "A self-organizing state-space model." Journal of the American Statistical Association, 1203-1215 (1998)* or in *O. Cappé, "Online sequential monte carlo em algorithm", IEEE/SP 15th Workshop on Statistical Signal Processing, 37-40, (2009).*

We have added the following paragraph to make this distinction explicit, where we cite the above mentioned parameter-learning literature to clarify that the ML plug-in update for static parameters is a standard and well-justified procedure in online inference.

> *Estimating losses jointly with the phase is particularly challenging~~, since the corresponding parameters are incompatible observables~~ in quantum multiparameter estimation since the probe and measurement choices that optimize one parameter are generally not optimal for the other, leading to fundamental tradeoffs and, in general, preventing a single measurement strategy from simultaneously saturating the corresponding single-parameter bounds [73]. Furthermore, η is a global setup parameter expected to be static (or slowly varying) over a run; carrying a full Bayesian representation of such a static nuisance parameter in an online update substantially increases the dimensionality and can lead to degeneracies in the numerical approximation. To overcome this limitation, we adopt a hybrid strategy that combines Bayesian inference with a maximum-likelihood (ML) estimation of η, that has been demonstrated to remain stable and computationally efficient [74,75]. Specifically, during each update of the three-dimensional posterior, η is set to the maximizer of the $\eta-$posterior at the previous step and used as a plug-in value in the likelihood for the Bayesian update of (ϕ, r). This update bypasses full three-parameter inference...*

4) In the single-parameter benchmarking (known r and η), the authors show variances below the classical bound, close to the squeezed-state QCRB, but they also remark that the calibration required to know r and η employs a significant amount of resources that are usually not included in the resource budget. Given that a central message of the paper is that calibration steps can be removed in the new protocol, I suggest:
+ Explicitly discussing how resources are counted.
+ Clarifying, at least qualitatively, how including calibration shots would affect the effective sensitivity in more conventional schemes, versus the proposed fully adaptive, calibration-free protocol.

In our work, the metrological resources are the number of detected samples, i.e., the number of homodyne outcomes processed to infer the parameter of interest. This is the same convention adopted in previous adaptive demonstrations with squeezed light (see Berni et al.), where the comparison to the single-parameter QCRB is expressed in terms of the number of homodyne samples used in the phase-estimation stage. Importantly, however, in the single-parameter benchmarking scenario,

the likelihood required for Bayesian (or maximum-likelihood) inference cannot be specified without pre-calibrated values of r and η, and obtaining these parameters requires additional homodyne data acquired before the sensing run (and, in realistic conditions, potentially repeated as drifts occur). Following the Reviewer's suggestion, we have therefore added an explicit analysis showing how the precision of such a pre-calibration scales with the number of homodyne samples devoted to it. This makes quantitative the fact that the knowledge of a likelihood model accurate enough to operate close to the single-parameter QCRB typically requires a non-negligible calibration budget M_{calib}. The result is that to have a really fair comparison, this scenario should also include this resource budget in the computation of the ultimate precision bound. However, in general, the bound reported is $\frac{1}{MF_Q}$, where M is the number of samples used only for estimating ϕ. The overall resources are instead $M_{\text{tot}} = M + M_{\text{calib}}$, where M_{calib} is the number of samples consumed to determine r and η. This will consistently impact the final single parameter estimation performance. On the contrary, our multiparameter strategy does not involve any pre-calibration and the total number of used resources are alredy included in the computation of the bound. This really highlights the operational advantage of the calibration-free strategy. Because it learns the relevant nuisance parameters online from the same measurement record used to infer ϕ there is no separate calibration stage whose samples are excluded from the metrological budget. All detected samples contribute to the joint inference and to the adaptive choice of measurement setting, so the reported precision already reflects the full resource consumption, thus eliminating the structural separation between calibration and sensing that otherwise inflates the apparent performance when calibration resources are not accounted for.

To stress this important point, we have now included a new subsection in the Supplementary Information.

A. Pre-calibration measurements

In the single parameter strategy, the probe and system parameters r and η, have to be pre-calibrated. In practice, this requires collecting additional homodyne data before the phase-estimation run. This stage represents a genuine metrological cost because these extra *samples do not directly contribute to estimating the parameter of interest. To quantify the amount of required* extra *resources, we determine, from experimental measurements, the relative uncertainty on the calibration measurements of r and η as a function of the number of homodyne samples M_{calib} employed in this pre-calibration stage. The results reported in Fig.6 show that achieving a relative error, on both measurement of r and η, below 1% requires employing at least $M_{calib} = 5000$ samples. A perfect calibration is instead obtained for M_{calib} larger than $40k$. This makes explicit that, even under stable conditions, obtaining r and η values needed to retrieve the likelihood and the optimal measurement projection assumed in single-parameter benchmarking entails a non-negligible measurement cost.*

To highlight the operational consequence, we therefore re-evaluate the single-parameter quantum and classical bounds under an explicit resource accounting in which the total number of detected samples is $M_{tot} = M + M_{calib}$. As shown in Fig.3, this shifts away the experimentally evaluated variances from the ultimate QCRB computed considering M_{tot}. In particular, requiring a relative error on both r and η of the order of 1% requires an amount of calibration measurements M_{calib} that, if included in the overall resource budget when computing the bounds, washes away the quantum enhanced performances achieved in this scenario. This is shown by the fact that in this configuration the experimental points lie on the blue classical coherent bound.

By contrast, our multiparameter strategy has no separate pre-calibration stage: the relevant nuisance parameters are inferred online from the same measurement record used to estimate ϕ. In this scenario, we already include in the reported performance and in the corresponding bounds the total resource consumption. All detected samples contribute simultaneously to updating the inference model and to steering the adaptive measurement toward maximal information. This removes the structural separation between calibration and sensing that can otherwise inflate apparent performance when calibration resources are omitted, and it directly illustrates the operational advantage of a genuinely calibration-free protocol.

5) In the multiparameter case, the authors state that the FI matrix becomes singular, making the simultaneous estimation of ϕ and r impossible in the absence of prior infor-

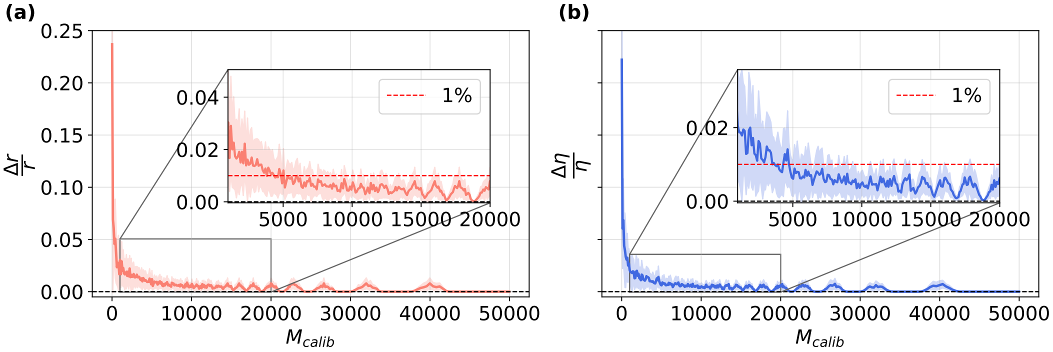

FIG. 2. **Resources for pre-calibration.** The experimental relative error of the pre-calibrated squeezing parameter r and overall efficiency eta values as a function of the number of processed homodyne samples M_{calib}, are reported in panel **(a)** and **(b)**, respectively. Insets highlight the 1% relative-error threshold (red dashed line). The black dashed line denotes the perfect estimate.

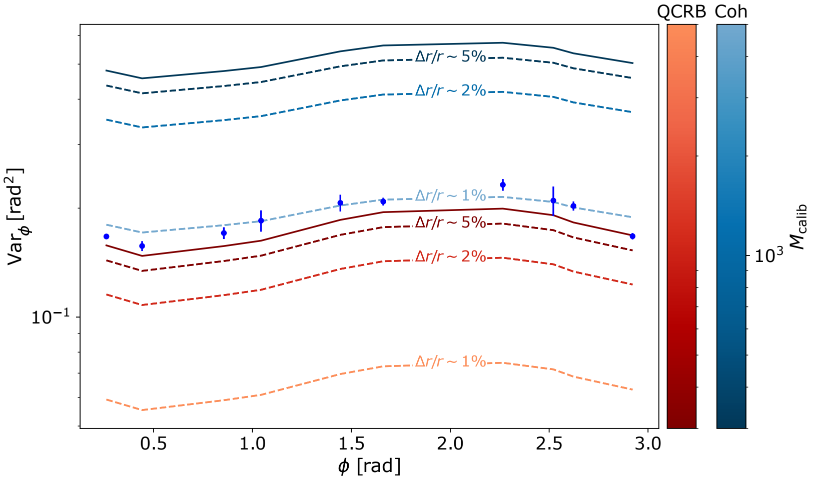

FIG. 3. **Effects of calibration resources accounting on the estimation variance bounds.** The experimental estimation variances for the single-parameter protocol are reported as blue dots for different values of the estimated phase ϕ. If the resources needed to pre-calibrate the nuisance parameters are not accounted for, the resulting QCRB and coherent bound are represented by solid blue and red lines, respectively, the same as reported in the main text. We report here as dashed lines in shades of red and blue, the bounds computed including also the resources needed for calibration. This shows how the QCRB and coherent bound change according to three different levels of relative error in the pre-calibration of the squeezing strength, i.e. $\Delta r/r \sim 5\%$, 2%, and 1%.

mation, whereas measuring at multiple homodyne settings restores a non-singular FI matrix. This is an interesting and important point, but it is presented rather briefly. I suggest that the authors provide a citation if this is a known general result.

We thank the Reviewer for highlighting this aspect, which is indeed crucial for our estimation protocol. Since the Fisher Information Matrix (FIM) singularities are a common issue in the context of multiparameter estimation, several techniques have been developed to mitigate its effects, such as the use of the Moore-Penrose pseudoinverse [Stoica, Petre et al. IEEE Transactions on Signal Processing 49.1 (2002): 87-90.], sequential measurement strategies [Yang, Yaoling et al. Physical Review Letters 135.1 (2025): 010401.], scrambling operations [Priyanka Sharma et al. 2025 New J.

Phys. 27 104511], and step-wise estimation protocols [Mukhopadhyay, Chiranjib, et al. arXiv preprint arXiv:2506.06075 (2025), Manrique, Mylenne, et al. arXiv preprint arXiv:2512.04898 (2025)]. However, to our knowledge, there are no general results addressing instances in which the FIM singularity is removed by performing consecutive measurements in different settings, as in our adaptive protocol. Ultimately, this property relies on the fact that the sum of singular matrices is not necessarily a singular matrix. When performing independent measurements, the overall FIM corresponds to the sum of the individual FIMs, therefore, in our case, consecutive measurements with different homodyne settings can remove the singularity.

In order to gain further physical understanding of this process, we have written an additional section in the Supplementary Information (also reported below) in which we exploit the framework introduced in [Mihailescu, George, et al. Quantum Science and Technology 11.1 (2026): 015006.], showing how FIM singularity can be attributed to symmetries that are lifted by consecutive measurements in different settings, thereby achieving a full-rank FIM.

I. OVERCOMING FISHER INFORMATION MATRIX SINGULARITY

The singularity of the (Quantum) Fisher Information Matrix ((Q)FIM) is a well-known limitation that can arise in multiparameter estimation problems [Goldberg, Aaron Z., et al. International Journal of Quantum Information 19.08 (2021): 2140004.], preventing the standard definition of the corresponding (Q)Cramér-Rao Bound ((Q)CRB). Several approaches have been proposed to circumvent this problem, such as the use of the Moore-Penrose pseudoinverse [Stoica, Petre et al. IEEE Transactions on Signal Processing 49.1 (2002): 87-90.], sequential measurement strategies [Yang, Yaoling et al. Physical Review Letters 135.1 (2025): 010401.], scrambling operations [Priyanka Sharma et al. 2025 New J. Phys. 27 104511], and step-wise estimation protocols [Mukhopadhyay, Chiranjib, et al. arXiv preprint arXiv:2506.06075 (2025)., Manrique, Mylenne, et al. arXiv preprint arXiv:2512.04898 (2025)]. In what follows, we will show how, in our case, the FIM singularity is removed thanks to the multiple steps prescribed by the adaptive estimation protocol.

The key property of the FIM that we exploit is its additivity for independent measurements. In our case, as illustrated in Eq.(25), the overall FIM resulting from our adaptive protocol is constituted of the sum of the contributions from the different steps of the protocol. Since the sum of singular matrices is not necessarily a singular matrix itself, by properly choosing the measurements to be performed during the rough estimation, it is possible to achieve a global non-singular matrix and, therefore, a well-defined CRB.

In order to have a better physical understanding of this process, we exploit the framework presented in [Mihailescu, George, et al. Quantum Science and Technology 11.1 (2026): 015006.] to analyze the origin of FIM singularity in our model and how it can be removed. In particular, it can be demonstrated that the FIM corresponding to the joint estimation of phase and squeezing level from measurements along a single homodyne angle always leads to a singularity. This is a consequence of the parametrization of the model, since the likelihood corresponding to the homodyne detection of squeezed vacuum state (Eq. (4) of the main text) is modeled by a zero-mean Gaussian distribution with variance depending on both the phase and squeezing level, which we recall here for practicality:

$$\sigma^2(\varphi, r, \eta) = \frac{1}{4}\left(\eta e^{-2r}\cos^2\varphi + (1-\eta)\cos^2\varphi + e^{2r}\sin^2\varphi\right), \tag{2}$$

This is indeed an instance of overparametrization, where a model with only one degree of freedom (i.e. the variance) is instead described with a larger number of parameters. For this reason, there will be different combinations of ϕ and r which will produce the same quadrature variance. In the context of Bayesian estimation, a recent work [Mihailescu, George, et al. Quantum Science and Technology 11.1 (2026): 015006.] points out that for such problems, even if one cannot give a proper estimate of the parameters, it is still possible to gain insights on the singularity affecting the estimation, as the posterior distribution will not converge to a single point in parameter space, but

rather lie along a line. The curve describing this line represents the set of parameters for which the effective single parameter describing the problem remains constant. Even if a general approach to identify such an effective parameter is not provided, in our case, it is rather intuitive to associate it with quadrature variance, since it is the only quantity that is really fixed when measuring quadrature along a single angle. For this reason, we expect that, in our case, the posterior will lie along the "isovariance curve" describing the combinations of ϕ and r for which the variance in Eq.2 is equal to the observed quadrature variance. Examples of such isovariance curves are illustrated in Fig.4a.

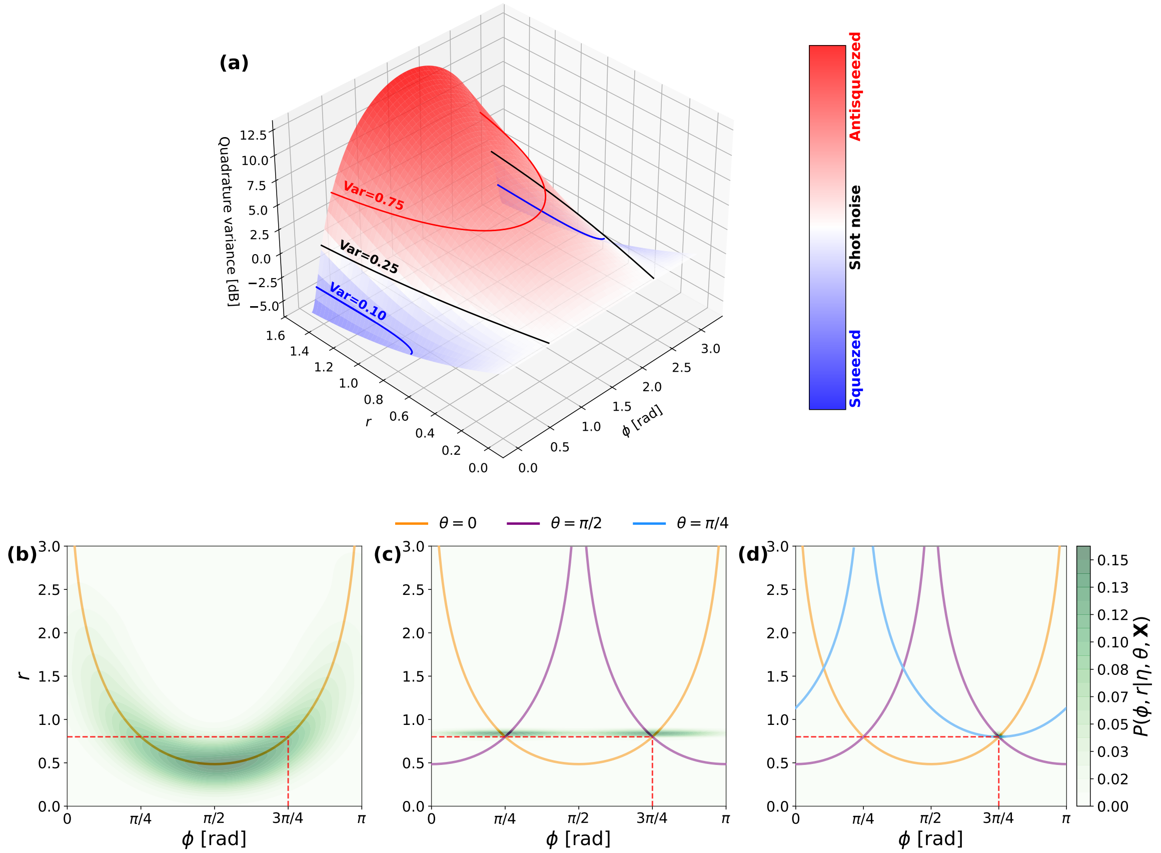

FIG. 4. **Effects of the FIM singularity on the Bayesian estimation of ϕ and r.** In panel **(a)**, we represent the behavior of the quadrature variance for the homodyne detection of a squeezed vacuum state, as a function of the phase ϕ and the squeezing strength r, while the efficiency $\eta = 0.85$ and the LO phase $\theta = 0$ are taken as fixed parameters. As examples, isovariance curves are depicted in the cases of vacuum fluctuations ($\sigma^2 = 1/4$), squeezed ($\sigma^2 = 0.1 < 1/4$), or antisqueezed ($\sigma^2 = 0.75 > 1/4$), respectively indicated by black, blue, and red solid curves. In panels **(b)**-**(d)**, we illustrate the evolution of the posterior distribution when consecutive batches of homodyne data (400 quadratures each) are acquired for different values of $\theta = 0, \pi/2, \pi/4$. For each one of these LO phases, we depict the corresponding isovariance curve (yellow, purple, and blue solid lines, respectively corresponding to $\theta = 0, \pi/2, \pi/4$), illustrating how the posterior distribution is affected by its symmetries, progressively removed as new batches of homodyne data are measured along different angles. The actual values of the parameter to be estimated are $\phi_{\text{true}} = 3\pi/4$ and $r_{\text{true}} = 0.8$, indicated by the intersection of the red dashed lines.

In order to verify this property, in Fig.4b-d, we report the behavior of the posterior distribution $p(\phi, r|\eta, \theta, \mathbf{x})$ during the rough estimation step of our protocol. In particular, we simulate 400 homodyne detection for a squeezed vacuum state with parameters $\phi_{\text{true}} = 3\pi/4$, $r_{\text{true}} = 0.8$, and $\eta = 0.85$, repeating this operation for three different LO phases $\theta = 0, \pi/2, \pi/4$. After measuring

quadratures only along $\theta = 0$*, the resulting posterior is indeed "delocalized" along the curve describing the combinations of parameters which produce a homodyne distribution with variance equal to* $\sigma^2(\phi = \phi_{\rm true}, r = r_{\rm true}, \eta)$*, as we can see in Fig. 4. Therefore, at this stage, the estimation cannot yet provide a reliable measure of* ϕ *and* r*. In Fig.4c, we can see how the singularity breaks after updating the posterior with an additional 400 homodyne measurements taken along* $\theta = \pi/2$*, since the posterior distribution is now constrained to lie in the intersection between the two isovariance curves. Even if this lifts the symmetry generating the continuous set of ambiguous pairs (*ϕ*,* r*), the estimation cannot resolve between the phases* $\phi_{\rm true}$ *and* $\pi - \phi_{\rm true}$*. The final 400 homodyne data taken with* $\theta = \pi/4$ *also remove this ambiguity, so that, as illustrated in Fig.4d, the posterior distribution concentrates around the only intersection point among the three isovariance curves, which correspond to the actual values of the parameters* $\phi_{\rm true}$ *and* $r_{\rm true}$*, therefore providing the proper estimations needed to proceed with the adaptive step of the protocol.*

Reply to Reviewers 3-4

I'm afraid I cannot recommend publication of the manuscript in Nature Communications in its current form. While certainly of interest to the metrology community, the manuscript does not make a sufficient case for a broader appeal which is required for Nature Communications. We have two major comments that we ask the authors to address
1) In the abstract it is highlighted that the authors' multiparameter strategy jointly estimates the optical phase and the squeezing level to ensure robustness against experimental drifts and calibration errors. Therefore, we would like to see a more detailed section on it. For example for the statement "Our results highlight the versatility of the adopted multiparameter approach that enables the simultaneous estimation of probe and signal parameters, while allowing the feedback strategy to be tailored to prioritize the precision of the most relevant observable (page 7, left column, upper section)" we would have liked a quantitative description of the linked precisions that are achievable for the phase and squeezing estimation. It is partially included in insets of Fig.4a), but there are no description or possible explanations for the shape change of the reconstructed posterior distributions p(phi,r|X) at different squeezing levels.

We thank the Reviewer for highlighting this aspect. We agree that a clear definition of the achievable precision in the joint estimation of multiple parameters is crucial for our protocol. Phase and squeezing strength are estimated simultaneously from the same homodyne samples, but they are incompatible quantities, meaning that it is impossible to jointly estimate them with a precision that simultaneously saturates the Quantum Cramer-Rao Bound (QCRB) for both parameters. In other words, the protocol must distribute the available information between phase estimation and probe self-calibration, which leads to a precision trade-off. A detailed discussion of this point, including the analytical framework, is given in Sec. III.A of the Supplementary Information. This trade-off is not a limitation for the goal of the present work, because our protocol is intentionally designed to prioritize phase precision, while estimating the squeezing level only to keep the inference model and feedback settings updated online. In this sense r is treated as a nuisance parameter, we aim to maximize the precision of the phase estimation at the expense of reduced precision in the squeezing level. In particular, considering the specific homodyne-based adaptive strategy that is experimentally implemented, this tradeoff explicitly emerges in the study of the corresponding Fisher Information Matrix (FIM). A quantitative study of this behavior is detailed in Sec.III.B of the Supplementary Information, where we derive the FIM for our adaptive protocol. In particular, Supplementary Figure 12 shows that the CRBs for ϕ and r reach their minima at different local oscillator phases. Consequently, when precision is optimized for phase estimation, the resulting posterior distribution becomes narrower along the phase direction and broader along the r direction. To corroborate these observations, we have extended Sec.III.B of the SI by comparing the computed CRBs with the variances experimentally obtained in the estimation of ϕ and r.
Therefore, the revised version of the SI now reads:

> *... which is detailed in the inset (c) of Fig.12. To directly connect the model to the experiment, Fig.5 compares the theoretical bounds computed in this section with the posterior variances reconstructed from the experimental data. Specifically, considering the experimentally implemented configuration that optimizes phase estimation precision, we observe that the posterior variances are compatible with the adaptive protocol CRBs for both ϕ (panel* ***(a)****) and r (panel* ***(b)****), and the phase estimation variances remain significantly below the coherent bound. In addition, in panel* ***(c)*** *we report the experimentally estimated correlation $\mathrm{Corr}[\hat{\phi}, \hat{r}] = \mathrm{Cov}[\hat{\phi}, \hat{r}]/\sqrt{\mathrm{Var}_{\phi}[\hat{\phi}]\mathrm{Var}_{r}[\hat{r}]}$ for all the measured phases, finding an overall average value of $\overline{\mathrm{Corr}}_{\phi r} = -0.085 \pm 0.037$. Such a small correlation is in agreement with the model prediction (see Fig.12b), according to which the phase-optimized operating point of the multiparameter protocol also corresponds to a near-vanishing off-diagonal term of the inverse FIM. Furthermore, panel* ***(d)*** *depicts a representative experimental posterior distribution, which directly visualizes the anisotropic uncertainty expected from the multiparameter trade-off: the posterior is more tightly localized along the phase direction (the parameter prioritized by the feedback) and broader along the squeezing direction. This provides an intuitive picture of*

how the protocol allocates information between phase estimation and probe self-calibration.

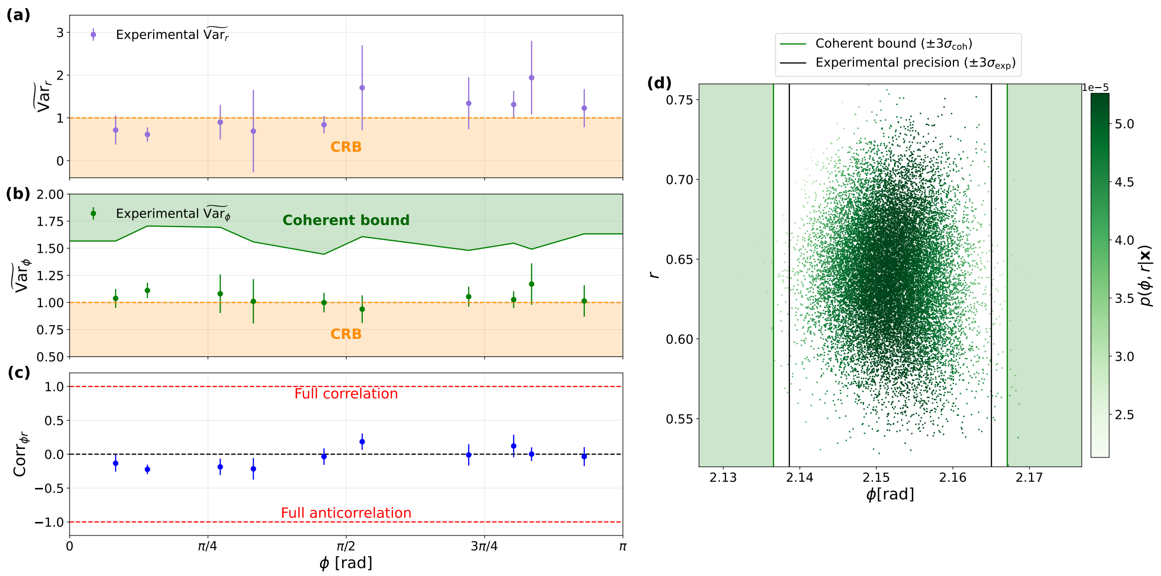

FIG. 5. **Comparison of our CRB model with experimental results.** Panels **(a)** and **(b)** display the experimental variances for the estimation of r (purple dots) and ϕ (green dots), respectively. The data are normalized to the corresponding CRB, represented by a constant dotted orange line at $\widetilde{\mathrm{Var}} = 1$, where the tilde denotes the normalization. Additionally, in panel **(b)**, the coherent bound is indicated by a solid green line. In panel **(c)**, we report the parameter correlation $\mathrm{Corr}_{\phi r} \equiv \mathrm{Cov}_{\phi r}/\sqrt{\mathrm{Var}_\phi \mathrm{Var}_r}$ obtained from the experimental multiparameter estimations, compared to the extremal values of full (anti)correlation (red dashed lines) and full lack of correlation (black dashed line). Panel **(d)** depicts a representative experimental posterior distribution (green dots) alongside the corresponding experimental standard deviation, $\sigma_{\mathrm{exp}} \equiv \sqrt{\mathrm{Var}_\phi}$, which is compared to the coherent bound standard deviation, $\sigma_{\mathrm{coh}} \equiv \sqrt{\mathrm{QCRB}_{\mathrm{coh}}}$.

2) Furthermore, the self-calibrating multiparameter approach seems like a valuable estimation strategy, but we were missing examples of specific use cases, which would immediately profit from the presented self-adaptive multiparameter phase estimation. Such examples would increase the appeal of the manuscript to a wider audience.

We thank the Reviewer for the opportunity to clarify the broader impact of our work. The need for self-calibrating multiparameter estimation a practical necessity for operating quantum-enhanced sensors in realistic scenarios where loss, alignment, and technical noise fluctuate in time.

A central point is that, in state-of-the-art quantum-enhanced sensing experiments, the inference of the parameters of interest is intrinsically model-based. The estimator requires knowledge of the likelihood (as in Bayesian squeezed-light protocols [Berni et al., Nat.Phot. 9, 577-581 (2015)] already cited in the manuscript), which usually depends not only on the sought parameter, but also on nuisance parameters describing the probe and the detection chain. For this reason, conventional experiments typically require offline characterization steps to calibrate the noise model. For long-lasting measurements, such a pre-calibration is usually repeated several times, introducing on one hand a resource overhead devoted purely to calibration rather than sensing, but even more detrimental is the fact that repeated calibrations can not track real-time drifts. Apart from estimation, the knowledge of the nuisance parameters is usually also required to identify the optimal sensor operating point used for the implementation of adaptive feedback loops.

Our work directly addresses this bottleneck. We demonstrate that high-sensitivity, ab-initio phase estimation with squeezed light can be performed without assuming pre-calibrated probe/noise parameters, by learning them online from the same measurement record used for the estimation. This removes the

need for interruptive recalibration stages and increases robustness to drift, which is essential for many application-driven deployments. Importantly, the same structure applies beyond explicit phase readout. Many practical quantum-enhanced sensors use intensity detection. This regime maps directly onto our framework because direct detection of a bright optical field is equivalent to measuring its amplitude quadrature. Also in this representation, the estimator is model-based and depends on the quadrature variance (depending on ϕ, r, and η), the only difference for those configurations is that the parameter of interest, therefore the one requiring the optimal sensitivity, is either r or η. As for our approach, the achievable sensitivity and the optimal feedback will both depend explicitly on the other two nuisance parameters. In a large class of applications, such as biosensing, absorption, spectroscopy, microscopy, and plasmonic sensors, the quantity of interest manifests indeed in a change of transmitted/reflected intensity, where losses and noise parameters can vary during long acquisitions.

In the following, we highlight representative applications where our self-calibrating strategy can directly reduce calibration overhead and improve robustness.

Quantum-enhanced biosensing and plasmonics.
In plasmonic/biosensing platforms the sample and sensor head are intrinsically lossy and can evolve during the measurement. In these platforms, to retain quantum-enhanced performance is necessary to tune the measurement configuration to maintain optimal noise cancellation. In other words, to remain at optimal sensitivity, one must continually retune based on the underlying (calibrated) noise model. A concrete example is twin-beam plasmonic sensing [Pooser and Lawrie, Optica 2, 393-399 (2015)], where the measurement configuration has to be adapted for each data point and the achievable noise reduction depends explicitly on η. Similarly, in [Dowran et al., Optica 5, 628-633 (2018)] a quantum-enhanced plasmonic refractive-index sensing experiment is carried out, requiring an empirical gain optimization to recover the best noise suppression. In both cases, the measurement remains quantum-enhanced only when the effective squeezing/correlation strength and η are known and kept near the optimum; in realistic deployable operation, these quantities drift with alignment and sample loading. The same need arises even more sharply in real-time biological sensing, where the optical response evolves throughout the measurement. In [Kamble et al., PRX Life 3, 023010, (2025)], the authors track protein–gold adsorption kinetics in real time. Here, performance hinges on a pre-characterized shot-noise reference and on the effective squeezing available after loss; they also explicitly adjust the conjugate intensity (via absorbers) to maintain optimal cancellation. Such long acquisitions are exactly where slow drifts undermine the validity of a one-time calibration. A similar aspect arises in a very recent paper [Andrade et al., arXiv:2512.12057 (2025)], which reports quantum-enhanced biosensing of bacterial growth with squeezed light. Before introducing bacteria, they characterized losses and baseline noise, and only after they perform multi-hour measurements, where slow drifts must be monitored; such baseline characterization is essential precisely because it fixes the noise model entering the estimate. This is a clear example of calibration/monitoring overhead that is required to keep the inference model valid. They further note that future developments could extend the technique to multiple parameters or spectral signatures, which makes the need for self-calibrating multiparameter inference even more pronounced. In these biosensing and plasmonic settings, our protocol maps naturally by treating the sensor's phase-like (or quadrature-displacement) response as the parameter of interest ϕ, while simultaneously learning η and the effective noise/squeezing parameter r online. This would enable uninterrupted, self-calibrated monitoring of biological kinetics and refractive-index changes, maintaining quantum advantage even as the sample and the detection chain evolve—without inserting separate "blank" recalibration steps during the measurement.

Quantum-enhanced spectroscopy and nonlinear microscopy.
Recent squeezed-comb/entangled dual-comb approaches [Belsley PRL 130, 133602, (2023); Hariri et al., PRX 15, 041009 (2025)] rely on accurate knowledge of the effective noise per channel to quantify and maintain enhancement. In quantum-enhanced nonlinear microscopy [Casacio et al., Nature 594, 201-206 (2021)], achieving sub-shot-noise operation relies on active stabilization (phase locking/overlap stability) and explicit loss-budget characterization. Performance varies between runs due to changes in overlap and phase-lock stability, necessitating calibrated homodyne models and therefore repeated characterization of the effective "prior-to-sample" squeezing. This is again the same bottleneck: the

estimator and its confidence require a correct loss budget and fit squeezed-variance data to a model before each measurement. By treating the effective squeezing at the detector as a dynamic nuisance parameter, learned from the measurement record, our approach points toward more autonomous, "plug-and-play" operation where the microscope could continuously self-calibrates its noise model based on the actual squeezing reaching the camera at that moment, rather than relying on manual per-run characterization.

Quantum communication and distributed sensing.
Optical links and continuous-variable (CV) quantum key distribution experiments [Zhang et al., PRApp. 13, 0240058 (2022), Soh et al., PRX 5, 041010, (2015)] provide another example of the same bottleneck addressed by our work. In these applications, usually the quantity of interest is a time-varying optical phase, and the security of the protocol is strongly conditioned on calibrated receiver noise and efficiency models. Quantum-enhanced phase tracking with squeezed light [Yonezawa et al., Science 337, 1514-1517 (2012)], already cited in our manuscript, makes explicit that optimal tracking depends on the probe noise properties. In such scenarios, the effective measurement noise, and thus the optimal adaptive gain / update rule, varies with loss and receiver noise. In practice, maintaining the validity of the receiver model requires dedicated calibration procedures, precisely because performance and, in QKD, security are sensitive to these parameters. Our joint estimation would allow one to keep the estimator and the adaptive update rule tuned to the actual operating conditions. The same reasoning extends naturally to distributed sensing and networked metrology in the CV regime [Guo et al., Nat.Phys. 16, 281-284 (2020)]: once multiple nodes/links are involved, nuisance parameters become heterogeneous and time-dependent, and the calibration burden scales with network size. A self-calibrating multiparameter strategy is therefore not only convenient but becomes operationally enabling, because it allows phase (or phase-combination) estimation to remain correctly modelled as link conditions drift across the network.

> *The necessity of pre-calibration is not merely a laboratory inconvenience but a fundamental bottleneck for the deployment of quantum sensors in complex, non-stationary environments. ~~Realistic sensing scenarios introduce an additional layer of complexity.~~ In many cases, not only is the sample-induced phase shift unknown, but properties of the probe state itself, such as the squeezing strength, may also be subject to variations during the measurement time [37-39]. This is particularly evident in fields such as quantum-enhanced biosensing and plasmonics, ranging from the study of protein adsorption kinetics [40-41], to the monitoring of bacterial growth with squeezed light [42]. In these settings, the interaction between the probe and the biological sample manifests as a change in transmitted or reflected intensity, which intrinsically modifies the detection efficiency and noise statistics in real-time. Similarly, in broadband spectroscopy [43,44] and nonlinear microscopy [45], factors such as varying mode overlap, dynamic loss budgeting, and phase-lock stability fluctuate between experimental runs, translating into drifting nuisance parameters that invalidate static noise models. Conventional protocols typically address these instabilities through frequent recalibration cycles, which consume measurement time and fail to track rapid environmental drifts. Optimal operation in these regimes, therefore, requires a strategy capable of tracking a time-dependent signal while simultaneously inferring the nuisance parameters that define the inference model itself. Addressing such situations requires a multiparameter estimation framework [46,47], in which both phase and probe parameters are inferred simultaneously. Since the corresponding observables are generally non-commuting, quantum incompatibility imposes trade-offs on precision [47-50], making it essential to investigate experimentally how adaptive protocols perform in this regime [51]. To this end, a stepwise estimation strategy has recently been introduced [52-54], alongside several other methods aimed at boosting precision in this framework [55-58].*
>
> *This article reports the experimental realization of quantum-enhanced optical phase estimation over the full π-range [59], marking a decisive step toward practical multiparameter estimation with squeezed light. Importantly, beyond interferometric phase sensing, our framework maps directly onto a broad class of practical quantum-enhanced sensors that rely on bright optical probes and intensity readout, where intensity fluctuations provide an effective measurement of the amplitude quadrature, so the achievable sensitivity is jointly determined by drifting probe and channel parameters such as losses. Our multiparameter strategy therefore treats phase, squeezing, and efficiency as co-estimated parameters, enabling self-calibrated quantum enhancement under realistic drift.*

Here are some additional minor comments that the authors might want to consider.
3) In Fig1 b) The depiction of the liquid crystal to introduce a phase on the squeezed state is a bit confusing. What is the purple outline, white the white dotted line? In the right plot, what are the spheres and why do they change in shape? Could one also include orange or pink dotted lines for the final state with phi_t and r_t? Similar to the blue dotted line for the first estimated phase and squeezing parameter values.

In the figure, the purple outline is intended to represent the probe field (sketch as the Wigner function of a vacuum squeezed state) whose orientation (white dashed line) depends on the phase to be inspected applied with the liquid crystal.

In the right panel, the shapes are not spheres but contour ellipses of the Bayesian posterior distribution in the (ϕ, r) parameter space. As the adaptive protocol proceeds and more homodyne outcomes are processed, the posterior becomes progressively more concentrated. The ellipses therefore shrink (reduced uncertainty) around the true values (ϕ_t, r_t). The blue ellipses indicate the three steps of the raw estimate, while the pink ones indicate the fine estimate.

To clarify this point, we have now modified the Fig.1 caption as follows:

> ***(b)*** *The probe is a squeezed state, represented by its Wigner-function ellipse (purple outline). The unknown phase shift is imprinted on the probe by the liquid-crystal element, which rotates the squeezing ellipse in phase space (denoted as the white dashed line). At each step of the protocol, the homodyne data collected are used to reconstruct the multiparameter posterior distribution. As homodyne outcomes are acquired and processed, the joint posterior becomes progressively more concentrated (represented in the sketch as the contour ellipses). Blue contours indicate the posterior after the initial raw estimation steps, while pink contours show the refined posterior during the final adaptive stage. From this posterior, the estimate is updated and the optimal measurement angle θ is computed. A feedback Δ, derived from the current estimates, is then applied to adjust the LO phase for the next measurement. Repeating this cycle progressively refines the posterior until, at the end of the protocol, the estimates converge to the true values of phase ϕ_t and of the squeezing parameter r_t.*

4) On page 2, left column, bottom section: "Optimizing the measurement strategies leads to the quantum Cramér-Rao bound (QCRB)..." is the first introduction to the QCRB. It could be potentially misleading since it sounds like by optimizing the measurement the bound becomes quantum. I would rather present the definition of the QCRB and how it is different to the CRB first and then get the explanation of how to reach that quantum bound.

In parameter estimation theory, the (classical) Cramér-Rao-Bound (CRB) is defined for a fixed measurement and is expressed in terms of the Fisher information associated with the chosen POVM. The quantum Cramér-Rao-Bound (QCRB) is obtained instead by optimizing the Fisher information over all physically allowed measurements, yielding the quantum Fisher information. In this sense, the Reviewer's interpretation of our original wording is exactly the intended meaning. We then clarified that the QCRB is obtained by optimizing over all POVMs, as shown in the paragraph below.

We also added appropriate review references that discuss these figures of merit and their relationship in detail.

> Optimizing the measurement strategy over all positive operator-valued measures (POVMs) leads to the quantum Cramér-Rao bound (QCRB)

5) Figs 2. a) and b) Is there a meaning behind the color gradient "behind" the blue and green lines. Same for the red one? Why is there only the CRB bound for the multiparameter case? To make it more consistent the CRB bound should be included for the single parameter case.

There is no meaning for the color gradient but it was only an aesthetic choice, therefore, to avoid misunderstunding we have updated the Figure in the revised version.

Concerning the second question, in the single-parameter scenario considered in our benchmark, homodyne detection with the LO phase set to the optimal working point is an optimal measurement for a squeezed Gaussian probe. Under these conditions, the Fisher information F of the implemented measurement equals the quantum Fisher information F_Q.

To avoid confusion, we add a short sentence in the text stating explicitly that in the single-parameter benchmark, the homodyne CRB overlaps with the QCRB and is therefore not shown separately.

> *In the single-parameter configuration, when the LO phase is set to the optimal working point, homodyne detection is an optimal measurement for squeezed Gaussian probes; consequently, in the lossless scenario, its FI equals the QFI and the corresponding homodyne CRB overlaps with the QCRB.*

6) Page 3, right column, middle section: "For the multiparameter protocol we also report the actual CRB (orange dashed line)." What is the actual Cramer Rao bound? Especially in comparison to the QCRB for a coherent probe.

What we meant by 'actual CRB' is the classical CRB associated to the actual (specific) measurement settings. To be more specific and avoid any confusion, we have now substituted the term *actual* with classical from the revised version.

7) Figs 3 f) and g), switch the two plots to make it consistent with e) or even plot it together.

We followed the Reviewers suggestion and updated Figure 3 in the revised manuscript.

8) p(phi|X) is first referred to as homodyne probability distribution and afterwards posterior distribution. It would help if it stayed the same or if the variable had different indices.

In the Bayesian framework, the probability distribution of a homodyne outcome x conditioned on the system parameters is the likelihood function $p(X = x|\phi, r)$, while the posterior is the distribution of the parameters conditioned on the full measurement record $\boldsymbol{x} = \{x_1, \ldots, x_M\}$ $p(\phi, r|\boldsymbol{x})$ (or its marginal $p(\phi|\boldsymbol{x})$). We double checked our manuscript to ensure to be consistent with this notation.

9) Page 6, left column, upper section: The reported gain below the SQL shows that the multiparameter strategy leads to the same result as the single parameter strategy, which needs pre-calibration. In a different perspective it also shows that the single parameter strategy is sufficient and feasible. That brings up the question of whether it is actually resource efficient to put in the work of realizing the different estimations steps plus feedback loop with the posterior estimation.

We highlight that the approach of implementing multiple estimation steps and an adaptive feedback loop is not unique to the multiparameter protocol. It is also done in the single-parameter strategy, indeed any ab-initio phase estimation protocol with squeezed light must actively steer the homodyne quadrature to remain near the region of maximal information, because the sensitivity is intrinsically phase dependent. In this sense, the different estimations steps plus feedback loop are only linked to the ab-initio/adaptive nature of the problem itself, rather than by whether implementing a single or multiparameter strategy. The difference is therefore not that the single-parameter strategy is simpler, but that it operates under a calibrated-model assumption: it requires externally supplied values of r and η to construct the estimator and to set the feedback operating point. Obtaining and maintaining those values in practice entails additional characterization measurements and periodic recalibrations that can not correct for drifts occurring during long acquisitions.

Our multiparameter strategy removes exactly this hidden overhead. It achieves the same quantum-enhanced performance while learning the relevant nuisance parameters online from the same homodyne record used for estimating ϕ. As a consequence, there is no separate calibration stage that consumes resources without contributing to the sensing task, and the protocol remains valid under realistic conditions where squeezing level, detection efficiency, or technical noise fluctuate in time. Therefore, the

fact that the multiparameter approach delivers the same sensitivity as the calibrated single-parameter method while avoiding additional calibration measurements is exactly one of the strenghts of our work.

To stress this important point, we have now included a new subsection in the Supplementary Information.

A. Pre-calibration measurements

In the single parameter strategy, the probe and system parameters r and η, have to be pre-calibrated. In practice, this requires collecting additional homodyne data before the phase-estimation run. This stage represents a genuine metrological cost because these extra *samples do not directly contribute to estimating the parameter of interest. To quantify the amount of required* extra *resources, we determine, from experimental measurements, the relative uncertainty on the calibration measurements of r and η as a function of the number of homodyne samples M_{calib} employed in this pre-calibration stage. The results reported in Fig.6 show that achieving a relative error, on both measurement of r and η, below 1% requires employing at least $M_{calib} = 5000$ samples. A perfect calibration is instead obtained for M_{calib} larger than $40k$. This makes explicit that, even under stable conditions, obtaining r and η values needed to retrieve the likelihood and the optimal measurement projection assumed in single-parameter benchmarking entails a non-negligible measurement cost.*

To highlight the operational consequence, we therefore re-evaluate the single-parameter quantum and classical bounds under an explicit resource accounting in which the total number of detected samples is $M_{tot} = M + M_{calib}$. As shown in Fig.3, this shifts away the experimentally evaluated variances from the ultimate QCRB computed considering M_{tot}. In particular, requiring a relative error on both r and η of the order of 1% requires an amount of calibration measurements M_{calib} that, if included in the overall resource budget when computing the bounds, washes away the quantum enhanced performances achieved in this scenario. This is shown by the fact that in this configuration the experimental points lie on the blue classical coherent bound.

By contrast, our multiparameter strategy has no separate pre-calibration stage: the relevant nuisance parameters are inferred online from the same measurement record used to estimate ϕ. In this scenario, we already include in the reported performance and in the corresponding bounds the total resource consumption. All detected samples contribute simultaneously to updating the inference model and to steering the adaptive measurement toward maximal information. This removes the structural separation between calibration and sensing that can otherwise inflate apparent performance when calibration resources are omitted, and it directly illustrates the operational advantage of a genuinely calibration-free protocol.

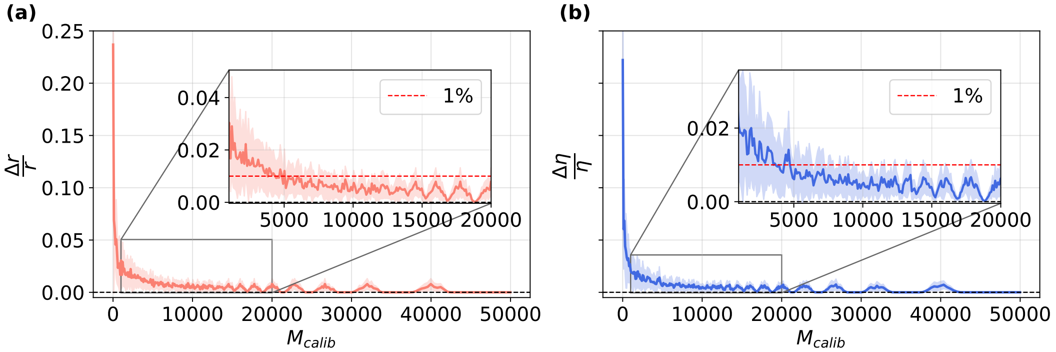

FIG. 6. **Resources for pre-calibration.** The experimental relative error of the pre-calibrated squeezing parameter r and overall efficiency *eta* values as a function of the number of processed homodyne samples M_{calib}, are reported in panel **(a)** and **(b)**, respectively. Insets highlight the 1% relative-error threshold (red dashed line). The black dashed line denotes the perfect estimate.

10) Page 6, left column, bottom section: "the multiparameter strategy requires a larger number of measurements before reaching convergence, since part of the data are used to

estimate the squeezing level of the probe". This sounds like a type of calibration for the multiparameter approach. Is it not and if not why?

The key point is that the additional measurements required by the multiparameter strategy do not constitute a separate calibration stage. They are part of the same sensing run, processed within a single unified inference model to jointly estimate the phase and the probe parameters from the measurement outcomes. This is the strength of our multiparameter strategy that does not rely on any pre-calibration, and on the opposite all measurement outcomes are counted and used consistently within the same Bayesian update. This should be contrasted with the standard single-parameter approach (used in many previous squeezed-light phase estimation experiments), where the Bayesian model is built assuming known probe and detection parameters (squeezing level and efficiency). In that setting, obtaining the required values of r and η requires an external characterization step, whose calibration measurements are typically not included in the overall metrological resource budget used to compute the ultimate precision bound and thus inflating the achieved performance.

To avoid confusion, we now rephrase the sentence in the manuscript:

> *As expected, because the multiparameter strategy jointly infers both the phase and the probe parameters from the same homodyne record, it requires a larger number of measurements before reaching convergence, since ~~part of the data are used to estimate~~ every measurement outcomes contribute to simultaneously estimate both ϕ and self-calibrate the squeezing level of the probe.*

11) Page 7, left column, middle section: "This behavior provides an experimental indication that stronger squeezing yields greater metrological gain". What is meant by metrological gain?

Here, by metrological gain, we refer to the improvement in phase estimation precision with respect to coherent states, i.e., the shot-noise benchmark for the same mean photon number. We now clarify this in the revised manuscript as follows:

> *As r increases, the variance decreases accordingly, and the gap between squeezed light estimation and the classical coherent state shot-noise benchmark widens. This behavior provides an experimental indication that stronger squeezing yields ~~greater metrological gain.~~ larger improvement in estimation precision at fixed resources.*
